# Questionnaire survey of the pan-African trade in lion body parts

**DOI:** 10.1371/journal.pone.0187060

**Published:** 2017-10-26

**Authors:** Vivienne L. Williams, Andrew J. Loveridge, David J. Newton, David W. Macdonald

**Affiliations:** 1 School of Animal, Plant & Environmental Sciences; University of the Witwatersrand, Wits, South Africa; 2 Wildlife Conservation Research Unit, Department of Zoology, Oxford University, The Recananti-Kaplan Centre, Tubney House, Tubney, Oxon, United Kingdom; 3 TRAFFIC East/Southern Africa, c/o IUCN ESARO, Hatfield, Pretoria, South Africa; National Zoological Park, UNITED STATES

## Abstract

The African lion is in decline across its range, and consumptive utilisation and trade of their body parts and skins has been postulated as a cause for concern. We undertook a pan-African questionnaire and literature survey to document informed opinion and evidence for the occurrence of domestic and international trade and consumption in African lion body parts across current and former range states. Sixty-five people from 18 countries participated in the online questionnaire survey (run from July 2014 to May 2015), with information provided for 28 countries (including 20 out of 24 countries believed to have extant populations). Respondents were experts within their professional spheres, and 77% had ≥6 years relevant experience within lion conservation or allied wildlife matters. Their opinions revealed wide sub-regional differences in consumptive use, drivers of trade, and access to lions that impact wild lion populations in different ways. Traditional medicine practices (African and Asian) were perceived to be the main uses to which lion body parts and bones are put domestically and traded internationally, and there is reason for concern about persistent imports from former lion range states (mainly in West Africa) for parts for this purpose. The domestic, rather than international, trade in lion body parts was perceived to be a bigger threat to wild lion populations. Parts such as skin, claws, teeth and bones are thought to be in most demand across the continent. The impact of international trade on wild populations was acknowledged to be largely unknown, but occasionally was judged to be ‘high’, and therefore vigilance is needed to monitor emerging detrimental impacts. Seventeen countries were nominated as priorities for immediate monitoring, including: South Africa, Tanzania, Zimbabwe, Mozambique, Zambia, Botswana, Kenya, Nigeria, and Cameroon. Reasons for their selection include: prevalence of trophy hunting, ‘hot spots’ for poaching, active domestic trade in lion body parts, trade in curios for the tourist market, and histories of legal-illegal wildlife trade. This survey, and increased incident reports since mid-2015 of lion poisoning and poaching in Mozambique, Zimbabwe and South Africa, and sporadic poaching events in Uganda and Tanzania, are signalling an escalating trend in the trade of lion products that is an increasing threat to some national populations. The evidence is sufficient to make more detailed investigation of this trade a conservation priority.

## Introduction

The African lion once occurred in 47 African countries, but is now extinct in 18 (38% of former range states) and possibly extinct in five others [1]. Of the 24 countries believed to have extant populations, three are estimated to have wild populations with ≤30 individuals and nine have wild populations of >1000 individuals [1], the latter concentrated in East/Southern Africa (S1 Table). Moreover, lion populations are presently declining in all but four range states [2].

**Fig 1:**
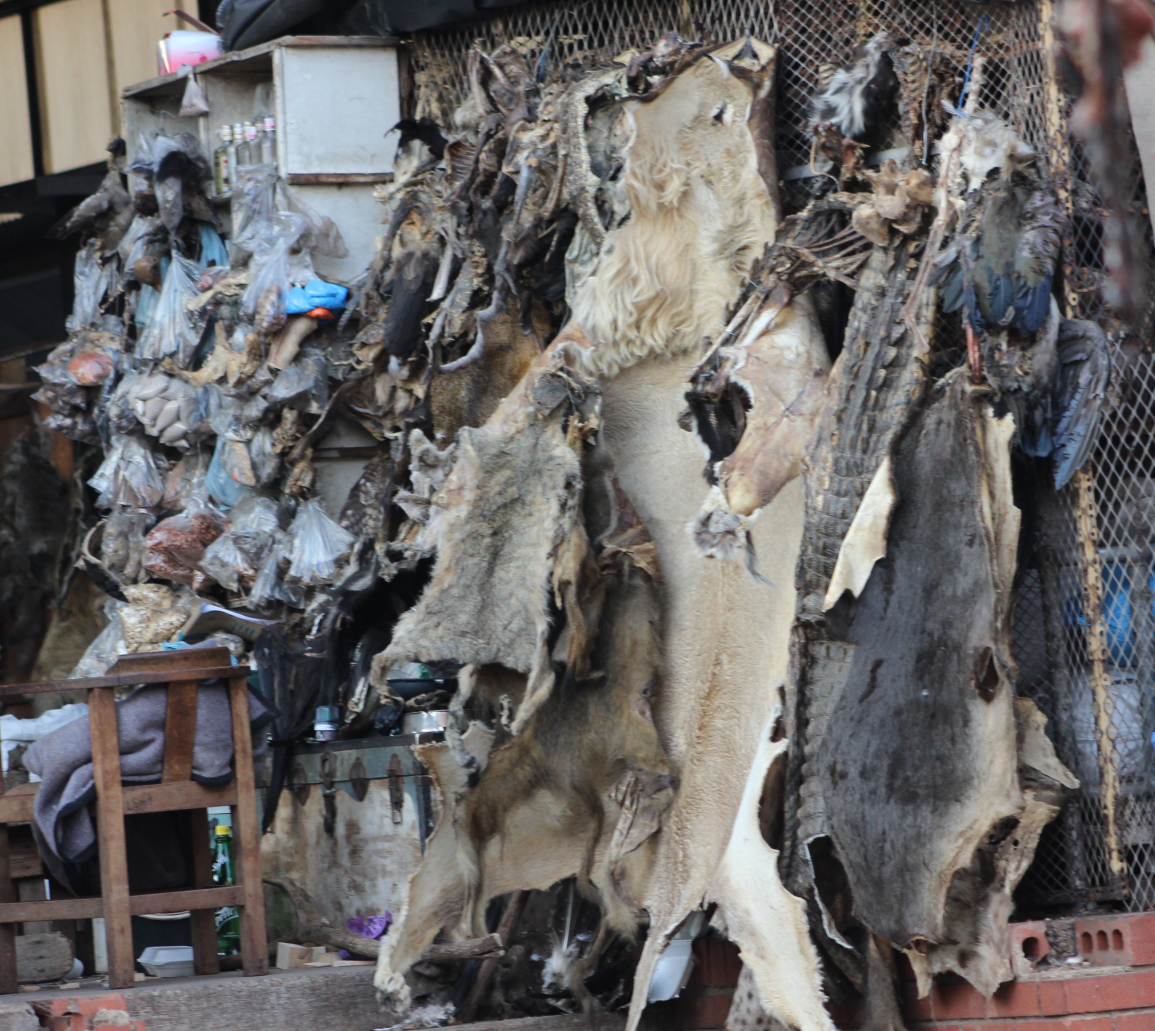
Lion skin in a Johannesburg traditional medicine market. (Copyright J. Calle).

Their decline across the continent is due to factors such as habitat loss, prey base depletions, human-lion conflict, poorly regulated sport hunting, poaching, consumptive utilisation and, to a poorly documented extent, trade of body parts and skins [2–4] (Fig 1). Consumptive use is a cause of rising concern, particularly the threat of commercial and non-commercial use and trade in lion bones and body parts for ‘zootherapeutic’ purposes (both Asian and African). This factor drew much attention at the October 2016 CITES Conference of the Parties 17 (CoP17) held in Johannesburg [2,4–6,8]. However, with the exception of the lion bone trade, published information on these practices to evaluate their importance to lion conservation are fragmented, frequently anecdotal and/or in ‘grey’ literature sources.

To address apparent information gaps, we undertook a pan-African questionnaire and literature survey in 2014/15 to document informed opinion and evidence of the domestic and international trade and consumption in wild African lion body parts across current and former range states. In addition, CITES trade data were explored to ascertain where and for how long legal trade has occurred in Africa. Our purpose was to address questions that were subsequently raised at CoP17 discussions and beyond. We asked: (i) what is known about the purposes for which lion body parts are sought after and traded, domestically and internationally? (ii) is trade perceived by expert opinion to be a substantial and current threat to lion populations and conservation efforts, and if so, why and where? (iii) do trade hotspots exist, and are these places also sources or conduits? (iv) which countries/regions should be prioritised for further investigation? and lastly, (v) insofar as it is detrimental to lion conservation, what could be done to curtail the trade? We sought answers to the questionnaire survey from people with knowledge and expertise in lion conservation, wildlife trade and law enforcement regarding inter- and intra-African use and trade in African lion body parts.

## Methods

### Questionnaire survey

An online survey was conducted between July 2014 and May 2015 using a structured semi-quantitative questionnaire with 25 questions designed and pre-tested to solicit information on knowledge, awareness and/or understanding of the use and trade in wild African lion body parts across Africa (S2 Table). Questions were in three sections: (A) participant information, (B) general use and trade of lion body parts (including generic questions on bones to permit comparisons with answers for other body parts), and (C) lion bone trade. Except where specified, questions from Section B excluded sport hunting trophies because the topic is frequently the subject of investigations (accordingly, rather than review the practice, we limited the literature survey on trophy hunting to reports of where it has occurred).

The questionnaire was created and administered using SurveyMonkey®, an online software service that provides a platform for customised surveys. The software has tools for tabulating results, generating convenient question summaries and charts, and exporting data to Excel in multiple formats for further analysis. The survey was translated into French and Portuguese for respondents in Francophone and Lusophone lion range states respectively.

We employed a ‘chain-referral’ sampling method to circulate the questionnaire among potentially suitable research participants and to recruit new respondents. The method involved sending email invitations to potential respondents from various academic, government and non-government institutions/organisations with experience and regional insights. The first wave of invitees was identified from our collective professional email lists. The invitees were asked to invite other potential research participants from their professional networks. The survey invitation was also circulated via Facebook and Twitter, which involved asking numerous relevant organisations and people with social media accounts to post a web link that directed interested parties to the survey site. Chain-referrals via these sites occurred when friends and followers shared and ‘retweeted’ the invitations. Custom URLs were specifically created for the email and social media invitations that enabled the number of informants who responded to the questionnaire via these different instruments to be tracked. Reminders and new invitations to participate in the survey were periodically sent out while the survey was open. Because the invitations were electronic and by referral, we could not monitor all who received an invitation. The answers provided by the respondents (so-called ‘self-nominated experts’) comprise the ‘informant opinions’ analysed here.

All protocols followed in this study were approved and carried out in accordance with the ethical guidelines and recommendations of the Human Research Ethics Committee (non-medical) of the University of the Witwatersrand (Protocol Number H14/03/02). Respondents were informed on the first page of the questionnaire that they could remain anonymous, and that submission of the online questionnaire would be interpreted as their consent to use the information they provided (S2 Table).

### Literature assessment

Literature was consulted to extract information on the loci of domestic/international and legal/illegal commercial/non-commercial consumptive use and trade of lions across Africa. The literature included ethnozoological accounts of wildlife utilisation (including ‘zootherapy’ and allied practices) and trade, reports of commercial and subsistence hunting and poaching, as well as lion conservation statuses and strategies that were published in peer-reviewed papers, reports, online newspaper articles, theses, and other grey literature available on websites. The publications were found using various combinations of keywords pertinent to utilisation, trade, hunting and threats at country-specific, regional and continental scales. These terms were also translated into French and Portuguese because, in addition to English, they are generally the official and/or working languages of countries with extant or possibly extinct lion populations and also the languages in which we found most reports on lion conservation, utilisation and trade to be published. While hundreds of publications in several languages were viewed in the course of the investigation, we ultimately used information from 139 sources for S1 Table [9–147]. The cited publications were mainly in English and cover a period of 98 years from 1918 to early 2016, however the majority (50%) was published between 2010 and 2016 and 34% between 2000 and 2009.

Information retrieved from this process was tabulated per African country and combined with the results from the questionnaire survey and selected CITES trade statistics derived from the CITES Trade Database maintained by UNEP-WCMC (https://trade.cites.org/) (see S1 Table). Through records of issued export/import permits, the CITES database has the best publicly available information on when and where legal trade in lions has occurred. There are, however, limitations to using the Database that we acknowledge (such as discrepancies in how countries report data to CITES, and the failure of some countries to submit reports).

### Data analysis

**Fig 2:**
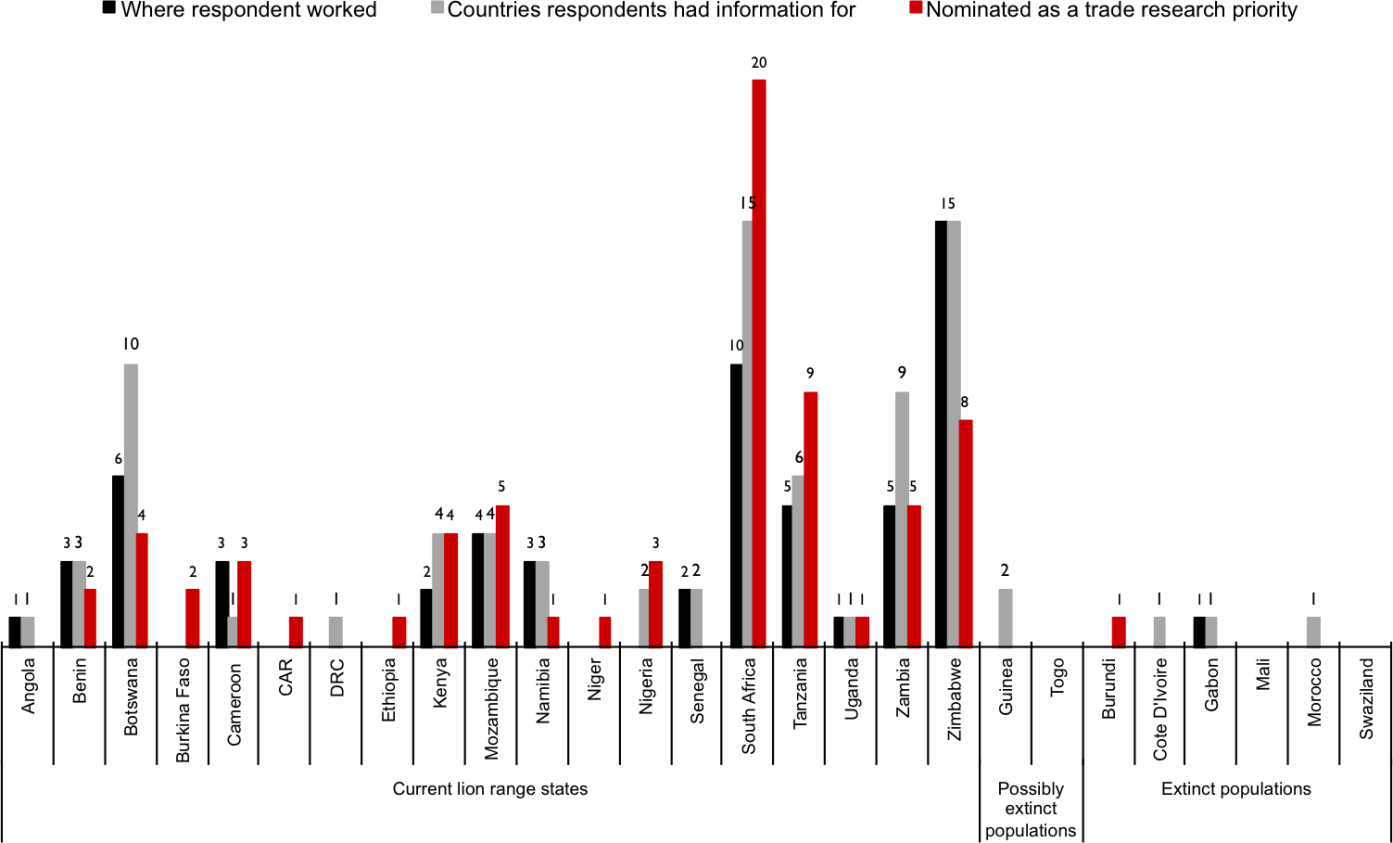
All African countries named during the survey. The legend further indicates countries (i) where respondents worked/resided, (ii) for which respondents specifically stated they had information for (includes multiple responses), and (iii) nominated as priorities for further research (includes multiple responses). Countries mentioned include lion range states with extinct and possibly extinct lion populations. Range states not mentioned during the survey are Malawi, Somalia, Sudan and South Sudan. In addition, 10% of respondents worked in Europe and the USA. (See S1 & S6 \s. for sub-region summaries).

In addition to the overall synthesis of answers to each question, most results were subdivided according to African sub-regions (Table 1) to avoid distortions due to large differences in the numbers of respondents between regions (e.g. Fig 2 later, S1 Fig). Furthermore, results from South Africa were excluded from Southern Africa and presented alongside the other sub-regions. This sub-regional approach was also taken to avoid sampling biases, and to be consistent with the treatment of the population ranges in the regional lion conservation strategies of the IUCN Cat Specialist Group [73,74] and the 2015 IUCN Red List assessment [1,3]. Note that: (i) countries in these African lion geographical sub-regions are different to the groupings defined by the United Nations [148], which has Angola, Malawi, Mozambique, Sudan, Zambia and Zimbabwe in different sub-regions; (ii) since Morocco was the only North African country mentioned in the survey, results for this former lion range state are mentioned only in the text and not in a separate sub-regional result. The respondents’ answer to Question 7 (‘lion range state(s) the answers were for’) and/or the comment sections of other questions (where clarity on the place to which the answers pertained was requested) determined the sub-region to which answers were assigned, and answers were manually reassigned to sub-regions accordingly.

**Table 1:** Sub-regional division of African lion range states with extant, extinct and possibly extinct wild populations. Countries in parentheses were not mentioned by any respondent in the survey.

African sub-regions [1,73,74] ^a^	Extant lion populations (n = 24)	Extinct lion populations (n = 18)	Possibly extinct lion populations (n = 5)
**Southern**	Angola, Botswana, Mozambique, Namibia, South Africa, Zambia, Zimbabwe (Malawi)	Swaziland [1] ^b^ (Lesotho)	
**East**	Ethiopia, Kenya, Tanzania, Uganda (Somalia)	Burundi (Djibouti, Eritrea)	(Rwanda [1] ^c^)
**West**	Benin, Burkina Faso, Niger, Nigeria, Senegal	Cote d’Ivoire, Mali (Gambia, Guinea Bissau, Mauritania, Sierra Leone)	Guinea, Togo (Ghana [1] ^d^)
**Central**	Cameroon, Central African Republic, Chad, Democratic Republic of Congo (South Sudan, Sudan)	(Congo)	Gabon [149] ^e^
**North**		Morocco (Algeria, Egypt, Libya, Tunisia, Western Sahara)	

^a^ Sub-regional classification of lion range states derived from Table 1 in [1]. Additional information obtained from the other reference numbers listed in the table

^b^ extirpated but reintroduced

^c^ recently reintroduced

^d^ reports in Mole National Park

^e^ Camera trap footage captured images of a single male lion in south-eastern Gabon in early 2015. This was the first sighting of a lion in Gabon in 20 years. As only a single lion has been sighted to date, one cannot consider this to indicate the presence of a lion ‘population’ *sensu stricto*, hence lions are treated here as probably functionally extinct in Gabon.

South Africa was treated separately to Southern Africa because: (i) every wild lion population in the country is growing [2]; (ii) the captive lion population there is >50% larger than the wild population [5]; (iii) <5% of lions that are hunted in South Africa are of wild origin [5,13,14], hence most trophy hunting targets lions bred in captivity; (iv) all wild populations are in fenced reserves [2]; (v) incidents of illegal offtake and poaching in national and private reserves are rare and not believed to be a notable contributor of illegally obtained body parts in the country [5]; (vi) in 2015 we published a major study documenting the South African trade in lion bones and body parts [5–7]; and (vii) our detailed study of South Africa generated sufficient circumstantial evidence on trade in wild animals to suggest the necessity for detailed lion bone and parts trade studies in various southern and eastern African countries–by their nature requiring separate effort. Furthermore, since most respondents had information for South Africa (Fig 2), the results would have obscured those for other Southern Africa countries where some monitored populations are in decline (even though the sub-regional population is increasing overall), where most sport hunting involves wild-origin lions, where some populations are not in fenced reserves, and where poaching and *“indiscriminate retaliatory or pre-emptive killing to protect humans and livestock”* [2] is fairly commonplace.

Respondent confidentiality was maintained by using a unique identifying number whenever they were quoted (e.g. Resp’t #1). Since respondents frequently worked in, and/or had information for, multiple countries and/or sub-regions, and several questions required multiple responses, the total response percentages for some questions exceeded 100%. The comment sections of selected questions gave respondents the opportunity to elaborate on their answers.

Questions 12 and 13 originally required answers for 11 and 12 types of uses respectively. However, to simplify the results several of these were merged to create six and seven use categories, respectively. The response rates to each of these revised use categories was manually recalculated. The use types combined were: (i) ‘crafts’ with ‘curios’; (ii) ‘traditional attire’ with ‘decorative’; (iii) ‘African traditional medicine’ with ‘rituals’ and with ‘magic/witchcraft/supernatural’ into a use type called ‘zootherapeutic: African’. Question 14 originally had a source category for ‘Taxidermists’, but this category was excluded in the analyses since it represented a secondary source for body parts that could not be classified as ‘captive’ or ‘wild’. Quantitative answers to Question 15 on the perceived legality of trade were omitted because our wording of the question did not evince answers amenable to analysis, but anecdotes from the comment section are retained in S1 Doc.

Recent and historical information collected through the literature and questionnaire surveys was considered cumulative and complimentary. Hence each new national record was interpreted as evidence that a category of legal/illegal consumptive use/trade had occurred in that country in the past and/or present. Thus gaps in the information record must not necessarily be interpreted as signifying evidence for the absolute absence of illegal trade and/or use type within the respective consumer populations. Furthermore, wherever information on a purpose for trade received from respondents could not be corroborated with the available literature, we noted these differences and did not treat them as being contradictory. In terms of priority setting for future research to evaluate the impact of consumptive use on national lion populations, the answers to Question 25 (‘which countries are the most important to investigate’) were supported (but not weighted) with more recent information on, for example, lion population statuses, duration of CITES trade, and evidence for illegal trade and potentially detrimental consumptive activities.

For brevity in the questionnaire, and accordingly continuity in the results, we simplistically referred to ‘Asia’ and/or ‘Asian’ in certain contexts (e.g. traditional medicine). Strictly speaking however, the ‘Asian’ sub-regional focus is on East-Southeast Asia–which includes the four key destination countries for lion bone exports, namely China, Laos, Thailand and Vietnam [8].

The results are arranged in themes corresponding to (i) respondent demographics (Questions 3–10), (ii) the foci of the pan-African survey for lion body parts (sometimes including bones) (Questions 11–14,16,17), (iii) the trade in lion bones (Questions (12–23), (iv) a sub-regional summation of trade, (v) nominated priority range states (Questions 24–25), (vi) literature summation, and (vii) African healing practices and the lion trade.

## Respondent demographics and extent of knowledge

Sixty-five people working/residing in 18 countries (15 African) participated in the survey, with 80% answering all 25 questions (Table 2; Fig 2; S1 Fig). The majority worked from Southern Africa (61%, including South Africa) and completed the survey in English (91%) (Table 2). Most respondents said they had information for Southern Africa (63%, including South Africa), followed by West and East Africa (15% and 13% respectively, Fig 2; S1 Fig); hence, regional information levels were lowest for Central Africa. Although initially saying they had information for 19 countries (Fig 2), information for 28 countries was received via the comment sections of some questions (20 out of 24 countries believed to have extant wild lion populations; five with extinct populations; three with possibly extinct populations) (Table 1; S1 Table). Lion range states not mentioned were Malawi, Somalia, Sudan and South Sudan, but then no reliable estimates for the sizes of the Somali and Sudanese lion populations are available either [2].

**Table 2:** Number of survey respondents and questionnaire completion rate.

	Number of respondents	Completion number	Completion rate	Language proportion
**English**	59	46	78%	91%
**French**	3	3	100%	5%
**Portuguese**	3	3	100%	5%
**Total**	65*	52	80%	100%

* Respondents accessed the questionnaire via (i) chain-referral email invitations = 75% (n = 49), and (ii) Facebook = 25% (n = 16)

**Fig 3:**
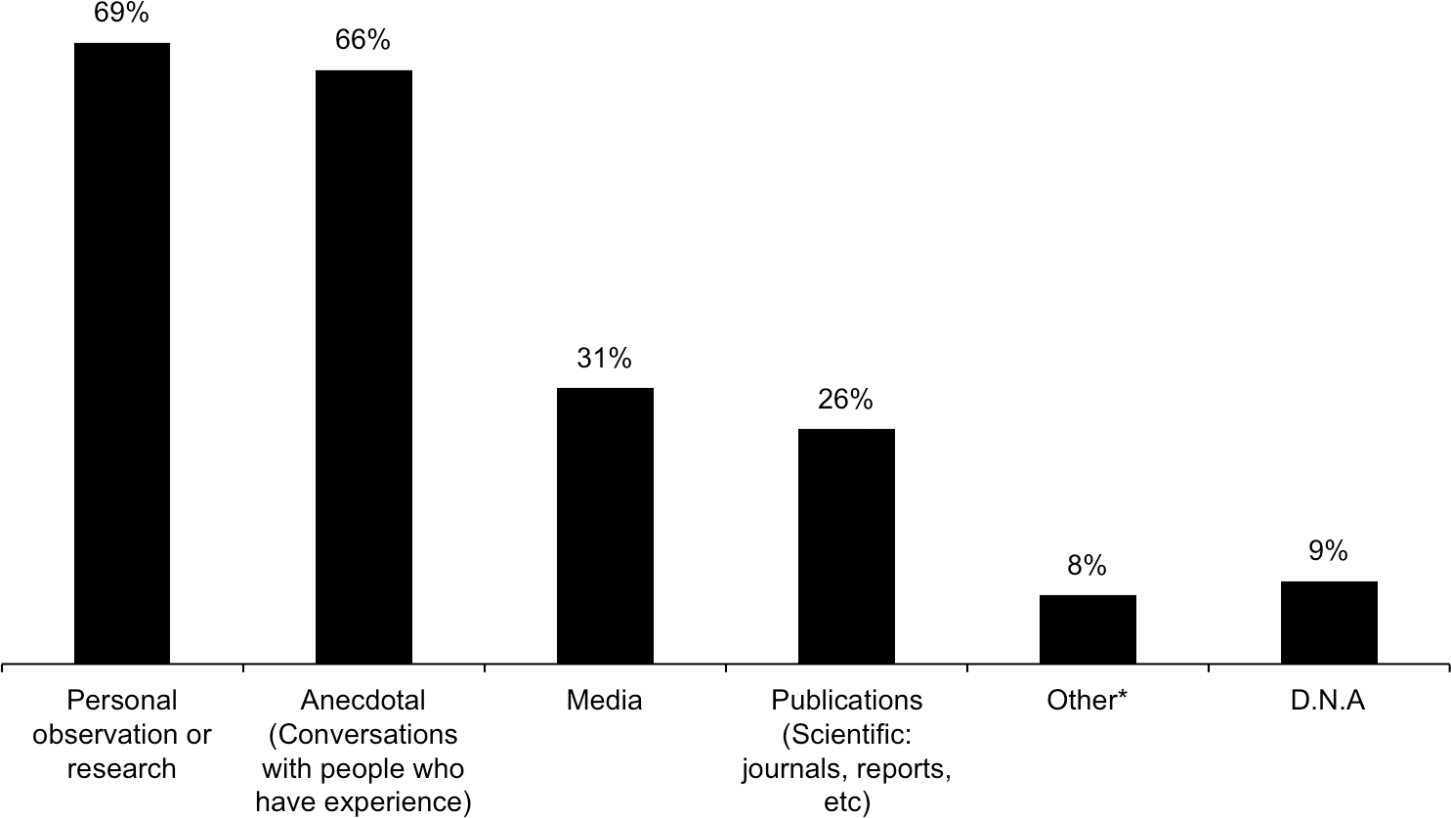
Respondents’ sources of information for the trade in lion products (includes multiple responses). (* Other includes: permit applications, rumour, covert operations; D.N.A. = Did Not Answer) (Answers correspond to survey Question 9).

The respondents’ expertise was mainly within the broad domain of carnivore conservation (44%, including research, monitoring, management, human-wildlife mitigation), and 31% within general wildlife conservation (including conservation biology, ecology, management of wildlife that may include lions) (S2 Fig). They included academics (22%) and people from government institutions (14%, including law enforcement). Two respondents chose not to identify themselves, but all the others provided sufficient information (name, email, job description and/or years of experience) that identified them as credible self-selecting experts within their professional capacities. Respondents with ≤5 years of experience (23% of respondents) tended to be affiliated to a university and had information based on personal research observations. However, 77% of respondents had ≥6 years of appropriate experience within lion conservation or allied wildlife matters, and 58% had ≥11 year’s experience (S3 Fig). In terms of information sources, 69% said their knowledge was from personal observation/research, 66% also had anecdotal information, 31% had information obtained from the media, and 26% from publications (Fig 3).

## Pan-African trade in lion body parts

Since the results are the outcome of a questionnaire survey, they reflect the perceptions of the respondents (except where specified) and not necessarily the actual state of affairs.

### Awareness of body parts traded

**Fig 4:**
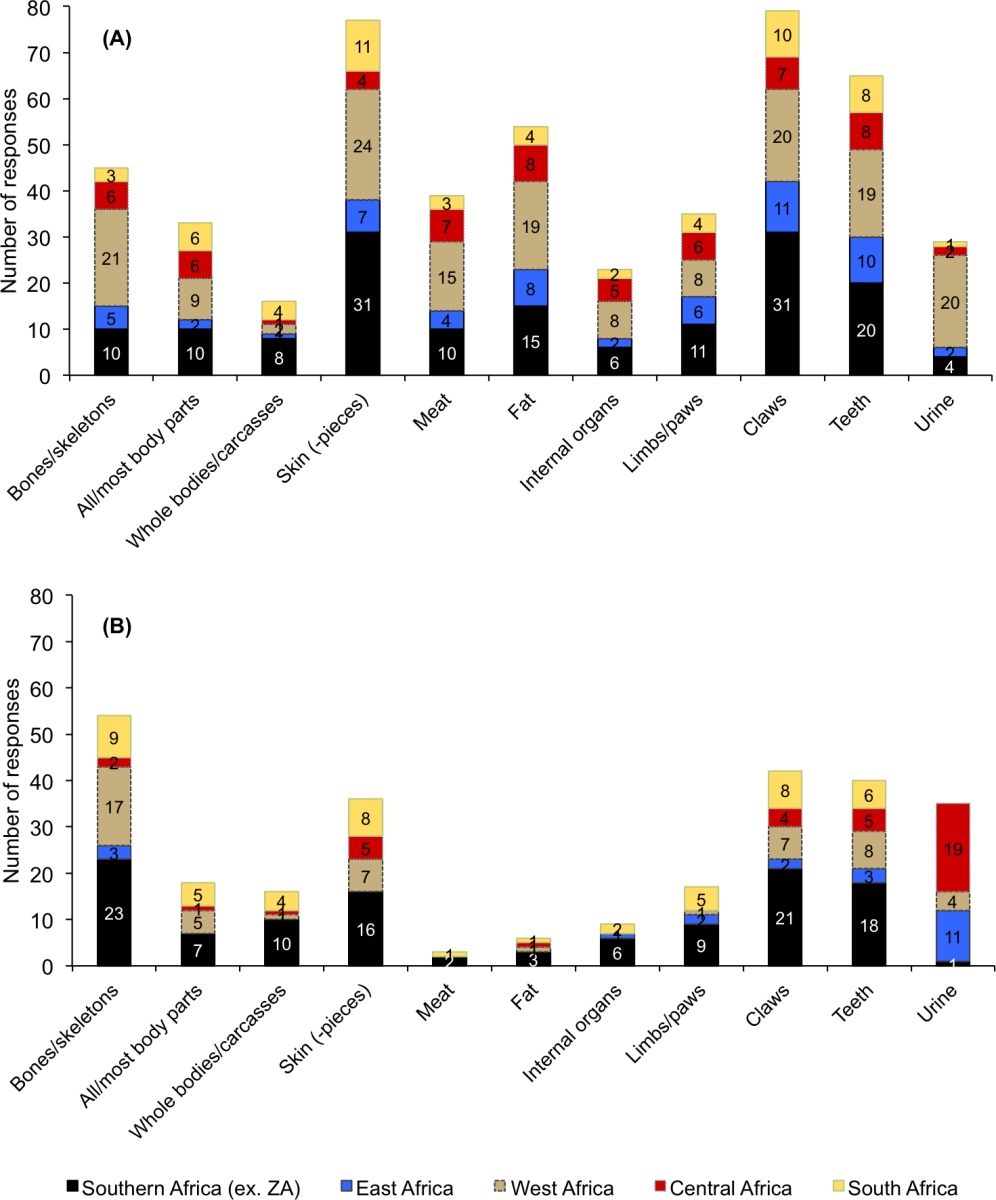
**Informant awareness of use/trade in specific lion body parts, (A) domestic/local, and (B) international.** Multiple responses for body parts and sub-regions were allowed. (Ex. ZA = Excludes South Africa) (Answers correspond to survey Question 11) Axes for both graphs to the same scale.

Respondents were generally more aware of domestic use of certain lion body parts compared to an international trade thereof (Fig 4A and 4B, Question 11), with the exception of lion bones (n = 45 domestic trade, n = 54 international trade). Skin, claws, teeth and bones were most frequently thought to be traded overall (domestic and international combined). Sub-regional differences at the domestic and international levels were also evident (Fig 4); for example, domestic use of ‘lion’ urine was more commonly listed for West Africa (n = 20; Fig 4A), but respondents perceived there to be more international trade in it from Central and East Africa (n = 19 & 11; Fig 4B). Sub-regional patterns are summarised later in Table 3 and S1 Doc. In the comments for Question 11, a respondent with information for Morocco (Resp’t #24) wrote that there was domestic and international trade in lion skins there, and that the skins traded domestically within the country originated from outside the country.

**Table 3:** Comparative sub-regional summary of the most responses per category per question. Answers are for informant opinions. Numbers in brackets with ‘%’ are for single answer questions; the other numbers in brackets are for questions where multiple answers could be selected and the body parts are listed in descending order of responses per category.

Questions:	South Africa	Southern	East	West	Central	Total
**Awareness of trade:** **domestic** (Fig 4A)	Skin (11); claws (10); teeth (8)	Skin & claws (31 each); teeth (20); fat (15)	Claws (11); teeth (10); fat (8); skin (7)	Skin (24); bones (21); claws & urine (20 each)	Fat & teeth (8 each); claws & meat (7 each)	Claws (79); skin (77); teeth (65); fat (54)
**Awareness of trade:** **international** (Fig 4B)	Bones (9); skin & claws (8 each); teeth (6)	Bones (23); claws (21); teeth (18); skin (16)	Urine (11); teeth & bones (3 each)	Bones (17); teeth (8); claws & skin (7 each)	Urine (19); teeth & skin (5 each)	Bones (54); claws (42); teeth (40); skin (36)
**Reasons for trade:** Domestic* (Fig 5A, S1 Doc)	ZooTh. (9; fat, claws, bone) Tr. att. (8; skin, teeth) In. gen (7; bone; skin, claws)	Craft/curio (18; claws, teeth) ZooTh. (17; claws, skin, fat) In. gen. (15; skin)	ZooTh. (12; fat, claws, bones) Tr. att. (7; skin, claws, teeth) Stat. sym. (7; skin, claws)	ZooTh. (11; skin, claws, teeth) Stat. sym.; In. gen.; Bushmeat (5 each; variety of parts)	ZooTh. (11; teeth, claws, skin, bone)	ZooTh. (37; claws, fat, skin) Tr. attire (29; skin, teeth) In. gen. (28; skin, claws, All)
**Reasons for trade:** International* (Fig 5B, S1 Doc)	ZooTh. (22 Asian + Afr.; bones, All, claws, teeth, skin, fat) Craft/curio (11; bone, All, skin) Tr. att. (9; skin, teeth)	ZooTh. (21Asian + Afr.; bones, claws, teeth) In. gen. (13; bone, skin, All) Craft/curio (12; teeth, claws, skin)	ZooTh. (16 Asian + Afr.; bone, teeth, claws) Tr. att. (8; skin, teeth) In. gen. (8; skin, All)	ZooTh. (15 Asian + Afr.; all, bone, teeth) Stat. sym. (6; skin, teeth) In. gen. (5; All, skin, bone)	ZooTh. (11 Asian + Afr.; bone, All, teeth) In. gen. (5; skin, bone, All)	ZooTh. (44 Asian + Afr.; bone, All, teeth) In. gen. (24; skin, bone, All)
**Sources:** **body parts** (Fig 6A)	Captive: trophy & Captive: not hunted (7 each)	Wild: problem lion (16); Wild: poaching (14)	Wild: poaching (7); Wild: problem lion (6)	Wild: poaching (6); Wild: problem lion (4)	Wild: trophy & Wild: poaching (2 each)	Wild: poaching (33); Wild: problem lions (30)
**Sources:** **bones** (Fig 6B)	Captive: trophy (10); Captive: not hunted (8)	Wild: trophy (15); Wild: poaching (14)	Wild: poaching (6); Wild: problem lion (5)	Wild: poaching (7); Wild: trophy (4)	Wild: poached (2)	Wild: poaching (35); Wild: trophy hunted (28)
**Impact of trade:** **body parts; domestic** (Fig 7, S4 Fig)	Low impact (33%); Unknown (25%)	Low impact (33%); Unknown (27%)	Medium impact (32%); Unknown (32%)	Unknown (40%); Low impact (32%)	Unknown (39%); Low impact (33%)	Low impact (32%); Unknown (32%)
**Impact of trade:** **body parts; international** (Fig 7, S5 Fig)	Unknown (75%)	Unknown (48%) High impact (21%)	Unknown (79%)	Unknown (69%) High impact (27%)	Unknown (83%) High impact (17%)	Unknown (65%) High impact (18%)
**Impact of trade:** **bones; domestic** (Fig 7, S4 Fig)	Low impact (45%); Unknown (27%)	Low impact (45); Unknown (28%)	Low impact (41%) Unknown (38%)	Unknown (50%); Low impact (45%)	Low impact (53%); Unknown (45%)	Low impact (47%); Unknown (35%)
**Impact of trade:** **bones; international** (Fig 7, S5 Fig)	Unknown (50%) Low impact (25%)	Unknown (27%) Low impact (25%)	Low impact (34%) Unknown (28%)	Low impact (41%) Unknown (28%)	Low impact (41%) Unknown (31%)	Low impact (33%); Unknown (30%)

* Abbreviations for answers to the question: ZooTh = Zootherapeutic; Tr. att. = Traditional attire; In. gen. = Income generation; Stat. sym. = Status symbol; All = All body parts; Afr. = Africa

### Reasons for trade, and trading partners

**Fig 5:**
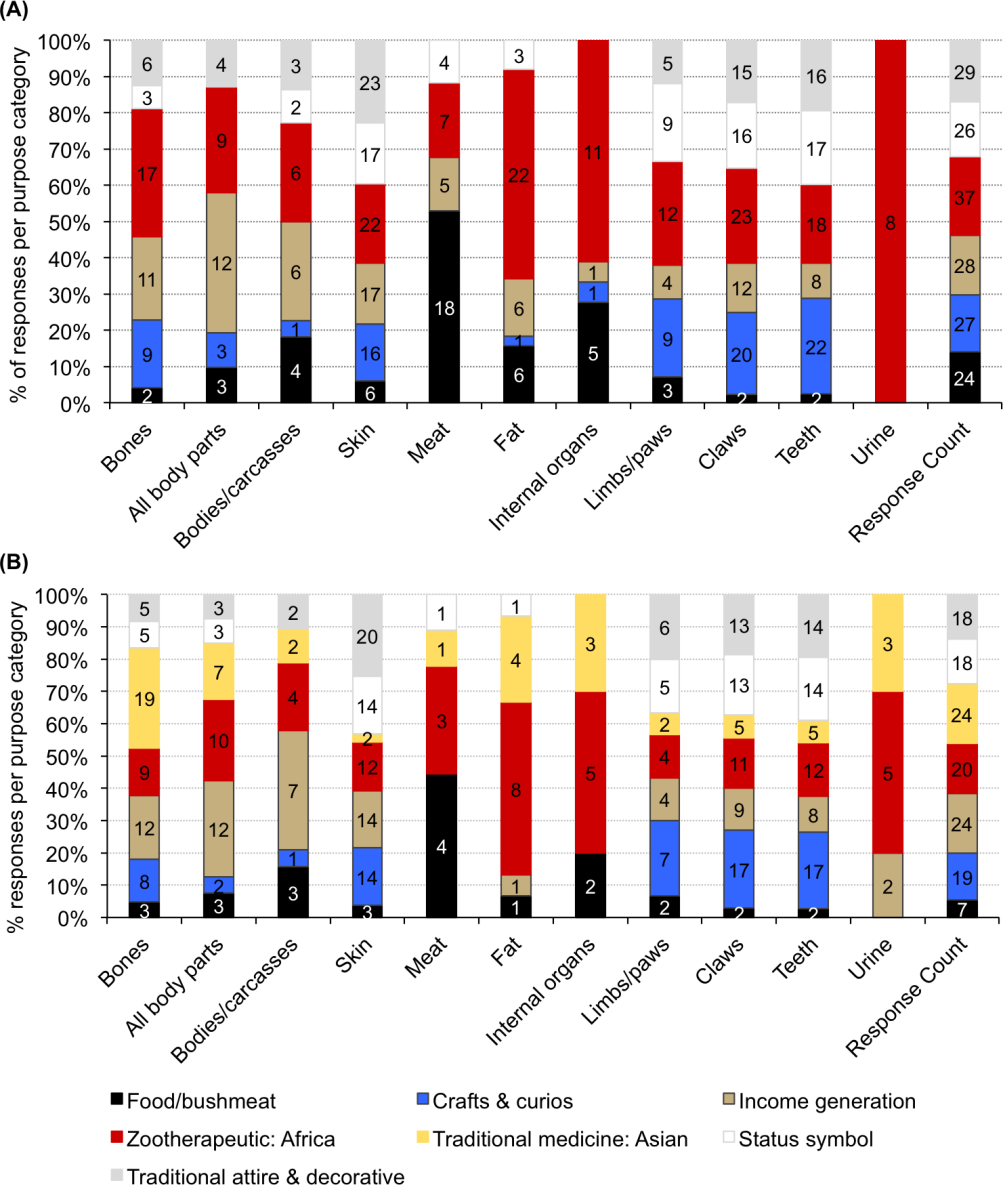
**Informant opinion of why various lion body parts are sold/used, (A) locally/domestically and (B) internationally.** The number (n) of responses is in the histogram blocks (except for ‘Response count’, which equals the total number of respondents per category), whereas the y-axis indicates the response proportions. (Answers to Fig 5A correspond to Question 12, see S4A–S4E Fig for sub-regional results. Answers to Fig 5B correspond to Question 13, see S4A–S4E Fig for sub-regional results).

African zootherapeutic practices (including traditional medicine, magic, ‘witchcraft’, rituals) (n = 37 responses; 17%) were believed to be the main reasons why lion body parts are used and traded domestically (Fig 5A, ‘Response count’ column). The parts said to be most frequently used in these practices were claws (n = 23), fat and skin (n = 22 each), and teeth (n = 18 each). Incorporation of parts into traditional attire (n = 29) (usually skin, teeth and claws) (Fig 5A) was also perceived to be prevalent. While most body parts were thought to be used for domestic income generation, the skin was most often mentioned for this purpose (n = 17) (however, most purpose categories could be considered ‘income generation’ if the parts involved are sold). Of all the body parts in domestic use/trade, the skin had the most total mentions across the combined spectrum of purposes (n = 101 total mentions; column 4), followed by claws (n = 88) and teeth (n = 83) (Fig 5A). Sub-regional patterns in domestic use/trade are summarised in Table 3 and in shown in S1 Doc.

Like the domestic trade, informant opinion was that the international trade was mostly for parts used for zootherapeutic purposes (n = 44 for African and Asian combined, 34% responses)–particularly Asian (n = 24), and to a lesser degree African (n = 20) (Fig 5B). But whereas the Asian trade was said to involve mainly bones (n = 19), pan-African international trade in body parts for African zootherapeutic practices was perceived to involve mostly skin and teeth (n = 12 each), claws (n = 11), all body parts (n = 10) and then bones (n = 9) (Fig 5B). The prevailing respondent opinion was also that trade in lion claws and teeth were mainly for the craft and curio market (n = 17 each), which would result in these parts being sold to international tourists. The pan-African skin trade, however, was mainly perceived to be for traditional attire and decorative purposes (n = 34) (Fig 5B), which corresponds with our recent personal observations of lion skins sold in traditional South African markets (Fig 1) and for which there is alleged to be an active trade within southern African involving syndicates [V.L. Williams, pers. obs.].

Of all the body parts believed to be traded internationally, skin had the most mentions across the combined spectrum of purposes (n = 79 total), followed by teeth (n = 72), claws (n = 70), and bones (n = 61); this trade in body parts is thought, by our respondents, to be mostly intra-African, with the exception of bones to East-Southeast Asia. Sub-regional patterns in international use/trade are summarised later in Table 3 and in S1 Doc. The literature does not specifically contradict the views of the respondents, but there are few records by which to assess the occurrence of trade (especially illegal trade, and the curio market). There is, however, more published information on domestic uses for body parts (particularly traditional medicine) compared to the international trade thereof.

Four respondents elaborated on the international destinations for lion parts in the comments associated with Question 13 with the following:

(i) Resp’t #39: “*All African lion range states are increasingly involved in various forms of trade in lion body parts*. *Tanzanian hunting companies have recently been approached by individuals to sell them bones from trophy hunted lions for $1*,*000 per skeleton*. *Lions killed as "problem" animals in retribution killings have many body parts ending up on commercial markets all over Africa*. *Much of this trade is local "cultural"*. *Some of the trade involves local sellers and international tourists buying souvenirs at local markets*. *Organized international trade with legal permits seems to be limited to South Africa*. *There are indications that illegal trade–by Vietnamese and Chinese nationals–occurs in very many nations in Africa*. *The extent of this illegal trade is difficult to determine as the focus of control efforts is always on ivory and rhino horn*.*”* [$ price quoted in September 2014]

(ii) Resp’t #10: “*Benin to Gabon (proof from arrest); Benin to Asia (bone; suggested by protected areas personnel); Nigeria to USA (proof from arrest in US*, *but body parts may have come from outside Nigeria); Cameroon to Nigeria (intended; proof from arrests in Cameroon [LAGA report]); Benin/Burkina Faso to Cote d'Ivoire (indicated to me by traders); Benin/Burkina Faso to Senegal (indicated to me by traders); Benin/Burkina Faso to Guinea (indicated to colleague by traders)*.*”*

(iii) Resp’t #4: “*There may be other reasons that I cannot verify (traditional medicine or ritual) that lion parts traded from Zambia are used in other countries*. *Bones*, *skins*, *and presumably all body parts have been confiscated from Congolese poachers in Zambia*. *Chinese living in Zambia have on numerous occasions attempted to solicit local villagers for lion bones and carcasses (from legal sport hunting remains) and lion "bodies" (presumably including skin since hunting has been closed)*.*”*

(iv) Resp’t #12: *“international tourists* [of unknown nationality] *buy skins in Pemba* [Mozambique] *on occasion”*. (This concern about skin, claws and teeth being sold as curios and jewellery to tourists, presumably without the requisite CITES permit, was reiterated by a Botswana respondent [Resp’t #5] who indicated that there was a market for jewellery to international travellers.)

International trade with African countries was most often stated by respondents to occur between current and former lion range states in West Africa–in particular, Benin was said to be a source of lion body parts received by Niger, Nigeria, Gabon, Cote d’Ivoire, Senegal and Guinea, whereas Burkina Faso was the origin of parts received by Benin, Cote d’Ivoire, Senegal and Guinea [S3 Table]. Togo (where lions are possibly extinct) was also listed as a destination. With the exception of Benin, lions are rare or extinct in these West African countries. In Central Africa, Cameroon was said to be the origin of parts received by Benin and Nigeria, and Gabon [S3 Table]. There was less frequent mention of trade between countries in East and Southern Africa, presumably because lion populations there are larger and local markets are supplied from within–but East and Southern Africa were believed to be more likely than other sub-regions to supply lion parts to countries in East-Southeast Asia or to Asian nationals living within a particular African country (e.g. bones and other body parts sold to Chinese nationals living in Zimbabwe, Zambia and Uganda). Also in Southern Africa, every lion range state (with the exception of Malawi, but especially South Africa, Zimbabwe and Mozambique) was reported to be a source country for parts traded internationally. The trade links reported above by the survey respondents are summarised in S3 Table with additional information included from the literature and CITES trade reports.

### Wild sourced or captive produced?

**Fig 6:**
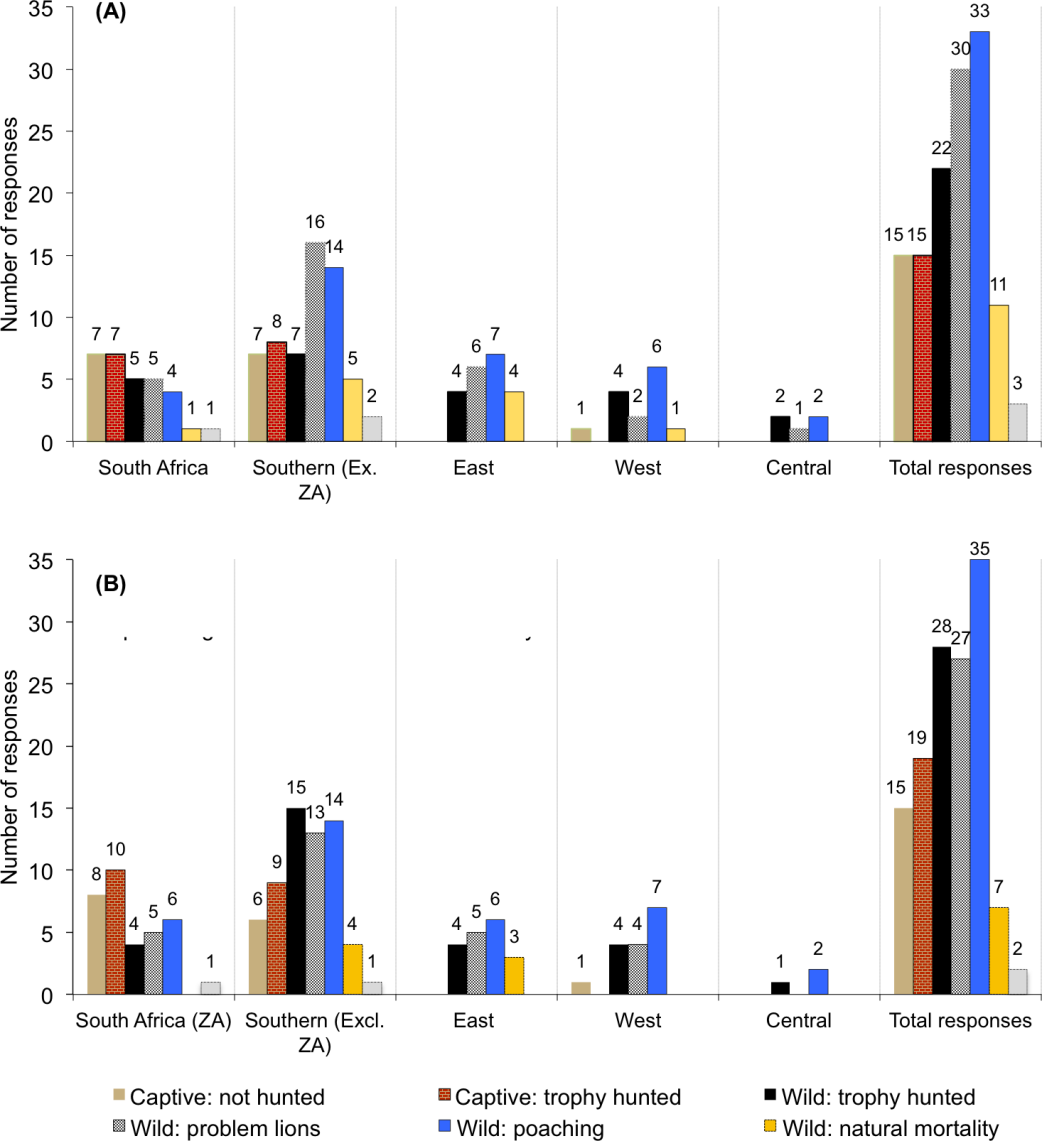
**Sources for (A) body parts (excl. bones) and (B) bones, in Africa, based on the number of responses per category.** Selection of multiple sources was allowed. (Answers correspond to survey Question 14) (Excl. ZA = excluding South Africa).

Lion body parts were mainly perceived to be obtained from wild lions that are poached (n = 33, 24% of responses) or killed due to human-lion conflicts (so-called ‘problem lions’) (n = 30, 22%) (Fig 6A, ‘Total responses’; Table 3). Poaching was considered to be the main source of body parts in East, West and Central Africa, but not Southern and South Africa (Fig 6A). Captive lions in some Southern African range states (especially South Africa, but not Mozambique) are an alternative to wild-sourced lion parts given the size of the captive breeding industry (although the rise in incidents of lion poisonings on private property in South Africa since 2015 means that ‘captive: poached’ could be added as a source category). However, ‘problem lions’ were viewed as being the major source of parts traded in other Southern African range states (Fig 6A). Overall, these results are not dissimilar for lion bones (Fig 6B, discussed later), except that trophy hunted wild lions were believed to be a bigger source for bones in Africa than problem lions (Table 3). Literature on this matter, while fragmented, is in accord with these results, but more information on actual levels of specimen origin are needed.

‘Road-kill’ and/or animals found dead in the bush were listed as alternative sources of lion body parts from Zimbabwe; Resp’t #18 wrote: “*…many times the body parts are taken* [from animals hit on the road or natural mortalities] *for use locally in traditional medicine but also for trade…this is normally done at a local level and then filters up to international trade occasionally*”. Instances in East Africa where the killing of ‘problem lions’ appeared to be a front for illegal hunting, lead to one respondent recommending that the pretext for conducting problem lion hunts should be scrutinised.

### Impact of trade

**Fig 7:**
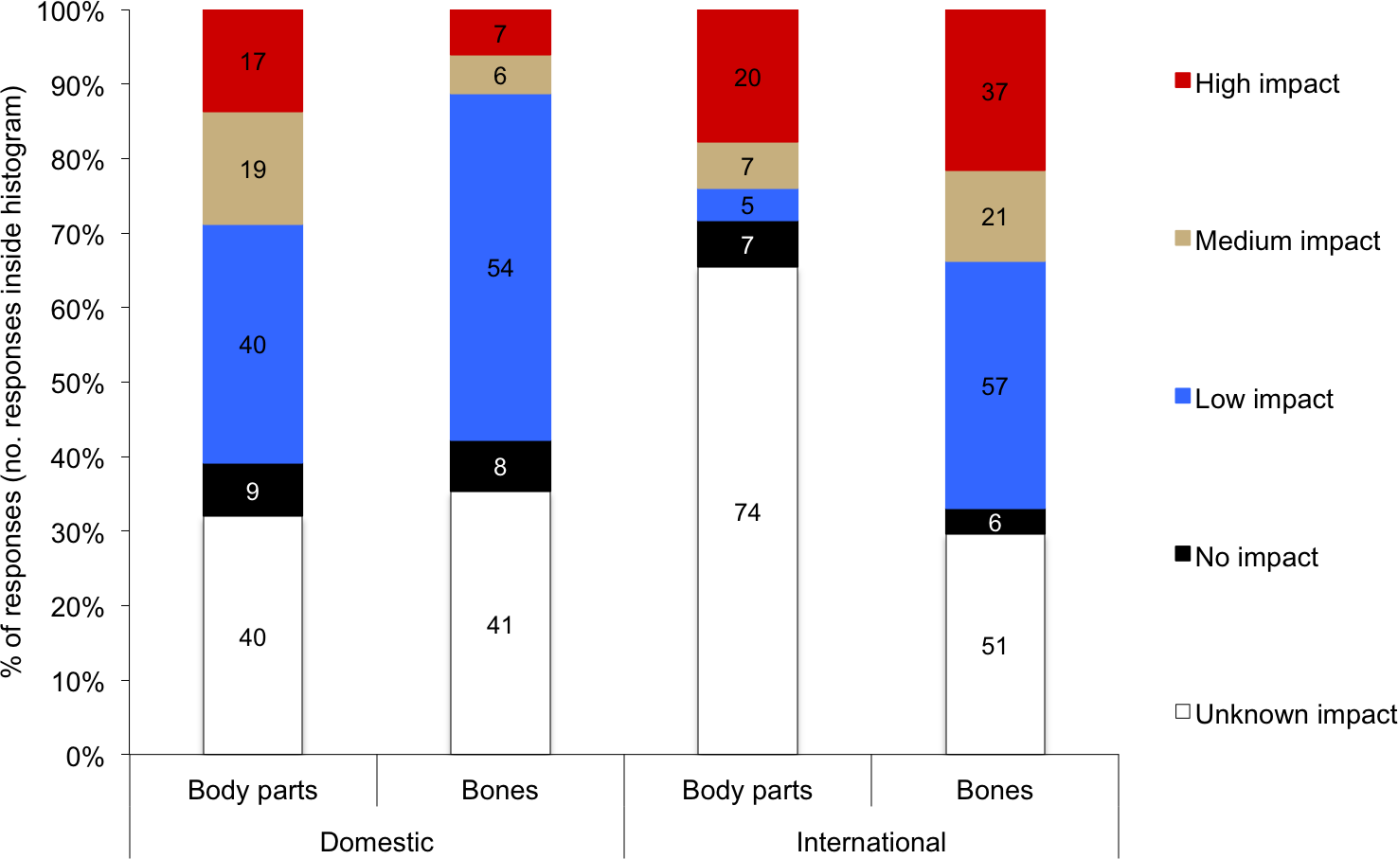
Informant opinion of the impact that domestic and international trade in body parts and bones is having on wild lion populations throughout Africa. The Y-axis represents the percent responses per region, whereas numbers inside the histogram blocks are the number of responses. (See S4 Fig and S5 Fig for domestic and international, as well as sub-regional and country-specific results) (Answers correspond to survey Questions 16 and 17; Total responses are greater than the number of respondents because the questions’ comment sections permitted people to elaborate on the countries/regions that their answers pertained to).

The timing of this study was pertinent to the results on the impact of trade. In 2015, informant opinion was that, overall, the *domestic* trade in lion *body parts* (excluding bones, which is discussed later) was having little (‘low’) to ‘no impact’ on wild lion populations on the continent (n = 9+40, 39% of respondents, ‘Total responses’ in Fig 7)–however these proportions varied across the sub-regions (see Table 3; S4 Fig). Domestic trade in body parts was perceived to be having a lower impact in South and Southern African wild populations than on wild lions in East and West Africa (given infrequent incidents of poaching of wild lions in South Africa), but there was also overall uncertainty on the impacts across Africa (32% of total responses) (S4 Fig). For the specific countries mentioned, six countries had at least one nomination each for ‘no impact’ (Botswana, Mozambique, South Africa, Tanzania, Zambia, Zimbabwe), and 13 countries had at least one nomination each for ‘high impact’ (Benin, Botswana, Burkina Faso, Cameroon, Guinea, Mozambique, Namibia, Nigeria, Senegal, South Africa, Tanzania, Zambia, Zimbabwe) (S4 Fig). These results are not markedly different for lion bones (Fig 7, discussed later), with similar proportions for ‘unknown’ impact but with the perception being that the domestic lion bone trade was having less of an impact on wild populations compared to other body parts (Table 3). Since 2015, however, incident reports of poaching in Southern Africa have escalated. Information from grey literature (such as media reports) is indicating that the impact of consumptive use on wild lion populations is rising, possibly in relation to domestic markets and/or the demand from Asia (including bones, teeth and claws), and warrants investigation.

In contrast to the domestic trade, the impact of the *international* trade in lion *body parts* (excluding bones, which is discussed later) was mostly nominated to be ‘unknown’ (n = 74, 65% of responses, Fig 7; ranging from 83% in Central Africa to 48% in Southern Africa, S5 Fig). However, where respondents said that international trade was having some impact, there were more nominations for this impact being high–especially in West and Southern Africa (e.g. Nigeria, Botswana and Zimbabwe) (S5 Fig). Other countries receiving at least one nomination each for high impact were Benin, Burkina Faso, Cameroon, CAR, Guinea, Mozambique, Namibia, Senegal and Tanzania, whereas countries with a nomination for ‘no impact’ were the same as for the domestic trade (S5 Fig). These results differ notably from the perceived impact that the international lion bone trade was having (Table 3). There was a lower rate of ‘unknown impact’ responses (30% total responses), and a more prevalent belief that international trade was having a high impact somewhere in every range state, especially in Southern Africa (Fig 7, discussed later).

Respondents did not distinguish the impact to a national population from the impact to a local sub-population in their study area when answering the questions. Hence, while seemingly contradictory at times (e.g. countries nominated as both ‘no impact’ and ‘high impact’), these results could indicate varying degrees of impacts to lions across a range state and sub-region rather than conflicting answers. That the international trade impacts are considered to be mostly unknown indicates, at the least, a need for vigilance.

## Pan-African use and trade of lion bones

**Fig 8:**
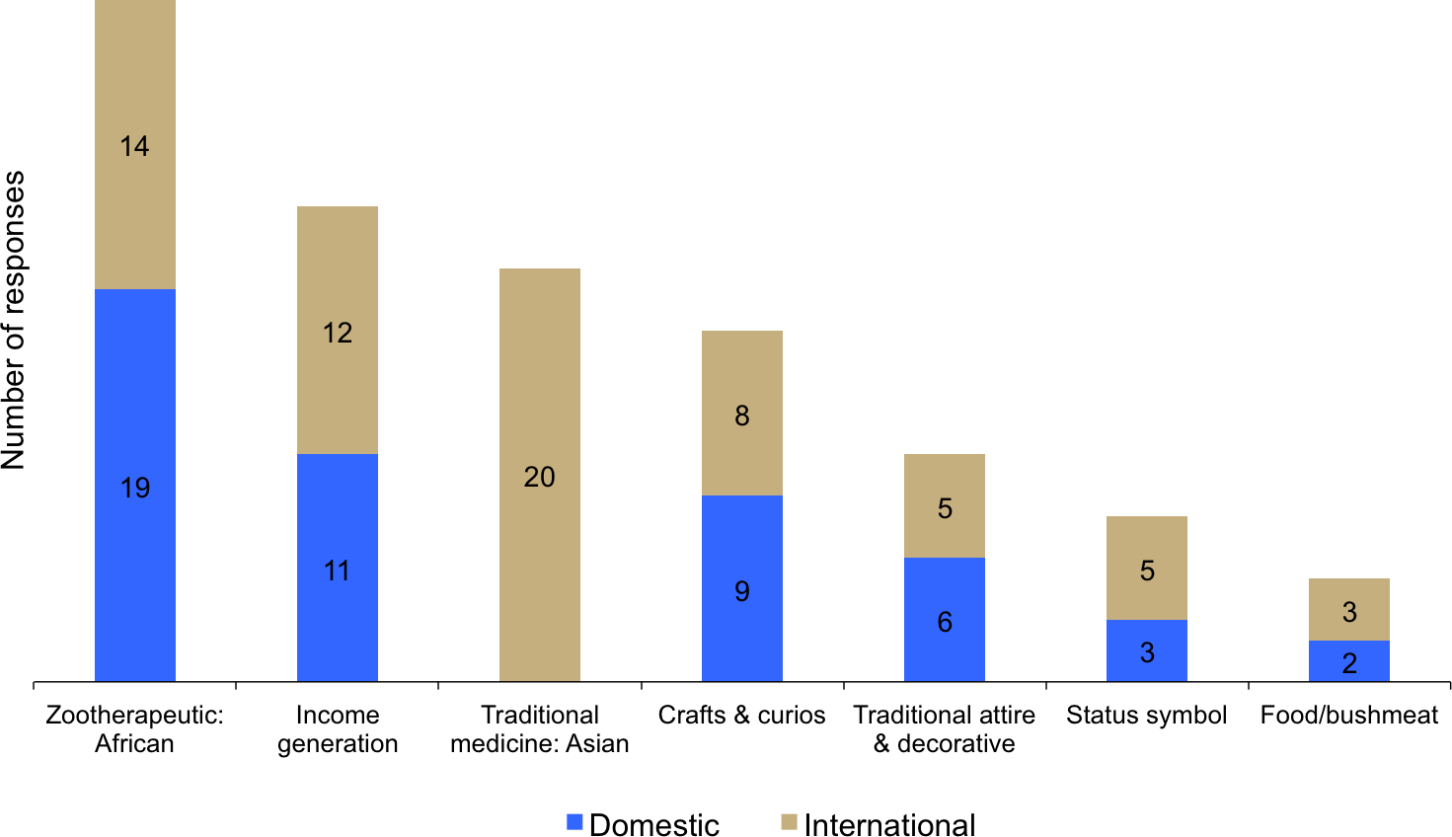
The perceived reasons why lion bones are used and traded, domestically and internationally, within and between, African countries and East-Southeast Asia. Answers correspond to Questions 12 and 13.

Like the trade in body parts, African zootherapeutic practices and also Traditional Asian Medicine are perceived by respondents to be the main purposes for which lion bones are used and traded, domestically and internationally, within and between, African countries and Asia (Fig 8). Other purposes for bones include income generation and crafts/curios (purportedly for the tourist market) (Fig 8). While these are the primary uses for bones, African zootherapeutic practitioners and consumers mainly use the fat, claws, skin and teeth for healing (see later). The physical strength displayed by lions is often believed to be a trait that is transferable to humans to impart comparable strength to overcome adversity [140], and the bones (especially carpals and tarsals) are routinely used as divination instruments by ‘bone-throwing’ traditional healers [5].

In response to the origin of lion bones, the general perception was that they were mainly obtained from wild lions that are poached, trophy hunted, and killed in retaliatory reactions to human-lion conflicts (so-called ‘problem lions’) (Fig 6A), especially in Southern, West and East Africa. In South Africa, however, bones were known to originate mostly from captive lions (Fig 6B). A respondent from Botswana alleged that wild lion carcasses from there were being smuggled into South Africa. And, a respondent from Zimbabwe wrote that there were *“circulating rumours about several lion breeders in Zimbabwe trading lion parts (whole carcasses)”* and that these parts were being *“moved to South Africa”*. Since there are no corresponding CITES records of this cross-border trade, these anecdotal reports may flag the presence of illegal lion trade between South Africa and its neighbours.

In 2014/15, the domestic market for lion bones within range states was perceived by respondents to be having little or low impact overall on wild lion populations. However, the overall response rate to the impacts being unknown was 35% (Fig 7), so this uncertainty merits vigilance, especially with the apparent rise in instances of lions being poached and poisoned since the survey was conducted and speculation that this is partly driven by domestic or international trade. Some of these instances have the hallmarks of poaching for the traditional medicine and/or craft/curio trade, and we therefore caution against uncritically attributing every event to the Asian lion bone trade. Respondents felt that the highest impacts of the domestic market for bones were to wild populations in Southern and West Africa (S4 Fig), and that the international trade was having a greater impact on wild lion populations across the continent (Fig 7, S5 Fig) and probably somewhere in each range state. Hence, the intra-African demand (i.e. between range states) for lion products is a likely to be an anthropogenic risk factor in population decline.

There was a low response rate to requests for information on the domestic and international supply chain for lion bones (except for South Africa, which we have written about in [5,8]) (Question 19, see S2 Table, where the ‘supply chain’ was defined as *‘e*.*g*. *suppliers*, *middlemen*, *exporters*, *importers*, *how bones leave the country*, *etc’*). The supply chain in most range states is unclear and typically appears to be more a matter of suspicion and speculation that requires further verification. Respondents *“had heard that…”* certain activities were occurring.

The acquisition of bones typically arises through three routes: (i) opportunistic collection (e.g. lions caught in bushmeat snares), (ii) deliberate acquisition (e.g. poisoning, poaching, commercial and subsistence hunting), and (iii) a by-product of trophy hunting (captive or wild), all of which, depending on the consumer/customer, may or may not involve Asian nationals living in Africa. The supply chain may also involve professional hunters selling bones illegally to traders, and also “*lion parts being concealed within loads of charcoal and cotton*” (Resp’t #4, Zambia). A few respondents mentioned carbofuran poison being used at waterholes in parks and concessions in Mozambique and Zimbabwe to kill lions, and resulting in the carcasses being processed to convert the bones to *“finer materials”* for export to Laos and Vietnam via South Africa, or for the parts to enter the local markets via traditional healers. In Zambia, a respondent was aware of at least two instances where Congolese poachers were caught with lion bones, and another instance where Zambian nationals destined for Lusaka were caught with ‘problem lion’ skulls they had allegedly obtained from ZAWA (Zambian Wildlife Authority) officials in Kafue National Park. Mention was also made of the suspect activities of some Chinese workers living in Africa, who were allegedly soliciting bones and carcasses from rural villagers, bribeable government officials and park wardens, and/or trophy hunting operators. It seems clear that the tentacles of the lion trade source and supply chain are persistently probing within lion range states for new purveyors, routes and exploitable weak links.

The overall low response rate to specific questions on the lion bone trade (except for South Africa) appears to indicate a deficiency of substantiated evidence within Africa, but cannot be taken as evidence for the absence of trade. The trade in lion bones and body parts for multiple purposes is widespread, illegal (except where people acquire permits) and somewhat of a ‘hidden economy’ (similar to the description of the South African traditional medicine trade by [150]). Presently the best available information on the trade in lion bones and other body parts in Africa is derived from the legal CITES-monitored trade, but this is restricted to information on self-reported, legal, international trade only. Hence substantiated evidence for illegal trade typically (and importantly) relies on sporadic informal reports shared between conservation agencies and concerned individuals, or reports of confiscations at international ports of exit.

## Sub-regional summaries

The overall perceptions of the respondents to trade, purpose for parts, sources of derivatives and the impact of trade, are compared sub-regionally in a table that summarises the highest responses per question (Table 3). An expanded account of the sub-regional results (which includes all the anecdotes from the comment section of most questions) is available in S1 Doc.

The results from the pan-African survey, as shown for each question in Table 3, can be summarised sub-regionally and overall as follows: (1) *Domestic use/trade awareness*: informants perceived claws, skin, teeth and fat to be used/traded the most; (ii) *International trade awareness*: informants perceived bones, claws and teeth to be traded the most, but there were subregional differences (e.g. urine in Central Africa); (iii) *Reasons for domestic trade*: informants mostly perceived African zootherapeutic practices, traditional attire and income generation to be the main purposes of domestic trade (except for Southern Africa, where it was crafts/curios); (iv) *Reasons for international trade*: informants mostly perceived zootherapeutic practices (African and Asian) and income generation to be the reasons; (v) *Sources for body parts (excluding bones)*: body parts were mostly thought to be sourced from ‘wild poached’ and ‘wild problem’ lions, except for South Africa where the main sources cited were captive; (vi) *Sources for bones*: bones were mostly thought to be sourced from ‘wild poached’ and ‘wild trophy hunted’ lions, except for South Africa where the main sources cited were captive; (vii) *Impact of body parts trade*, *domestic and international*: within sub-regions, the domestic trade was believed mostly to be having a ‘low’ and/or ‘unknown’ impact on wild populations (with medium impact in East Africa), and the results overall were similar. Importantly for the international trade in body parts, there was a high rate of nominations for ‘unknown’ impact. Perceptions for impacts being ‘high’ were greater for the body parts trade than for the bone trade; (viii) *Impact of bone trade*, *domestic and international*: informant opinion was generally that the impact on wild populations was ‘low’ and/or ‘unknown’ across all sub-regions. Although there is only fragmentary literature with which to compare the generality of these conclusions, we have found none that specifically contradicts them. As in each aspect of this report, further research seems to be merited.

**Fig 9:**
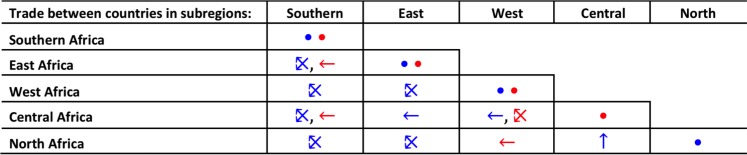
Summation of documented legal and/or illegal intra-African sub-regional trade in lion derivatives. Individual, country-specific records are in S3 Table. **Blue** and **red** symbols indicate records of *legal* (source: CITES Trade Database) and *illegal* (source: questionnaire and literature surveys, personal communications) trade respectively. Arrows point towards the recipient sub-region from the source sub-region; the ⤪ symbol indicates records of bi-directional source-recipient trade. The solid circles (**•**) indicate trade records within one sub-region.

Documented and anecdotal reports of legal (i.e. with CITES permits) and/or illegal trade between African countries are consolidated and detailed in S3 Table to demonstrate where trade links have been recorded, and this is summarised for sub-regional trade in Fig 9. To our knowledge, trade between Central African countries has only been recorded as illegal, as has trade between countries in the sub-regions of West and North Africa (Fig 9; S3 Table). However, trade between countries in North and East Africa tended to be reported as legal (CITES Trade database), but illegal sub-regional trade was more likely to be reported with Southern and especially West and Central Africa (Fig 9; S3 Table). Intra-regional trade between West African countries, especially those with small and diminishing lion populations, was being red-flagged by respondents. That mostly legal trade has been documented for North and East Africa does not mean that illegal trade between them and other countries in other sub-regions does not occur–rather, no documented evidence came to our attention in the period that this study was being conducted.

## Priority range states nominated for further investigation

Seventeen African range states were nominated at least once as priorities for further investigations into the lion derivatives trade and the impacts thereof (Fig 2; S6 Fig) (11 countries received ≥2 votes). While Southern and South Africa had the most nominations, 10 other countries were also proposed: Zimbabwe, Mozambique, Zambia and Botswana in Southern Africa; Tanzania and Kenya in East Africa; Nigeria, Benin and Burkina Faso in West Africa; and, Cameroon in Central Africa (Fig 2).

Respondents qualified their nominations by saying that priority range states were those that: (i) allowed *trophy hunting*, as it created opportunities to participate in the lion body parts trade and the bone trade to East-Southeast Asia; (ii) have some *captive breeding*, as this also created a potential supply of bones for Asian markets; (iii) are ‘hotspots’ for *poaching*, because this activity could supply derivatives to the local markets and the tourist trade, and will probably supply parts for international trade; (iv) have an active trade in *curios and souvenirs* for visiting tourists (especially in East and Southern Africa); (v) have an active *domestic trade* in lion products (e.g. for traditional practices, especially in West Africa); (vi) tend to have *larger lion populations*, as this could entice more illegal trade and consumptive utilisation at a larger scale (however, one could argue that countries with smaller lion populations have a proportionally higher risk); (vii) have a *history of wildlife trade* (legal and illegal), as the trade networks are likely to be established and operational; and, (viii) are *transit routes for illegal trade* (e.g. Tanzania, Kenya, Uganda).

The following answer from Resp’t #39 about research priorities encapsulates the sentiments of most other survey participants: “…*In terms of the legal bone trade from South Africa*, *this has spawned much ‘interest’ in similar trade from a diversity of countries where lions are trophy hunted*. *Most specifically Tanzania*, *Namibia*, *and Zimbabwe*. *The bone trade provides additional income for the hunting operators…The tourist trade in lion derivatives is growing in terms of items made of skin*, *teeth and claws sold openly on local markets in Zimbabwe*, *Zambia*, *Tanzania and Kenya as ‘souvenirs’ and ‘curios’*. *Such products are largely derived from poached lions*, *those killed in retribution for stock raiding*, *and even lion killed in road accidents…There is also a very significant internal market for lion products in all African range states*. *Local markets in western Africa and Zambia*, *Zimbabwe*, *Kenya*, *Mozambique and Tanzania all offer a diversity of lion products to local buyers–so not part of international trade*”.

## Literature summation

**Fig 10:**
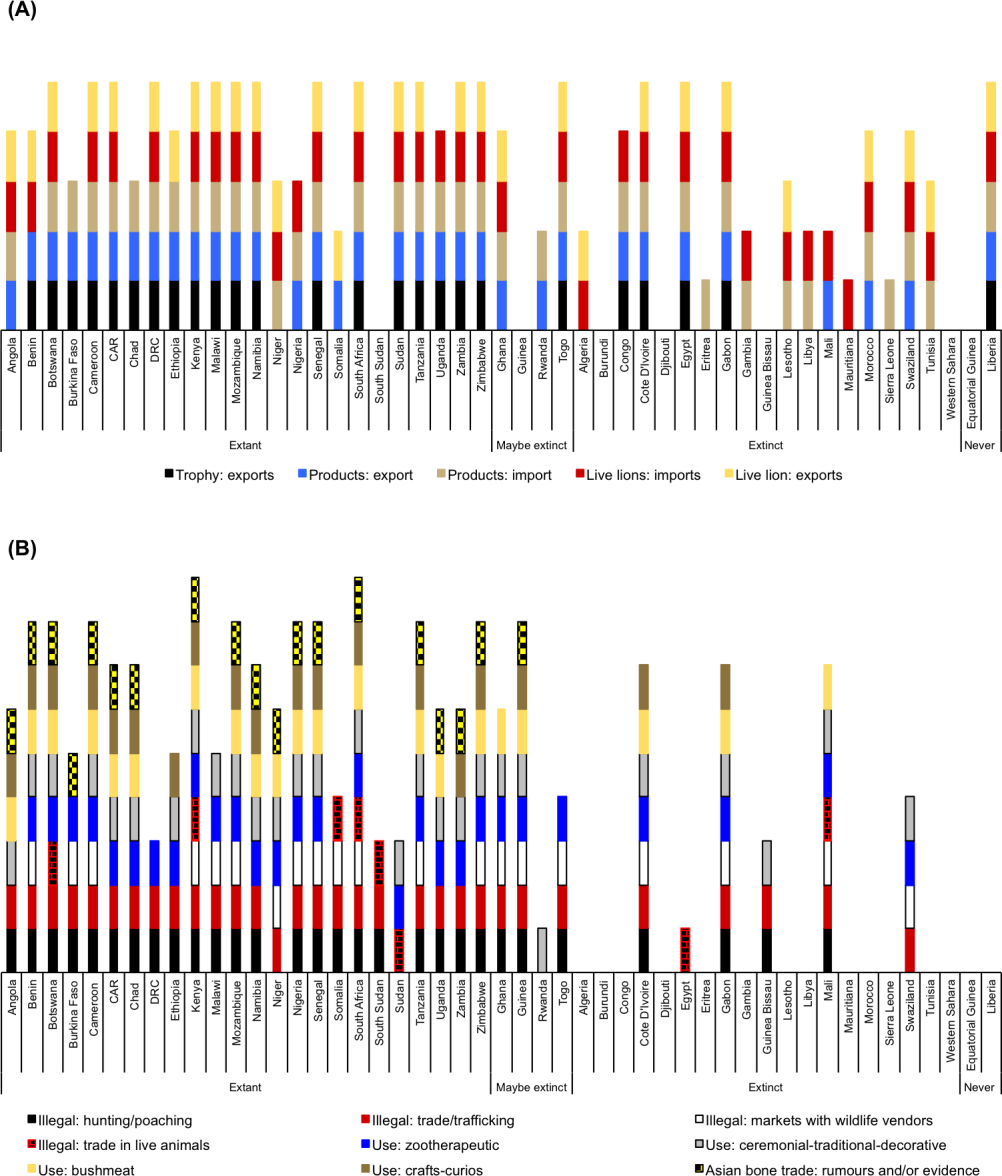
Records for categorised legal and illegal activities documented at least once within African countries. (A) Records of CITES permits having been issued for ≥1 year to export or import live lions and/or products; (B) records of illegal activities concerning lion products, and uses for lions parts, having occurred or been documented in the literature and/or the questionnaire survey. Absence of records does not infer that certain illegal activities have not occurred, nor that lion parts are not used or traded, in a country; record gaps indicate either that information for the activity was not located (despite occurring) and/or that these activities do not take place in a country. Numerical information for Fig 10A is in S1 Table.

**Fig 11:**
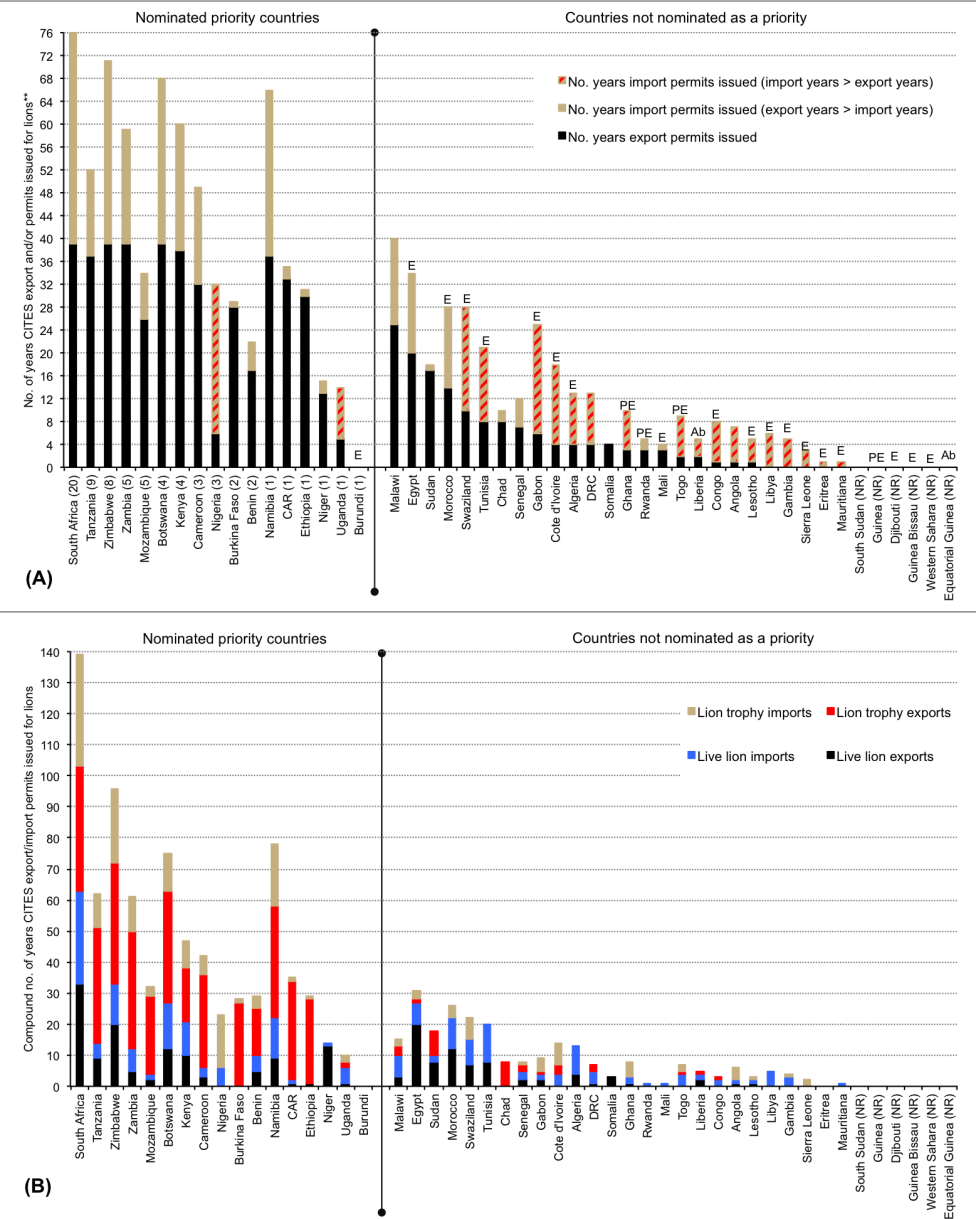
Relative number of years that CITES export and/or import permits were issued for lions* in African countries nominated by survey respondents as priorities for future trade research, compared to non-nominated African countries (*live and/or products). **(A) Number of years permits were issued for all lions (live and/or products), and showing countries where the number of years that import permits were issued was greater than the number of years that export permits were issued. (B) Number of years that permits were issued to export/import live lions and/or trophies.** Countries are arranged in the same order in A and B, with priority countries (LHS of the vertical dividing line) in descending order of the number of nominations (number in brackets in A). Non-nominated countries are listed (in A) in descending order of the number of years export permits were issued, followed by import permits issued. NR = no CITES records for the country for lion trade; Ab = countries that never had lion population; E = lion populations extirpated; PE = lion populations possibly extirpated. CITES record keeping commenced in 1977 and is correct to 2015. (Raw data and the total number of years that CITES export/import permits were issued are in S1 Table). Data are not inferred to be a proxy for actual volumes.

**Fig 12:**
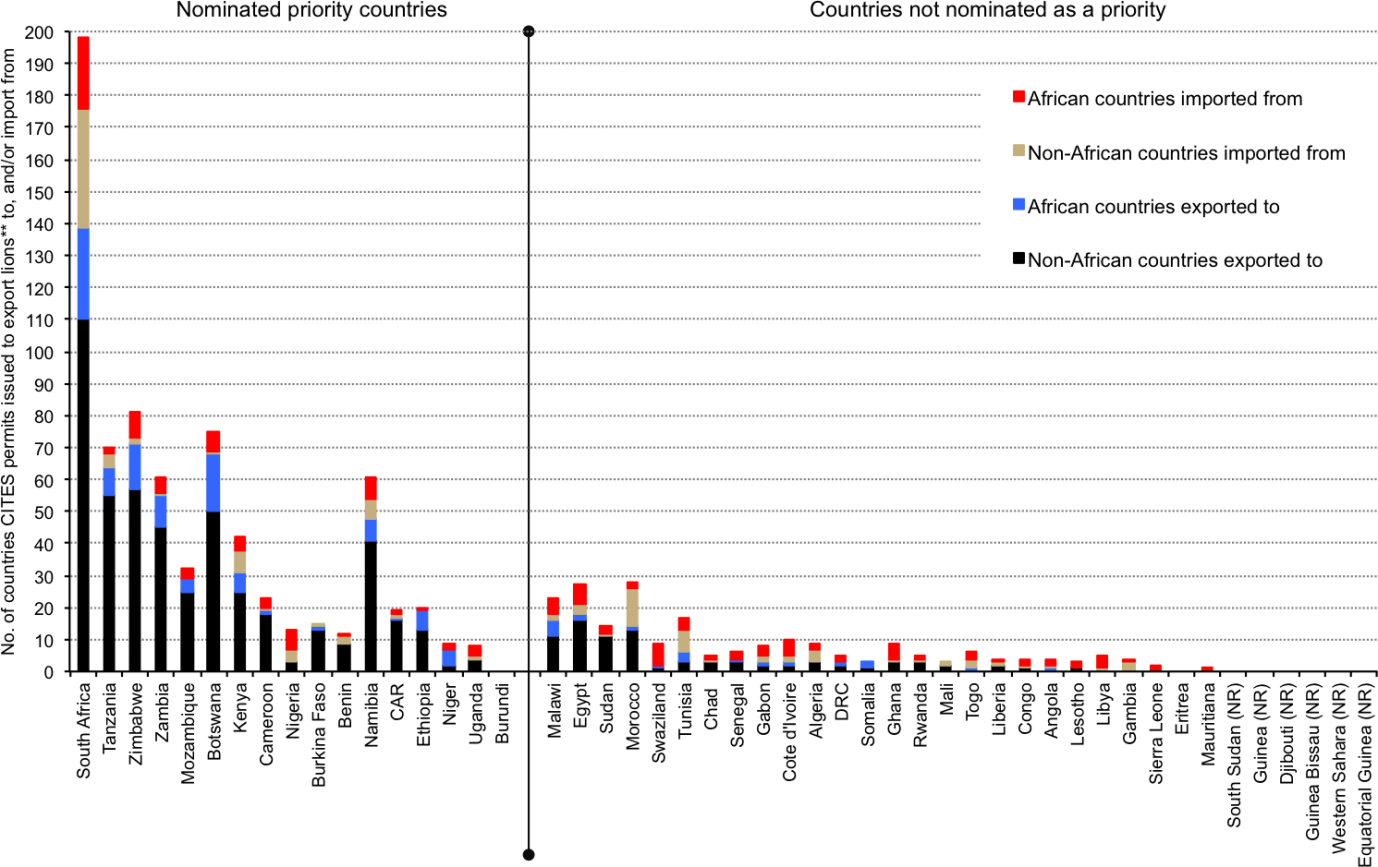
Number of countries (African and non-African) that priority-nominated and non-nominated African countries exported lion* to, or imported them from. Countries arranged in the same order as Fig 11. (*live and/or products).

A summation of the literature, presented as a categorical précis of African trade, use and lion population statuses in S1 Table and Fig 10 (also Figs 11 & 12 later), was drawn from references [9–147], the CITES database (for the number of years that CITES permits have been issued to export or import selected products and/or live lions per country), and pertinent survey results (*viz*. the categories of use). However, we abstained from including a lengthier analysis of legal lion trade extracted from the CITES database (e.g. quantities) in order to focus on the results of the pan-African survey. Furthermore, CITES published a comprehensive report on the status of African lions in 2014 that included an analysis of the scale and proportion of legal trade [14].

Tanzania has the largest wild lion population (>15000 individuals), followed by Mozambique and South Africa (>2000 wild lions each); six countries have populations of 1000–2000 individuals each (Angola, Botswana, Central African Republic or CAR, Ethiopia, Kenya, Zambia) (S1 Table). Lions have been extirpated in 18 countries (including 10 where extinction occurred within the last 20 years), in which legal lion trade periodically persists (mainly imports of live animals, skins and trophies) [CITES Trade Database]. Of the former range states, CITES reports indicate that countries in North Africa (especially Egypt) mostly trade in live lions (Fig 10A and Fig 11B later), countries in Central Africa mostly import skins (Gabon, Congo), and West African countries import a range of lion products. Egypt is reported to be a regular exporter of captive-origin live lions, especially to the Middle East [CITES Trade database]. For countries with small and/or extirpated lion populations there was, however, no noticeable switch from being exporters of lion products to being importers as a means to predict population decline; such countries have, since CITES record keeping began in 1977, typically reported lower levels of trade and more imports than exports of lions (live and/or products) (see Fig 11 later).

Between 1977 and 2014, legal trade in lion body parts was reported by CITES in 23 of the 24 current lion range states (except South Sudan), three of the four countries with possibly extinct populations (except Guinea), and 15 of the 19 countries where lions have been extirpated (except Burundi, Djibouti, Guinea Bissau, Western Sahara) (Fig 10A; S1 Table). Furthermore, annual reports for nine range states shows >30 years of mostly continuous legal international trade (Botswana, Cameroon, CAR, Kenya, Namibia, South Africa, Tanzania, Zambia, Zimbabwe) (mainly hunting trophies) (S1 Table). There were 11706 entries for lions on the CITES Trade Database where African countries were listed as the origin and/or exporter/importer (82% of all lion records for 38 years). The proportion of entries for specific trade terms (*not quantities*) are: 42% trophies, 36% skins or skin pieces, 12% skulls, 9% live lions, and 4% for bodies, bones and skeletons combined. Also listed were 17 entries for ‘specimens’ traded under the ‘medical’ purpose code, including 202 wild origin lion ‘specimens’ destined for China from South Africa and Botswana.

The Sudanese case is worth noting because no CITES trade records for lions from Sudan have been reported since the South Sudanese secession in 2011. Furthermore, South Sudan has not acceded to CITES yet and therefore trade is not being monitored. Given that the bulk of the former Sudanese lion population (pre-2011) is in what is now South Sudan, it is likely that most trade stemmed from lions from this population.

There are published accounts of lion products being sold in wildlife markets in 13 range states (Benin, Burkina Faso, Cameroon, Kenya, Malawi, Mozambique, Niger, Nigeria, Senegal, Somalia, South Africa, Tanzania, Zimbabwe), but also markets in seven countries with possibly extinct and extinct populations (Ghana, Guinea, Togo, Cote d’Ivoire, Gabon, Mali, Swaziland) (Fig 10A; S1 Table). The questionnaire survey did not reveal new countries. All countries with informal markets selling lion parts are also documented to have consumers using lions for zootherapeutic purposes, however use of lions for this purpose also occurs in most range states where evidence for trade in informal markets has not yet been formally published and/or identified (*viz*. Angola, Botswana, CAR, Chad, DRC, Ethiopia, Namibia, Sudan, Uganda, Zambia) (Fig 10A; S1 Table); the online survey provided new information for countries where published information had not been found. Hence, zootherapeutic practices emerge as important stimuli of the local and intra-African demand for lion body parts across the continent. Ceremonial and decorative uses of lion parts are also prevalent, and lion bushmeat consumption was documented in the literature for eight countries (five in West Africa); however, additional information received from the survey respondents suggests that bushmeat consumption occurs in at least 21 countries (Angola, Benin, Botswana, Cameroon, CAR, Chad, Kenya, Mozambique, Namibia, Niger, Nigeria, Senegal, South Africa, Tanzania, Uganda, Zimbabwe, Ghana, Guinea, Cote d’Ivoire, Gabon, Mali) (Fig 10A). The illegal trade in live animals has only been reported in six countries, namely between Botswana and South Africa, and also in Ethiopia, Somalia, Sudan and South Sudan (e.g. lion cub smuggling in Somalia [47]) (Fig 10A).

While reports of illegal lion hunting and trade have been published for most range states, there was a paucity of accessible corresponding information for Angola, the DRC, Ethiopia, Niger, South Sudan and Sudan (Fig 10A; S1 & S3 Tables); there is a similar absence of data, but for lion population sizes, in Angola, CAR, Ethiopia, Somalia and South Sudan [2]. The bulk of the original Sudanese lion population is inferred to be in South Sudan following the secession of the latter from Sudan in 2011. In the published accounts, illegal trade in lion products had been described as ‘rare’ or ‘insignificant’ in Namibia, Tanzania, Zambia and Zimbabwe–but some participants in the survey indicated that there has been a recent escalation in illegal activities in these countries that is a cause for concern (e.g. trade in lion claws in northern Namibia is a huge worry) (S1 Table). In some cases, ‘problem lion hunts’ were alleged to be an excuse for poaching. West Africa, however, has been described as a major hub for illegal intra-African trade. Most notably Benin, Burkina Faso, Niger and Nigeria are reported to be sources of lion products, whereas Gabon, Senegal and Cote d’Ivoire are the recipients/importers. In Central Africa, Cameroon is a noted supplier of lion parts to Nigeria and Chad (S3 Table).

Illegal lion hunting and poaching occurs across the continent, including countries in which lion hunting is legal (Fig 10A). Range states allowing hunting have fluctuated over the years, with some imposing short-term moratoriums banning hunting. Since mid-2015, re-evaluations of national hunting policies have taken place and, as of June 2016, lion hunting was permitted in 17 range states and was only prohibited in six (namely Angola, Botswana, Kenya, Malawi, Niger, and Nigeria) (S1 Table). Countries in East Africa (except Tanzania) generally only allow problem lions to be hunted, including Kenya where trophy hunting has been banned since 1977.

### African healing practices and the lion trade

Lions are symbolically powerful animals and use of their parts has recurrently represented control over the supernatural [151]. Understanding why lions and/or their specific parts/derivatives are used/traded to the point where supply chains from the source to the consumer are established is key to addressing regional drivers of trade–for example, the dynamics of the lion urine trade in Niger, allegedly harvested from the cages of several lions in a museum in Niamey, and supplied to customers from Nigeria, Senegal, Cote d’Ivoire and Togo who use it to treat asthma [134]. There are also important socio-economic drivers behind resource use that are not addressed here.

The use of lions for zootherapeutic practices has not been widely or thoroughly documented across the continent, and the literature offers scope for a separate review. And, as elucidated from our questionnaire survey, uses of lions for these purposes was perceived and revealed to be an important factor in the domestic and international trade in parts across the continent (Fig 5). Zootherapeutic uses incorporate both medical conditions and symbolic social or cultural issues [146]; these uses are largely allied with the ‘Doctrine of Signatures’, where purposes for certain body parts can be predicted from the characters that the form suggests [146]. ‘Medically’, for example, lion lungs have been used to treat whooping cough, and the bones to treat rheumatism and fractures [33,94,121]. ‘Pseudo-medically’, the use of the throat (which may include the voice box) is alleged to increase the sound of the patient’s voice. Symbolically, however, bones and other body parts are alleged to imbue personal and spiritual strength, instil fear, and enable one to become intimidating [33,94,99,119,146]. ‘Strength’ was determined to be the main use for vertebrates in a study in South Africa by [146]; appropriately, they asked whether pervasive feelings of powerlessness and insecurity within these consumers was factor in their use of symbolically strong animals to overcome weakness.

Across the continent, however, lion fat seems to be a general panacea for most ailments–probably because it bears no resemblance to a character for which one can attribute a specific use, and it can be applied with ease to the body (no ingestion required). Fat also appears from the literature to be one of the most frequently used parts, albeit one that is most likely to be substituted with fakes (even though consumers acquire it from suppliers in the belief that it is genuine) [151].

Consensus on what specific lion body parts are used for, and/or what ailments are treated with the parts, is variable. For example, an investigation on uses for lion parts in Nigeria [33], reported 163 mentions for fat that were classified into four broad use types. The degree of informant consensus (ICF, calculated here using the formula in [146]) for what the fat was used for was 98%—i.e. a low degree of use heterogeneity. We similarly calculated ICF values for lion skin, bones and teeth for the Nigerian study, revealing consensus values of 96%, 94% and 87% for part use respectively (incidentally, fat, skin, bones and teeth are the parts that received the most citations for use overall). However, in a snapshot survey of 30 studies listed in 15 publications where lion part use was mentioned for 12 countries across Southern, West, East and Central Africa [33,35,42,58,76,94,99,104,106,119,121,134,146,151,152], consensus on fat use was only 11% (17 different uses in nine countries for purposes such as allergies, polio, depressed fontanels, dignity and lucky charms) and consensus on bone use was 30% (eight uses between four countries, including rheumatism and fractures).

There are thus regional cultural complexities and preferences in the zootherapeutic use of lions across the continent. One such matter that could shed light on trends in lion poaching in southern Africa, for example, has not been described before to our knowledge. A division within southern African spiritual traditions for training as the type of traditional healer called an *izangoma* (or, diviner) involves initiation through the *ukuthwasa* (‘*thwasa*’) processes. This training is further differentiated into traditions of practice involving variations in sacred spirits, sites, rites and animals. One of these traditions of practice is called ‘*umndawu*’ (the isiZulu spelling, with variations depending on the cultural region of practice e.g. *ndau*, meaning lion, in Tshivenda). Lions are sacred animals to these healers, and *umndawu* spirits are “*believed to have the strength*, *power*, *mastery and other attributes of the lion*” [153; N. Mbongwa, pers. comm., July 2017).

Healers called to *thwasa* with *umndawu* are required to have no more than two lion bones in their divination sets (carpels, tarsals and/or patella); bones should preferably be obtained from wild lions from Mozambique, but no other part of the lion can be used (however, healers who are trained within other traditions are allowed to incorporate lion derivatives such as fat and skin into their healing practices) [N. Mbongwa, pers. comm., July 2017]. The *umndawu* tradition originated along the east coast of Africa and spread to eastern Zimbabwe and South Africa [153], and is particularly prevalent in Mozambique south of the Zambezi Valley among Xitsonga (‘Shangaan’) communities. *Umndawu*-trained healers are regionally prevalent in South Africa (e.g. estimated 60% of healers in Johannesburg; 70% in Limpopo and Mpumalanga Provinces combined; 35% in KwaZulu-Natal Province, but more predominant in certain northern municipalities such as Ingwavuma and Umhlabuyalingana near Mozambique [N. Mbongwa, pers. comm., July 2017]) and neighbouring countries.

We suspect this to be another plausible factor in lion poaching in the sub-region (along with other stimuli, such as the Asian market for claws, teeth and bones), which is exacerbated by the high prices charged by traders to healers for lion parts (e.g. R500 (US$38) per carpel or tarsal, and R750 (US$58) per patella, in urban areas [N. Mbongwa, pers. comm., July 2017]). Historically, healers would, or should, have personally sourced bones from naturally wounded or deceased lions where there was no human intervention in their death [N. Mbongwa, pers. comm., July 2017]. However, multifaceted dynamics relating to socio-economics, urbanisation and migration, have created gaps in the resource supply chain for hunters and ‘muti’ traders to become market middlemen and to procure lion body parts for consumers. And, since people purchasing these parts have no knowledge of their actual origin, they can be persuaded that they are purchasing parts obtained from wild, Mozambique-origin lions that died naturally–even if the parts entering the supply chain were from captive-bred and/or trophy hunted and/or wild South African lions (as has been reported), or possibly even lion bone fakes.

Overall, the questionnaire survey elicited important latent information (mainly based on personal observations) from the respondents concerning the perceived purposes of lions and their parts, and the relative prominence thereof, e.g. the tourist trade as a stimulus for jewellery made from teeth and claws. But, these perceptions (albeit based on informed opinion) require further corroborative research to fully unravel the demand for different lion parts. With the exception of investigations into the lion bone trade to East-Southeast Asia [5,7,8], the literature documenting uses for lions tends to be fragmented and might fruitfully be the topic of a further review.

## Discussion

There were anecdotes, allegations and evidence and a gradient of opinions expressed relating to the utilisation of, and trade in lions (live, body parts and derivatives) across Africa. This may indicate that threats to wild lion populations vary across this continuum of continental trade, as do the impacts of legal and illegal activities that support the acquisition of body parts and derivatives. Judging from our survey and the CITES trade reports, there are established trade flows of lion body parts within Africa, and importation of parts from countries with no lion populations. The socio-ecological element to the trade in lion parts, however, presents an added conservation challenge that has the characteristics of a ‘wicked problem’ [154,155]]: namely, one that is socially complex, possess numerous cause-and-effect relationships and problems that are nonlinear and hard to isolate, and which similarly requires solutions “*that are not obvious and require collaboration among stakeholders to determine appropriate actions*” [155,154].

In terms of sub-regional differences in domestic use and international trade, patterns emerged that reflected differences in the extent of informal versus more formal and/or commercial markets and trade. Differences in corresponding consumptive and non-consumptive activities and trade will have more marked, negative impacts for countries with smaller lion populations and/or relatively larger demands for lion products. In Central and West Africa, the diverse local market for lion body parts was considered to be the biggest threat to vulnerable lion populations. In East and Southern Africa, however, there was thought to be a four-fold greater threat from discrete sectors, *viz*. trophy hunting, poaching for parts for the tourist trade (skin, teeth and claws), the international lion bone trade, and body part use for ostensibly traditional purposes.

When integrating the results from the pan-African survey with the literature and CITES trade records (S1 Table; period 1977–2015), the following characteristics emerge for 17 countries nominated as priorities (partly summarised in Figs 11 and 12, LHS of dividing line): (i) 16 have extant lion populations; (ii) there is regular reported annual legal trade in lions (products and/or live), and the average number of years for which CITES permits (export and/or import) were issued is 31±9 years (range 14 to 39 years; S1 Table); (iii) CITES permits to export lion products and/or live lions were issued for more years than import permits were issued–but with the exception of Nigeria and Uganda; (iv) the average number of export destinations is 37±36 countries (of which 9±8 export destination countries are African) (compared to 5±5 destination countries from non-priority range states, 2±1 of which are African) (S1 Table); (v) the average number of countries they import from is 9±14, of which 5±5 countries are African (compared to non-priority range-states importing from 5±3 countries, 3±2 of which are African); (vi) trophy hunting tends to be commonplace (especially in Southern and East Africa) (CITES export permits for trophies were issued for an average of 25±14 years per country, or 75% of a countrys’ total years that CITES permits were issued to export lion) (S1 Table); and, (vii) illegal trade has been recorded for more years in these countries than for non-priority countries (inferred from CITES seizures and confiscation records; mean 5±6 years of records, ranging from 0 to 24 years for priority countries). Burundi is the anomaly in the nominated priority group as its lion population is extinct, there are no CITES trade records, and there are no records in the literature for trade and utilisation (S1 Table). Since the respondent that named it (Resp’t #17) stated only ‘trade’ as the motivation, we assume that the absence of information led to its selection; therefore, we do not consider Burundi to be a priority for East Africa.

In contrast to the nominated priority lion range states, the countries not nominated have some of the following characteristics (Figs 11 and 12, RHS of the vertical dividing line): (i) lions have mostly been extirpated, and/or population numbers are relatively low (except for Angola and Somalia and South Sudan; S1 Table); (ii) reported annual trade in live lions and parts is irregular (average 11±8 years that CITES export and/or import permits were issued; range 1 to 28 years); (iii) CITES import permits were issued for relatively more years than export permits; (iv) number of export destinations is 5±5 countries (2±1 of which were African); (v) CITES export permits for trophies are relatively rare (CITES export permits were issued for an average of 1±2 years for all the trade years, or 18% of a countrys’ total years that CITES permits were issued to export lion); and, (v) annual records of illegal trade also rare (<1 year out of all the years trading).

The countries and sub-regional priorities nominated by respondents in relation to local use and international trade were motivated mainly for the following reasons: (i) in Southern Africa, the extensive trophy hunting and captive breeding industries makes large quantities of lion bones and other products available that are distributed within a legal/illegal domestic/international supply chain (e.g. as evidenced by lion skins appearing in South African traditional medicine markets alleged to have been supplied by a captive breeding facility, pers. obs.); (ii) in East Africa it was frequently alleged that lions are being poached to supply body parts for the tourist trade (such as claws, teeth and skins) (tourists are ostensibly led to believe these products are obtained from natural mortalities), and a smaller domestic market; (iii) in West Africa, the trade is perceived mainly to be for local consumption (with notable cross-border trade) that warrants further scrutiny; (iv) little has been documented on trade in Central Africa, thus making it a candidate for investigation. Hence, the purpose of any future field-based studies would be to detail local and international supply chains from source to destination, the prevalence of illegal activities, and the impact of all these utilisation- and trade-related activities on wild lions across the range states.

## Conclusion

Trade in African lion body parts is widespread and ranges from products for local consumption, to sport hunting trophies, curios sold in the tourist trade and, since 2008, to bones sold in East and Southeast Asia [5–8]. Furthermore, there are sub-regional differences in consumptive use, drivers and stimuli of trade, and access to lions that impact wild lion populations in different ways and to different degrees. In general, the respondents acknowledged and/or suspected that trade in lion body parts is an established and growing concern–but the scale and imminent impact was largely unknown and not fully estimated or assessed in terms of the immediate risks to wild lion populations across the continent.

This survey and recent reports of lion poisoning and poaching in Mozambique, Zimbabwe and South Africa, and sporadic poaching events in Uganda and Tanzania (not detailed in this paper), is signalling an escalating and worrisome trend that the trade of lion derivatives is increasingly becoming a more substantial threat to certain national populations. There is therefore a necessity for information sharing and consolidation, trade monitoring, and risk assessment in all countries. But while this survey identified some trade hotspots and the motives for utilisation and demand, it could not identify all the supply chain participants and their motives. As one respondent pertinently asked: “*who is ordering and buying the stuff*, *and why*?”

The unanswered question from the perspective of our study is, *‘insofar as it is detrimental to lion conservation*, *what could be done to mitigate the impacts of trade’*? Until most drivers of demand and remedies have been identified, the most obvious solution lays in better global, regional and national coordination, management and enforcement as envisaged by Doc CoP17 Com. I. 29 [156] arising out of CITES CoP17, and in the implementation of CBD CoP11 Dec. XI/25 on sustainable wildlife management and use of biodiversity [157]]. However, to achieve these goals it is necessary to understand the drivers of supply: our survey and the prioritisation of countries for immediate attention is a first step towards this.

## Supporting information

S1 Table Summary of the information on lion trade, utilisation, population size and illegal activities, obtained from the literature and questionnaire surveys for current and former lion range states in Africa. (PDF)

S2 Table Survey questionnaire on the trade and use in lion body parts distributed to respondents and administered using SurveyMonkey® (PDF)

S3 Table Records of intra-African trade in lion body parts, with symbols indicating whether trade between two African countries has been recorded as legal only, illegal only, or both legal and illegal. (HTM)

S1 Doc Sub-regional summaries for Southern, East, West, Central and South Africa (13 pages) (PDF)

S1 Fig Sub-regions in which respondents worked, and for which they had information on lions (answers include multiple responses). (PDF)

S2 Fig Respondents’ area of expertise and/or occupation and/or type of employment (PDF)

S3 Fig Number of years respondents had been involved in lion conservation or allied wildlife matters (PDF)

S4 Fig The perceived impact of the domestic trade in lion (A) body parts (sub-regional), (B) bones (sub-regional), (C) body parts (per nominated range state), (D) bones (per nominated range state).
(PDF)

S5 Fig The perceived impact of the international trade in lion (A) body parts (sub-regional), (B) bones (sub-regional), (C) body parts (per nominated range state), (D) bones (per nominated range state).
(PDF)

S6 Fig Informant opinion on which African Lion range states are the most important in which to conduct studies *vis-à-vis* the trade in body parts. (PDF)
