# Supplementary material for: Questionnaire survey of the pan-African trade in lion body parts
Source: PLoS One. 2017 Oct 26;12(10):e0187060. doi: 10.1371/journal.pone.0187060 (PMC5658145; doi:10.1371/journal.pone.0187060)
Supplement: S1 Table — (PDF) [file pone.0187060.s001.pdf]

S1 Table: Summary of the information on lion trade, utilisation, population size and illegal activities, obtained from the literature and questionnaire surveys for current and former lion range states in Africa. (Williams, Loveridge, Newton, Macdonald (2017); PLoS ONE

**Country** (Countries in **bold** were nominated as priorities; Countries in *italics* on the LHS of column A were not mentioned by respondents during the survey; Countries not in bold and also on the RHS of column A were mentioned during the survey but were not nominated as priorities)	**Country Code**	**Region** (South, East, West, Central, North)	**Approx. relative pop. size (c.2012)** [Derived from: 1,13]	**CITES: year joined**	**No. years of CITES permits (export/ import) issued (n=39; 1977-2015)**	**No. years CITES export permits issued for lion (products &/or live)**	**No. years CITES import permits issued for lion (products &/or live)**	**No. countries reported on CITES export permits (no. that are African)**	**No. countries reported in CITES import permits (no. that are African)**	**Live lion exports: no. years CITES permits issued**	**Live lion imports: no. years CITES permits issued**	**Trophy exports: no. years CITES permits issued**	**Trophy imports: no. years CITES permits issued**	**Net exports for 'trophy hunting' purposes (2001-2010)** [Source: 46]	**Av. annual wild sourced trade as % of pop. size** [Source: 13]
**EXTANT LION POPULATIONS**															
Angola	AO	S	1 970	2013	7	1	6	1(1)	3(2)	1	1	-	4	-	0.0%
**Benin**	BJ	W	215	1984	19	17	5	9	3(1)	5	5	15	4	13	1.3%
**Botswana**	BW	S	1 990	1978	39	39	29	68(18)	7(6)	12	15	36	12	115	1.4%
**Burkina Faso**	BF	W	135	1990	28	28	1	14(1)	1	-	-	27	1	53	4.9%
**Cameroon**	CM	C	217	1981	32	32	17	19(1)	4(3)	3	3	30	6	81	3.1%
**Central African Rep.**	CF	C	1 250	1980	33	33	2	17(1)	2(1)	1	1	32	1	73	<1%
Chad	TD	C	400	1989	10	8	2	3	2(1)	-	-	8	-	13	<1%
DRC	CD	C	300	1976	11	4	9	3(1)	2(2)	1	4	2	-	-	0.0%
**Ethiopia**	ET	E	1 010	1989	30	30	1	19(6)	1(1)	1	-	27	1	12	<1%
**Kenya**	KE	E	1 970	1978	38	38	22	31(6)	11(4)	10	11	17	9	1	<1%
*Malawi**	MW	S	30	1982	28	25	15	16(5)	7(5)	3	7	3	2	-	0.0%
**Mozambique**	MZ	S	2 700	1981	27	26	8	29(4)	3(3)	2	2	25	3	447	3.0%
**Namibia**	NA	S	>600	1990	37	37	39	48(7)	13(7)	9	13	36	20	196	2.5%
**Niger**	NE	W	10	1975	15	13	2	7(5)	2(2)	13	1	-	-	-	<1%
**Nigeria**	NG	W	32	1975	26	6	26	3	10(6)	-	6	-	17	-	0.0%
Senegal	SN	W	16	1977	11	7	5	4(1)	2(2)	2	3	2	1	-	0.0%
*Somalia**	SO	E	925	1986	4	4	-	3(2)	-	3	-	-	-	-	0.0%
**South Africa**	ZA	S	2 100	1975	39	39	37	138(29)	59(22)	33	30	40	36	5402	-
*South Sudan**	SS	C	580	-	-	-	-	-	-	-	-	-	-	-	<1%
*Sudan**	SD	C	unsure	1982	18	17	1	11	3(2)	8	2	8	-	-	-
**Tanzania**	TZ	E	15 600	1980	37	37	15	64(9)	6(2)	9	5	37	11	2031	2.0%
**Uganda**	UG	E	<415	1991	14	5	9	4	4(3)	1	5	2	2	-	0.0%
**Zambia**	ZM	S	1 150	1981	39	39	20	55(10)	6(5)	5	7	38	11	455	2.2%
**Zimbabwe**	ZW	S	850	1981	39	39	32	71(14)	10(8)	20	13	39	24	766	8.9%
**POSSIBLY EXTIRPATED LION POPULATIONS**															
Ghana	GH	W	0	1976	10	3	7	3	6 (5)	1	2	-	5		
Guinea	GW	W	0	1981	-	-	-	-	-	-	-	-	-		0.0%
Rwanda	RW	E	0	1981	5	3	2	3	2(1)	-	1	-	-		0.0%
Togo	TG	W	0	1978	8	2	7	1(1)	5(2)	-	4	1	2	1	>100%
**EXTIRPATED LION POPULATIONS**															
Algeria	DZ	N	0	1984	10	4	9	3	6(2)	4	9	-	-	-	-
**Burundi**	BI	E	0	1988	-	-	-	-	-	-	-	-	-	-	-
Congo	CG	C	0	1983	7	1	7	1	3(2)	-	2	1	-	-	0.0%

Cote d'Ivoire	CI	W	0	1995	16	4	14	3(1)	7(5)	-	4	3	7	-	0.0%
Djibouti	DJ	E	0	1992	-	-	-	-	-	-	-	-	-	-	-
Egypt	EG	N	0	1978	26	20	14	18(2)	9(6)	20	7	1	3	-	0.0%
Eritrea	ER	E	0	1995	1	-	1	-	-	-	-	-	-	-	0.0%
Gabon	GA	C	0	1989	20	6	19	3(1)	5(3)	2	2	1	4	2	3.0%
Gambia	GM	W	0	1977	5	-	5	-	4(1)	-	3	-	1	-	0.0%
Guinea Bissau	GW	W	0	1990	-	-	-	-	-	-	-	-	-	-	0.0%
Lesotho	LS	S	0	2003	4	1	4	1	2(2)	1	1	-	1	-	-
Libya	LY	N	0	2003	6	-	6	-	5(4)	-	5	-	-	-	-
Mali	ML	W	0	1994	4	3	1	2	1	-	1	-	-	-	-
Mauritiana	MR	W	0	1998	1	-	1	-	1(1)	-	1	-	-	-	-
Morocco	MA	N	0	1976	22	14	14	14(1)	14(2)	12	10	-	4	-	-
Sierra Leone	SL	W	0	1995	3	-	3	-	2(2)	-	-	-	2	-	-
Swaziland	SZ	S	0	1997	21	10	18	2(1)	7(7)	7	8	-	7	-	3.3%
Tunisia	TN	N	0	1975	16	8	13	6(3)	11(4)	8	12	-	-	-	-
Western Sahara	EH	N	0		-	-	-	-	-	-	-	-	-	-	-

NEVER HAD LIONS

Equatorial Guinea	GQ	C	0	1992	-	-	-	-	-	-	-	-	-	-	-
Liberia	LR	W	0	1981	5	2	3	2	2(1)	2	2	1	-	-	-

**Records for illegal hunting/ poaching** [Source: survey; 9-147]	**Illegal trade/ trafficking reported (not specific to markets)** [Source: survey; 9-147]	**Illegal trade: in markets with wildlife products** [Source: survey & literature]	**Illegal trade: live animals** [Source: survey; 9-147]	**Use: zootherapeutic (African)** [Source: survey; 9-147]	**Use: ceremonial / traditional / decorative attire** [Source: survey; 9-147]	**Use: bushmeat** [Source: survey; 9-147]	**Use: crafts/curios** [Source: survey; 9-147]	**Asian bone trade: mainly rumours/ concerns &/or limited evidence** [Source: survey; 9-147]	**Legal hunting: allowed or prohibited** [Source: 13,34,39,86,136, and others]	**Non-African foreign trade links:** questionnaire survey results only
yes	y				y	y	y	y	prohibited	Possibly Asia
yes	y	y		y	y	y	y	y	allowed (imposed 2-year moratorium in early 2000s)	
yes	y		y	y	y	y	y	y	prohibited (2001-2004; 2008 to present)	East-Southeast Asia; foreign tourists
yes	y	y		y				y	allowed	Asia
yes	y	y		y	y	y	y	y	allowed	East-Southeast Asia; Arab Gulf States
yes	y			y	y	y	y	y	allowed (imposed 3-year moratorium in early 2000s)	
yes	y			y	y	y	y	y	allowed	
yes	y			y					allowed	
probably	y			y	y		y		allowed (restricted to problem animals)	
yes	y	y	y	y	y	y	y	y	prohibited (1977) [only problem lion hunts allowed]	East-Southeast Asia; foreign tourists
yes	y	y		y	y				prohibited	
yes	y	y		y	y	y	y	y	allowed	East-Southeast Asia; foreign tourists
yes	y (insignificant)			y	y	y	y	y	allowed (no legal protection)	
-	y	y		y	y	y		y	prohibited	Asia
yes	y (massive hub)	y		y	y	y	y	y	prohibited	Asia
yes	y	y		y	y	y	y	y	allowed (restricted to problem/dangerous animals)	
yes	y	y	y						allowed [allegedly restricted to problem lions]	East-Southeast Asia; Arab Gulf States
yes	y	y	y	y	y	y	y	y	allowed	East-Southeast Asia
yes	y		y						allowed	
-			y	y	y				allowed	
yes	y (rare)	y		y	y	y	y	y	allowed	East-Southeast Asia; foreign tourists; Middle East
yes	y			y	y	y		y	allowed (resitricted to problem/dangerous lions from 2010)	
yes	y (insignificant)			y	y		y	y	prohibited (moratorium 2000-2001; 2013 to mid-2016)	East-Southeast Asia; foreign tourists
yes	y (insignificant)	y		y	y	y	y	y	allowed (but prohibited 2005-2008)	East-Southeast Asia; foreign tourists
yes	y	y		y	y	y			prohibited	
yes	y	y		y	y	y	y	y	prohibited	
-					y				prohibited	
yes	y	y		y					allowed	
-										
-										
-										

yes (was heavy)	y	y		y	y	y	y			
-										
-			y							
-										
yes	y	y		y	y	y	y			
-										
yes	y				y					
-										
-										
yes	y	y	y	y	y	y				
-										
-										
-										
-	y	y		y	y					
-										
-										
