# Supplementary material for: Questionnaire survey of the pan-African trade in lion body parts
Source: PLoS One. 2017 Oct 26;12(10):e0187060. doi: 10.1371/journal.pone.0187060 (PMC5658145; doi:10.1371/journal.pone.0187060)
Supplement: S2 Table — (PDF) [file pone.0187060.s002.pdf]

Supplementary Table 2: Questionnaire

THE TRADE AND USE IN LION BONES AND OTHER BODY PARTS ACROSS AFRICA

Welcome to the Survey

OVERVIEW

We have recently completed an assessment of the South African trade in African Lion bones and other body parts after concerns were raised that derivatives from wild lions were being sold into Traditional Asian Medicine markets, especially as a substitute for tiger. However, the trade in lion derivatives in the rest of Africa has not been documented. We are now undertaking a study of the evidence relating to the trade in lion body parts, and especially the source of parts in lion range states within the rest of Africa and, where possible, to quantify the amount of lion material that is available for sale on either local or international markets.

INVITATION

We very much hope you will take part in this study by completing this questionnaire. Your response would be greatly valued. The questionnaire can be completed anonymously if you prefer, but it would help us if you were prepared to provide your name and/or that of the organisation/institution for which you work/represent. Neither your name nor that of your organisation/institution will appear in any of the publications or reports resulting from this survey. If you wish to learn more about the results of the research we can, at your request, provide you with copies of the peer-reviewed report and other resultant publications once they are available.

The questionnaire should take less than 15 minutes to complete. We will accept partial submissions should you not wish, or be not able, to complete the entire questionnaire. The questionnaire can also be completed in French [url link] or Portuguese [url link].

Submission of the questionnaire will be taken as your consent for us to use the information you provide.

Should you have any queries or concerns about the research, please contact any member of the collaborating team:

* Dr Vivienne Williams (email address) University of the Witwatersrand
* Prof. David Macdonald (email address) & Dr Andrew Loveridge (email address) University of Oxford
* David Newton (email address) TRAFFIC East/Southern Africa

Ethics Clearance No.: H14/03/02 (University of the Witwatersrand)

Thank you for participating in our survey. Your feedback is important.

THE TRADE AND USE IN LION BONES AND OTHER BODY PARTS ACROSS AFRICA

SECTION A: Participant Information

1. What is your name

2. At what email address can you be contacted

* 3. In what country do you currently work

4. In what town/city/place/region do you currently work in?

5. Describe your occupation/area of expertise and/or who you work for

* 6. How many years have your been involved in lion conservation or relevant wildlife matters
    - Less than 2 years
    - 2 to 5 years
    - 6 to 10 years
    - 11 to 15 years
    - More than 15 years
    - Other (describe)

* 7. Which lion range states/regions do you have information for?

8. What is the scale at which you are commenting on the trade in lion derivatives?
    - National (domestic/local use/trade)
    - International
    - National and International

9. Regarding your knowledge of the trade in lion bones and derivatives, where do you know this information from? *(select as many apply)*
    - Personal observation or research
    - Scientific publications or technical reports
    - Media
    - Conversations with people who have experience (anecdotal)
    - Other (please specify)

10. Are there any publications on lion use/trade that you would like to bring to our attention (including your own)? Please list the references or the URL, OR email them to vivwill@netdial.co.za
    - Ref. 1
    - Ref. 2
    - Ref. 3
    - Ref. 4
    - Ref. 5

(* Obligatory questions)

THE TRADE AND USE IN LION BONES AND OTHER BODY PARTS ACROSS AFRICA

SECTION B: General Use and Trade of Lion Body Parts (excludes sport hunting trophies)

Please indicate your awareness of any local (domestic) or international trade in lion derivatives

* 11. For which lion body parts are you aware of local use &/or international trade?

	Local (domestic) trade	International trade	Local & international trade
1. Bones / skeletons			
2. All / most body parts			
3. Whole bodies / carcasses			
4. Skin / skin pieces			
5. Meat			
6. Fat			
7. Internal organs			
8. Limbs / paws			
9. Claws			
10. Teeth			
11. Urine			
12. Other			
If 'Other', supply details:			

12. What are the reasons why the derivatives are used/trade **LOCALLY (domestically)** ***[select as many apply]***

	Bones/skeletons	All/most body parts	Whole bodies/carcasses	Skin / skin pieces	Meat	Fat	Internal organs	Limbs/paws	Claws	Teeth	Urine
1. Food / bushmeat											
2. Crafts											
3. Curios											
4. Decorative											
5. Income generation											
6. Rituals											
7. Traditional medicine: African											
8. Magic / 'witchcraft' / supernatural											
9. Status symbol											
10. Traditional attire											
Indicate the country or countries that you are referring to:											

13. What are the reasons why the derivatives are used/trade **INTERNATIONALLY [*select as many apply*]**

	Bones/skeletons	All/most body parts	Whole bodies/carcasses	Skin / skin pieces	Meat	Fat	Internal organs	Limbs/paws	Claws	Teeth	Urine
1. Food / bushmeat											
2. Crafts											
3. Curios											
4. Decorative											
5. Income generation											
6. Rituals											
7. Traditional medicine: African											
8. Traditional medicine: Asia											
9. Magic / 'witchcraft' / supernatural											
10. Status symbol											
11. Traditional attire											
Indicate the countries between which there is trade:											

14. What is the source of derivatives in trade? **[*select as many apply*]**

	Captive: not hunted	Captive: trophy-hunt	Wild: trophy-hunt	Wild: problem lions	Wild: poaching	Wild: natural mortality	Wild: euthanasia	Taxidermist	Other	Don't know
**BONES** only										
**BODY PARTS** (*excluding bones*)										
Elaborate on a) 'other' sources; b) countries you are referring to:										

15. Evaluate the legality of the trade in lion derivatives

	Illegal	Mostly legal	Equally illegal & legal	Mostly legal	Legal	Don't know
The trade in **BONES** is						
The trade in **BODY PARTS** (*excluding bones*) is						
Comments on legal or illegal trade?						

16. What impact is the **DOMESTIC** market for derivatives having on *wild lion populations*?

	No impact	Low impact	Medium impact	High impact	Unknown impact
**BONES** use					
Countries you are referring to:					
Other **BODY PARTS** *(excluding bones)*					
Countries you are referring to:					

17. What impact is the **INTERNATIONAL** market for derivatives having on *wild lion populations*?

	Same as for domestic use	No impact	Low impact	Medium impact	High impact	Unknown impact
**BONES** use						
Countries you are referring to:						
Other **BODY PARTS** *(excluding bones)*						
Countries you are referring to:						

SECTION C: Lion Bone Trade

We have some specific questions on the lion BONE trade

18. In **Question 13** you indicated the sources of the bones. Please rank the sources in this question, where '1' is the most important source of bones, '2' is less important, etc. If one of the listed options is not a source of bones then select N/A.
Instructions. There are two ways to select rank: (i) use the *drop-down menu* next to an answer; the answer choice automatically adjusts the answer order so that rank 1 is on top; or, (ii) *drag and drop* the answer choice in order of preference.

Add rank from 1 to 8	Category	Select if appropriate
	Captive: not hunted	N/A
	Captive: trophy hunted	N/A
	Wild: trophy hunted	N/A
	Wild: problem lions	N/A
	Wild: poached	N/A
	Wild: natural mortalities	N/A
	Wild: euthanasia	N/A
	Taxidermist	N/A

* 19. Do you know anything about the supply chain for bones destined for export: *(e.g. suppliers, middlemen, exporters, importers, how the bones leave the country, etc)*

No

Yes

If YES, please supply details

* 20. Do you know anything about the quantities of bones being exported: *(e.g. number of skeletons, weight of exports, etc)*

No

Yes

If YES, please supply details

* 21. Do you know anything about the value of the bones being exported: *(e.g. price paid per skeleton, value per kilogram; differences in prices paid to people along the supply chain, etc)*

No

Yes

If YES, please supply details

* 22. Do you have information on confiscations and arrests in connection with the bone trade? *(e.g. country, when, who, where, destination, quantity, nationality of persons involved etc.* ***OR*** *references we can consult for this information* ***OR*** *persons we should contact)*

No

Yes

If YES, please supply details

* 23. Do you have information on policy and law enforcement in connection with the bone trade? *(e.g. policies and law enforcement, laws governing imports and exports and wildlife utilisation, quotas etc.* ***OR*** *where could we source this information]*

No

Yes

If YES, please supply details

24. Final comments or advice?

25. In terms of the trade in lion derivatives, which African countries are the most important to survey/investigate?
