# Supplementary material for: Questionnaire survey of the pan-African trade in lion body parts
Source: PLoS One. 2017 Oct 26;12(10):e0187060. doi: 10.1371/journal.pone.0187060 (PMC5658145; doi:10.1371/journal.pone.0187060)
Supplement: S3 Table — (HTM) [file pone.0187060.s003.htm]

**S3 Table:** Records of intra-African trade in lion body
parts and/or live lions. **Blue**
and **red**
symbols indicate records of *legal* (source: CITES Trade Database) and *illegal* (source:
questionnaire and literature surveys, personal communications) trade
respectively. Arrows point towards the receiving country from the source
country; the bi-directional symbol ⤪ indicates records
of trade in both directions. There are 131 �blocks�, of which 89 (68%)
are for legal CITES trade records only between countries, 32 (24%) are illegal
trade records only, and 10 (8%) (blue-shaded blocks)
indicate records of both legal and illegal trade between the respective
countries. (**Note:** **individual** **countries
must be read along the horizontal and vertical lines**).
Countries in differently shaded cells have either possibly extinct or extinct
wild populations.

 

|  |  |  |  |  |  |  |  |  |  |  |  |  |  |  |  |  |  |  |  |  |  |  |  |  |  |  |  |  |  |  |  |  |  |  |  |  |  |  |  |  |  |  |  |  |  |  |  |  |  |  |
| --- | --- | --- | --- | --- | --- | --- | --- | --- | --- | --- | --- | --- | --- | --- | --- | --- | --- | --- | --- | --- | --- | --- | --- | --- | --- | --- | --- | --- | --- | --- | --- | --- | --- | --- | --- | --- | --- | --- | --- | --- | --- | --- | --- | --- | --- | --- | --- | --- | --- | --- |
|  | **Country code** | AO | BJ | BW | BF | CM | CF | TD | CD | ET | KE | MW | MZ | NA | NE | NG | SN | SO | ZA | SS | SD | TZ | UG | ZM | ZW | GH | GW | RW | TG | DZ | BI | CG | CI | DJ | EG | ER | GA | GM | GW | LS | LY | ML | MR | MA | SL | SZ | TN | EH | GQ | LR |
| Angola | **AO** |  |  |  |  |  |  |  |  |  |  |  |  |  |  |  |  |  |  |  |  |  |  |  |  |  |  |  |  |  |  |  |  |  |  |  |  |  |  |  |  |  |  |  |  |  |  |  |  |  |
| Benin | **BJ** |  |  |  |  |  |  |  |  |  |  |  |  |  |  |  |  |  |  |  |  |  |  |  |  |  |  |  |  |  |  |  |  |  |  |  |  |  |  |  |  |  |  |  |  |  |  |  |  |  |
| Botswana | **BW** |  |  |  |  |  |  |  |  |  |  |  |  |  |  |  |  |  |  |  |  |  |  |  |  |  |  |  |  |  |  |  |  |  |  |  |  |  |  |  |  |  |  |  |  |  |  |  |  |  |
| Burkina Faso | **BF** |  | ← |  |  |  |  |  |  |  |  |  |  |  |  |  |  |  |  |  |  |  |  |  |  |  |  |  |  |  |  |  |  |  |  |  |  |  |  |  |  |  |  |  |  |  |  |  |  |  |
| Cameroon | **CM** |  | ⤪ | ← |  |  |  |  |  |  |  |  |  |  |  |  |  |  |  |  |  |  |  |  |  |  |  |  |  |  |  |  |  |  |  |  |  |  |  |  |  |  |  |  |  |  |  |  |  |  |
| CAR | **CF** |  |  |  |  |  |  |  |  |  |  |  |  |  |  |  |  |  |  |  |  |  |  |  |  |  |  |  |  |  |  |  |  |  |  |  |  |  |  |  |  |  |  |  |  |  |  |  |  |  |
| Chad | **TD** |  |  |  |  | ↑ |  |  |  |  |  |  |  |  |  |  |  |  |  |  |  |  |  |  |  |  |  |  |  |  |  |  |  |  |  |  |  |  |  |  |  |  |  |  |  |  |  |  |  |  |
| DRC | **CD** |  |  | ← |  |  |  |  |  |  |  |  |  |  |  |  |  |  |  |  |  |  |  |  |  |  |  |  |  |  |  |  |  |  |  |  |  |  |  |  |  |  |  |  |  |  |  |  |  |  |
| Ethiopia | **ET** |  |  |  |  | ↑ |  |  | ↑ |  |  |  |  |  |  |  |  |  |  |  |  |  |  |  |  |  |  |  |  |  |  |  |  |  |  |  |  |  |  |  |  |  |  |  |  |  |  |  |  |  |
| Kenya | **KE** |  |  | ↑ |  |  |  |  |  |  |  |  |  |  |  |  |  |  |  |  |  |  |  |  |  |  |  |  |  |  |  |  |  |  |  |  |  |  |  |  |  |  |  |  |  |  |  |  |  |  |
| Malawi | **MW** |  |  | ← |  |  |  |  |  |  |  |  |  |  |  |  |  |  |  |  |  |  |  |  |  |  |  |  |  |  |  |  |  |  |  |  |  |  |  |  |  |  |  |  |  |  |  |  |  |  |
| Mozambique | **MZ** |  |  | ⤪ |  |  |  |  |  |  | ←  ↑ | ↑ |  |  |  |  |  |  |  |  |  |  |  |  |  |  |  |  |  |  |  |  |  |  |  |  |  |  |  |  |  |  |  |  |  |  |  |  |  |  |
| Namibia | **NA** | ↑ |  | ⤪  ⤪ | ← |  |  |  |  |  | ↑ | ↑ | ← |  |  |  |  |  |  |  |  |  |  |  |  |  |  |  |  |  |  |  |  |  |  |  |  |  |  |  |  |  |  |  |  |  |  |  |  |  |
| Niger | **NE** |  | ↑  ← | ← |  |  |  |  |  |  |  |  |  |  |  |  |  |  |  |  |  |  |  |  |  |  |  |  |  |  |  |  |  |  |  |  |  |  |  |  |  |  |  |  |  |  |  |  |  |  |
| Nigeria | **NG** |  | ← | ← |  | ⤪ |  | ⤪ |  | ← |  |  |  |  | ←  ← |  |  |  |  |  |  |  |  |  |  |  |  |  |  |  |  |  |  |  |  |  |  |  |  |  |  |  |  |  |  |  |  |  |  |  |
| Senegal | **SN** |  | ← | ← | ← |  |  |  |  |  |  |  |  |  |  | ← |  |  |  |  |  |  |  |  |  |  |  |  |  |  |  |  |  |  |  |  |  |  |  |  |  |  |  |  |  |  |  |  |  |  |
| Somalia | **SO** |  |  |  |  |  |  |  |  | ← | ← |  |  |  |  |  |  |  |  |  |  |  |  |  |  |  |  |  |  |  |  |  |  |  |  |  |  |  |  |  |  |  |  |  |  |  |  |  |  |  |
| South Africa | **ZA** | ⤪ | ↑ | ⤪  ⤪ |  | ⤪ | ⤪ |  | ↑ |  | ⤪ | ⤪ | ⤪  ⤪ | ⤪  ⤪ |  | ↑ | ⤪ | ← |  |  |  |  |  |  |  |  |  |  |  |  |  |  |  |  |  |  |  |  |  |  |  |  |  |  |  |  |  |  |  |  |
| South Sudan | **SS** |  |  |  |  |  |  |  |  |  |  |  |  |  |  |  |  |  |  |  |  |  |  |  |  |  |  |  |  |  |  |  |  |  |  |  |  |  |  |  |  |  |  |  |  |  |  |  |  |  |
| Sudan | **SD** |  |  |  |  |  |  |  |  |  |  |  |  |  |  |  |  |  |  |  |  |  |  |  |  |  |  |  |  |  |  |  |  |  |  |  |  |  |  |  |  |  |  |  |  |  |  |  |  |  |
| Tanzania | **TZ** |  |  |  |  |  |  |  |  |  | ↑ |  |  | ↑ |  | ↑ |  |  | ⤪ |  |  |  |  |  |  |  |  |  |  |  |  |  |  |  |  |  |  |  |  |  |  |  |  |  |  |  |  |  |  |  |
| Uganda | **UG** |  |  |  |  |  |  |  |  |  | ← |  |  |  |  |  |  |  | ← |  |  |  |  |  |  |  |  |  |  |  |  |  |  |  |  |  |  |  |  |  |  |  |  |  |  |  |  |  |  |  |
| Zambia | **ZM** |  |  | ⤪ |  |  |  | ↑ | ↑ |  | ← | ← | ↑ | ↑ |  | ↑ |  |  | ⤪ |  |  |  | ↑ |  |  |  |  |  |  |  |  |  |  |  |  |  |  |  |  |  |  |  |  |  |  |  |  |  |  |  |
| Zimbabwe | **ZW** |  |  | ⤪ |  |  |  |  |  | ↑ | ⤪ | ⤪ | ← |  |  | ↑ |  |  | ⤪  ⤪ |  | ↑ | ⤪ | ← | ↑  ⤪ |  |  |  |  |  |  |  |  |  |  |  |  |  |  |  |  |  |  |  |  |  |  |  |  |  |  |
| Ghana | **GH** |  |  |  |  |  |  |  |  |  |  |  |  |  | ↑ |  |  |  |  |  |  | ↑ |  |  |  |  |  |  |  |  |  |  |  |  |  |  |  |  |  |  |  |  |  |  |  |  |  |  |  |  |
| Guinea | **GW** |  | ← |  | ← |  |  |  |  |  |  |  |  |  | ← | ← | ← |  |  |  |  |  |  |  |  |  |  |  |  |  |  |  |  |  |  |  |  |  |  |  |  |  |  |  |  |  |  |  |  |  |
| Rwanda | **RW** |  |  |  |  |  |  |  |  |  |  |  |  |  |  |  |  |  |  |  |  |  |  |  |  |  |  |  |  |  |  |  |  |  |  |  |  |  |  |  |  |  |  |  |  |  |  |  |  |  |
| Togo | **TG** |  | ← |  | ← |  |  |  |  |  |  |  |  |  |  |  |  |  | ↑ |  |  |  |  |  |  | ← |  |  |  |  |  |  |  |  |  |  |  |  |  |  |  |  |  |  |  |  |  |  |  |  |
| Algeria | **DZ** |  |  |  |  |  |  |  |  |  |  |  |  |  |  |  |  |  |  |  |  |  |  |  |  |  |  |  |  |  |  |  |  |  |  |  |  |  |  |  |  |  |  |  |  |  |  |  |  |  |
| Burundi | **BI** |  |  |  |  |  |  |  |  |  |  |  |  |  |  |  |  |  |  |  |  |  |  |  |  |  |  |  |  |  |  |  |  |  |  |  |  |  |  |  |  |  |  |  |  |  |  |  |  |  |
| Congo | **CG** |  |  |  |  |  |  |  |  |  |  |  |  |  | ← |  |  |  | ← |  |  |  |  | ← |  |  |  |  |  |  |  |  |  |  |  |  |  |  |  |  |  |  |  |  |  |  |  |  |  |  |
| Cote d'Ivoire | **CI** |  | ← | ← | ← |  |  |  |  | ← |  |  |  |  |  | ← |  |  | ⤪ |  |  |  |  |  |  |  |  |  |  |  |  |  |  |  |  |  |  |  |  |  |  |  |  |  |  |  |  |  |  |  |
| Djibouti | **DJ** |  |  |  |  |  |  |  |  |  |  |  |  |  |  |  |  |  |  |  |  |  |  |  |  |  |  |  |  |  |  |  |  |  |  |  |  |  |  |  |  |  |  |  |  |  |  |  |  |  |
| Egypt | **EG** |  |  | ← |  |  |  |  |  |  |  |  |  |  |  | ← |  |  | ⤪ |  | ↑ | ⤪ |  | ← | ← |  |  |  |  |  |  |  |  |  |  |  |  |  |  |  |  |  |  |  |  |  |  |  |  |  |
| Eritrea | **ER** |  |  |  |  |  |  |  |  | ← |  |  |  |  |  |  |  |  |  |  |  |  |  |  |  |  |  |  |  |  |  |  |  |  |  |  |  |  |  |  |  |  |  |  |  |  |  |  |  |  |
| Gabon | **GA** |  | ← | ← |  |  |  |  |  | ← |  |  |  |  | ← |  |  |  | ← |  |  |  |  |  |  |  |  |  |  |  |  |  |  |  |  |  |  |  |  |  |  |  |  |  |  |  |  |  |  |  |
| Gambia | **GM** |  |  |  |  |  |  |  |  |  |  |  |  |  |  |  |  |  | ← |  |  |  |  |  |  |  |  |  |  |  |  |  |  |  |  |  |  |  |  |  |  |  |  |  |  |  |  |  |  |  |
| Guinea Bissau | **GW** |  |  |  |  |  |  |  |  |  |  |  |  |  |  |  |  |  |  |  |  |  |  |  |  |  |  |  |  |  |  |  |  |  |  |  |  |  |  |  |  |  |  |  |  |  |  |  |  |  |
| Lesotho | **LS** |  |  | ← |  |  |  |  |  |  |  |  |  |  |  |  |  |  | ⤪ |  |  |  |  |  |  |  |  |  |  |  |  |  |  |  |  |  |  |  |  |  |  |  |  |  |  |  |  |  |  |  |
| Libya | **LY** |  |  |  |  |  |  |  |  |  |  |  |  |  |  |  |  |  | ← |  |  |  |  |  | ← |  |  |  |  |  |  |  |  |  |  |  |  |  |  |  |  |  |  |  |  |  |  |  |  |  |
| Mali | **ML** |  |  |  | ← |  |  |  |  |  |  |  |  |  | ← |  | ← |  |  |  |  |  |  |  |  |  | ⤪ |  |  |  |  |  |  |  |  |  |  |  |  |  |  |  |  |  |  |  |  |  |  |  |
| Mauritiana | **MR** |  |  |  |  |  |  |  |  |  |  |  |  |  |  |  | ← |  |  |  |  |  |  |  |  |  |  |  |  |  |  |  |  |  |  |  |  |  |  |  |  |  |  |  |  |  |  |  |  |  |
| Morocco | **MA** |  |  |  |  |  |  |  |  |  |  |  |  |  |  |  |  |  | ← |  |  |  |  |  |  |  |  |  |  |  |  |  |  |  |  |  |  |  |  |  |  |  |  |  |  |  |  |  |  |  |
| Sierra Leone | **SL** |  |  |  |  |  |  |  |  |  |  |  |  | ← |  |  |  |  | ← |  |  |  |  |  |  |  |  |  |  |  |  |  |  |  |  |  |  |  |  |  |  |  |  |  |  |  |  |  |  |  |
| Swaziland | **SZ** |  |  | ← |  |  |  |  |  |  | ← | ← |  | ← |  |  |  |  | ⤪  ← |  |  | ← |  |  | ← |  |  |  |  |  |  |  |  |  |  |  |  |  |  |  |  |  |  |  |  |  |  |  |  |  |
| Tunisia | **TN** |  |  |  |  |  |  |  |  |  |  |  | ← |  |  |  |  |  | ← |  |  |  |  |  |  |  |  |  |  | ↑ |  |  |  |  |  |  |  |  |  |  |  |  |  | ← |  |  |  |  |  |  |
| W. Sahara | **EH** |  |  |  |  |  |  |  |  |  |  |  |  |  |  |  |  |  |  |  |  |  |  |  |  |  |  |  |  |  |  |  |  |  |  |  |  |  |  |  |  |  |  |  |  |  |  |  |  |  |
| E. Guinea | **GQ** |  |  |  |  |  |  |  |  |  |  |  |  |  |  |  |  |  |  |  |  |  |  |  |  |  |  |  |  |  |  |  |  |  |  |  |  |  |  |  |  |  |  |  |  |  |  |  |  |  |
| Liberia | **LR** |  |  |  |  |  |  |  |  |  |  |  |  |  |  |  |  |  | ← |  |  |  |  |  |  |  |  |  |  |  |  |  |  |  |  |  |  |  |  |  |  |  |  |  |  |  |  |  |  |  |
