# Supplementary material for: Questionnaire survey of the pan-African trade in lion body parts
Source: PLoS One. 2017 Oct 26;12(10):e0187060. doi: 10.1371/journal.pone.0187060 (PMC5658145; doi:10.1371/journal.pone.0187060)
Supplement: S1 Doc — (PDF) [file pone.0187060.s004.pdf]

SUPPORTING INFORMATION S1 DOCUMENT

SUB-REGIONAL SUMMARIES

The information contained herein is a lengthier account of informant opinion on sub-regional trade and use, and includes quotes captured in the comments sections of several questions.

1) Sub-regional expertise

The sub-regional levels of respondent expertise and capacity is summarised in S1A Table. Most respondents had ≥11 years of experience in carnivore conservation and/or relevant wildlife matters and worked for a range of academic and non-academic institutions.

S1A Table: Regional capacity: percentage of respondents in the most frequently listed categories of experience and expertise

Region	% people ≥11 years relevant experience	Occupation: % per category
Southern	53%	57% - carnivore conservation (academic & non-academic institutions)<br>37% - general wildlife conservation (academic & non-academic institutions)
East	70%	60% - carnivore conservation (academic, non-academic, government institutions)<br>30% - general wildlife conservation (academic & non-academic institutions)
West	72%	57% - wildlife conservation (academic & government institutions)<br>43% - carnivore conservation (non-academic institutions)
Central	75%	50% each - carnivore and wildlife conservation (academic & government institutions)
South	71%	31% - general wildlife conservation (academic, non-academic, government institutions)<br>23% - carnivore conservation (non-academic institutions)

2) Sub-regions

a) Southern Africa (excluding South Africa)

Informant opinion was that the domestic use and trade of body parts in Southern Africa mostly involved skin and claws, teeth and fat (Fig. 4a; Fig. 5a; Table 3; Fig. A) for the craft/curios African zootherapeutic purposes. International trade, however, was perceived to be more for bones, claws and teeth for zootherapeutic practices (African and Asian), income generation, and crafts/curios (Fig. 4b; Fig. 5b; Table 3; Fig. B).

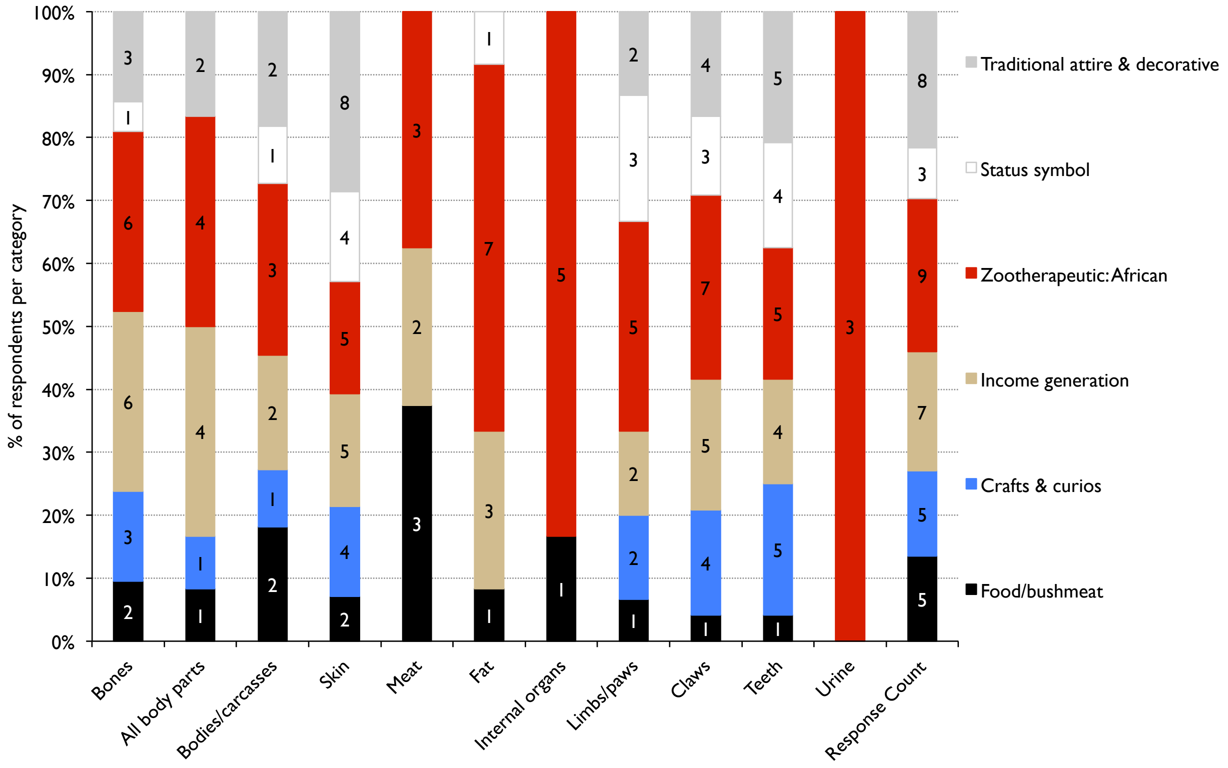

Fig. A. Reasons for domestic trade in Southern Africa (excluding South Africa)

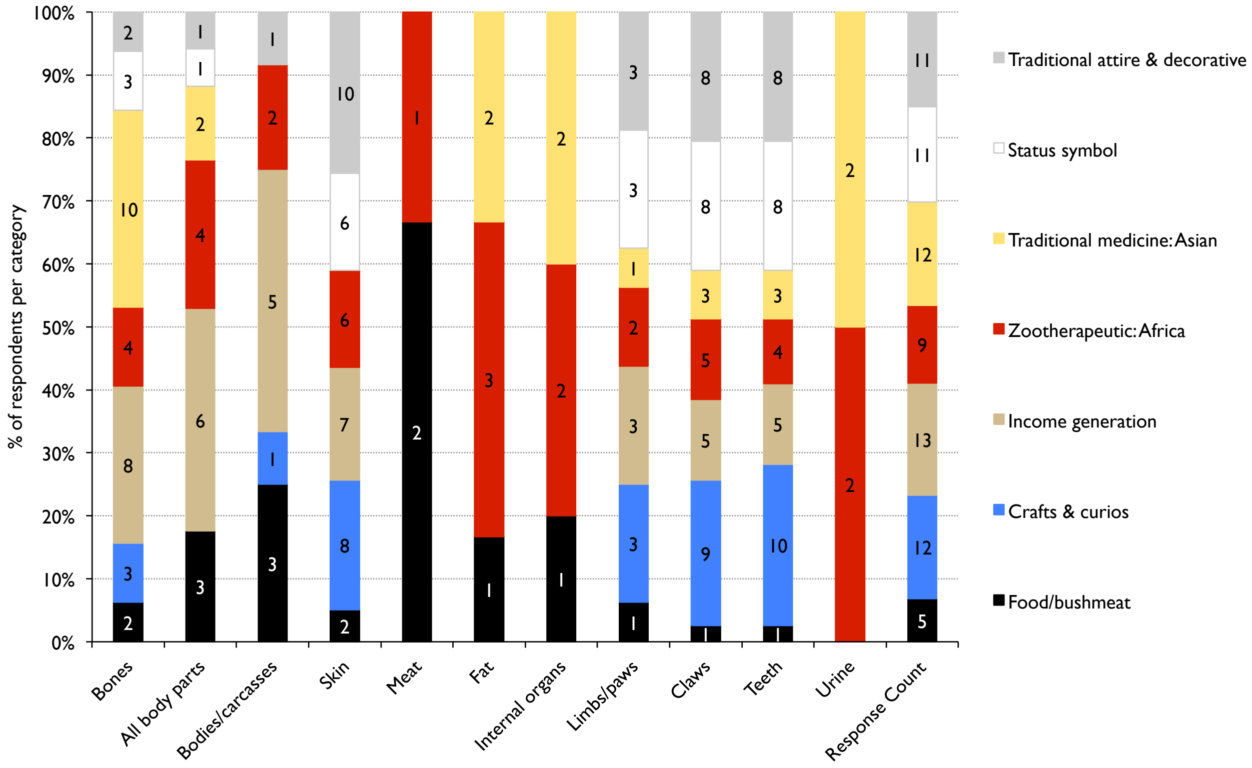

Fig. B. Reasons for international trade in Southern Africa (excluding South Africa)

The results for the perceived legality of trade are excluded from the results, but the following explanatory responses were received for four countries in the comments of Question 15:

Mozambique: Two respondents wrote: (Resp't #63) Trade is legal, and in Mozambique the lion is not a protected species by law, so it can be hunted and sold. In Mozambique the lion is in Appendix II of CITES, so the international trade of its products is allowed as long as it is regulated [original answer in Portuguese: *Em Mocambique o leão não 'e uma espécie protegida por Lei, portanto pode ser caçado e comercializado. Em Mocambique o leão está no Apêndice II da CITES, portanto o comercio internacional dos seus produtos 'e permitido, desde que regulado*]; (Resp't #12) The trade is illegal, and "*lions are killed in bushmeat snares and skins are taken and sold but lions were killed inadvertently, not targeted just used when available*".

Namibia: Two respondents wrote: (Resp't #56) Trade is mostly legal. "*The skin of a problem lion that has been killed is permitted by the Ministry of Environment for sale so that the owner of livestock that has been killed by the lion can sell the skin for a profit and make up some of his financial losses*"; (Resp't #15) Trade is illegal. "*The only legal trade I know of is sport hunting trophies. However, I am not sure whether Namibia has any specific laws about trading body parts, other than that killing a protected species without declaring it and selling any part of it without a permit of is illegal. I am also not sure about what happens to the non-exported body parts of lions that are trophy hunted*".

Zambia: Resp't #4 wrote: Trade is illegal, "*in particular, lion fat is prized and often harvested from problem animal carcasses. Although technically illegal, ZAWA* [Zambian Wildlife Authority] *turns a blind eye and is very likely harvesting fat themselves from at least some carcasses. I found freshly dead problem lion carcasses that had been harvested for fat behind ZAWA offices. When I queried this, ZAWA officers simply shrugged. I was even asked by some officers if I could obtain fat for them (!). Lion floating bones (clavicles) are also highly prized*".

Zimbabwe 1, with the opinion that trade was mostly illegal, four respondents wrote: (Resp't #43) "*I have been offered lion teeth and claws in curio markets in Victoria Falls and no permit or certificate was offered with the items*"; (Resp't #18) "*There needs to be better definitions on a local level, especially in terms of problem animal control as to how the carcass is distributed and ensuring that local consumption doesn't then drive into international trade*"; (Resp't #17) "*Information received that from PAC* [Problem Animal Control] *and Poached lions local trade with Chinese workers at Hwange Coal mines and Airport construction* [Victoria Falls Airport] – *all illegal. Suspected trade from dubious hunters via South Africa to Asia (not confirmed). Captive breeding operations of lions in South Africa also suspected of trading in body parts. (Some Zimbabwe captive bred lions may go through this system (not confirmed)*"; (Resp't #32) "*It is illegal to sell or trade any animal body parts unless if you are holding a permit issued in terms of Parks and Wildlife Authority, or unless if you are a taxidermist*".

Zimbabwe 2, with the opinion that trade is all illegal, four respondents wrote: (Resp't #42) "*The illegal trade has grown with more Orientals in the region*"; (Resp't #30) "*I do not believe the Zimbabwean legislation allows any export other than of trophy hunted lions which makes any trade of lion parts across its boarders illegal*"; (Resp't #29) "*Trophy hunting is legal and clients can still export/import trophies from the countries in which lion are hunted and bring them back to their country of origin. However, lion parts cannot be moved across borders and traded and permits are required to move any lion body parts out of Zimbabwe*"; (Resp't #19) "*This aspect of the lion trade is I believe at a low level in Zim but I do believe it is involved in certain quarters. WHERE THERE IS BIG MONEY TO BE MADE THERE WILL BE PEOPLE INVOLVED*".

One researcher (Resp't #34) working across Botswana, Mozambique, Zambia and Zimbabwe remarked that lion trade is mostly legal and "*at this stage I am not aware of any poaching of lions specifically for body parts or for trade in such body parts in any of the countries in which we have (typically law enforcement/poaching related) projects - however, that is not to say it doesn't happen. Except for legal hunting trophies*".

Regarding the sources of body parts (excluding bones), most informants were of the opinion that they acquired from wild problem lions (n=16), wild lions that are poached (n=14) and trophy hunted lions

(captive and wild, n=15 combined) (Fig. 6; Table 3). In the comments of Question 14, the following responses were received: (Resp't #2, Botswana): "*Lion carcasses and bones are being smuggled from Botswana into South Africa*"; (Resp't #4, Zambia): "*There is increasing pressure in Zambia to allow for captive-bred lions to be kept, hunted or otherwise traded or utilized for profit. I strongly suspect that this activity already occurs (illegally) on some level in Zambia*"; (Resp't #29, Zimbabwe): "*My feeling is that the majority of lion parts being traded are coming from South Africa through captive bred lions. However, there is growing evidence and circulating rumours about several lion breeders in Zimbabwe trading lion parts (whole carcasses). The speculation is that these are first being moved to South Africa*"; and further, they wrote "*I think the trade in lion parts is relatively small and there are a large number of captive lions to feed the market. However, as dealers continue to grow the market it could begin to threaten wild populations which can be fairly easily poached if targeted*"; (Resp't #54, Southern Africa): "*Wild-poaching source: Lion, leopard and smaller cat skins are exported to South Africa for traditional and or status and decorative attire*".

The domestic trade in body parts was mostly perceived to be having 'low' (n=16, 33% of responses) or 'no impact' (13% responses) on wild lion populations in Southern Africa (Fig. 7; Table 3). However, others also said this impact was 'unknown' (27%) or 'high' (15%). Of the sub-regions, Southern Africa had the largest proportion of 'no impact' responses. A respondent (Resp't #5) from Botswana said that trade was having a low impact and that they were not "*aware of any case where people actively hunted lion for its parts. Rather, commonly people use problem killed or naturally mortality carcasses*", whereas Resp't #30 from Zimbabwe said that they were unsure of the impact because they were unaware of a domestic market for lion body parts. Unlike the domestic trade, the impact of international trade was mostly deemed to be unknown (48% responses, especially Angola, Mozambique and Zambia) or high (21%, especially Botswana and Zimbabwe; Fig. 7; S5 Fig.). However, 13% felt the international trade has no impact.

Zimbabwe, Mozambique and Zambia were nominated the most as priorities for further study (Fig. 2; S6 Fig.). These countries were nominated because: (i) they have large lion populations; (ii) they are a potential source of large numbers of carcasses because of trophy hunting, problem lion control and lion breeding; (iii) they are 'hotspots' for poaching; (iv) there are unknown levels of illegal wildlife trade in some areas, and only certain aspects are reported (e.g. lion skins are exported but the fate of the skeletons is unknown); (v) local markets offer a diversity of lion products to local buyers; and (vi) parts such as teeth, claws and skin are sold openly to tourists in some markets for souvenirs and curios, but the products were likely to be obtained from poached animals or lions killed in retribution for stock raiding or lions killed in road accidents. Incidents of purposeful poisoning of lions were also said to be escalating in the region (especially Mozambique), and this has reportedly precipitated a whole new threat to lions for a market that allegedly cannot be satiated. In a subsequent email, Resp't #51 tendered that South Africa is responsible for this surge in illegal activity because it facilitated the legal trade in lion bones, which led to an overnight awareness of a market for lion parts/products, which triggered the 'copycat' activities in neighbouring countries thereafter.

b) East Africa

Domestic use of claws (n=11) and teeth (n=10) is believed to be more prevalent in East Africa, but more moderate for fat (n=8) and skin pieces (n=7) (Fig. 4a; Fig. 5a; Table 3; Fig. C). In contrast, there were more responses for international trade in urine (allegedly from lions) (n=11) from East Africa to other countries (Fig. 4b) rather than equivalent domestic use of the same product (n=2; Fig. 4a). Overall, the mentions of international trade in lion body parts here was surprisingly limited relative to domestic use (Fig. 4b; Fig. 5b; Table 3; Fig D), but a factor could also be the small sample size. Given that claws and teeth are periodically intercepted by airport customs officials in East Africa [132] and publicised in the media, more responses were expected. In the comments of Question 11, Resp't #7 noted that there is also a domestic trade in lion hairballs in Kenya, while Resp't #14 said that lion manes and tails are traded in Tanzania domestically and internationally.

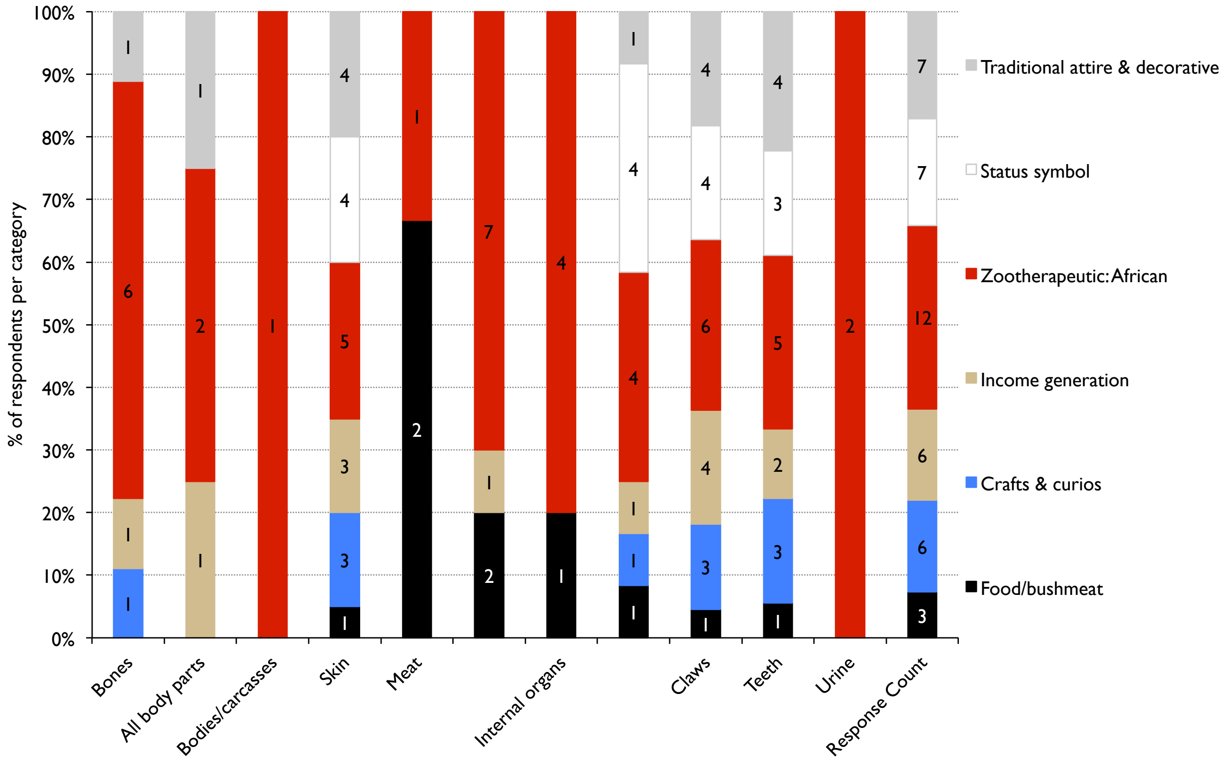

Fig. C. Reasons for domestic trade in East Africa

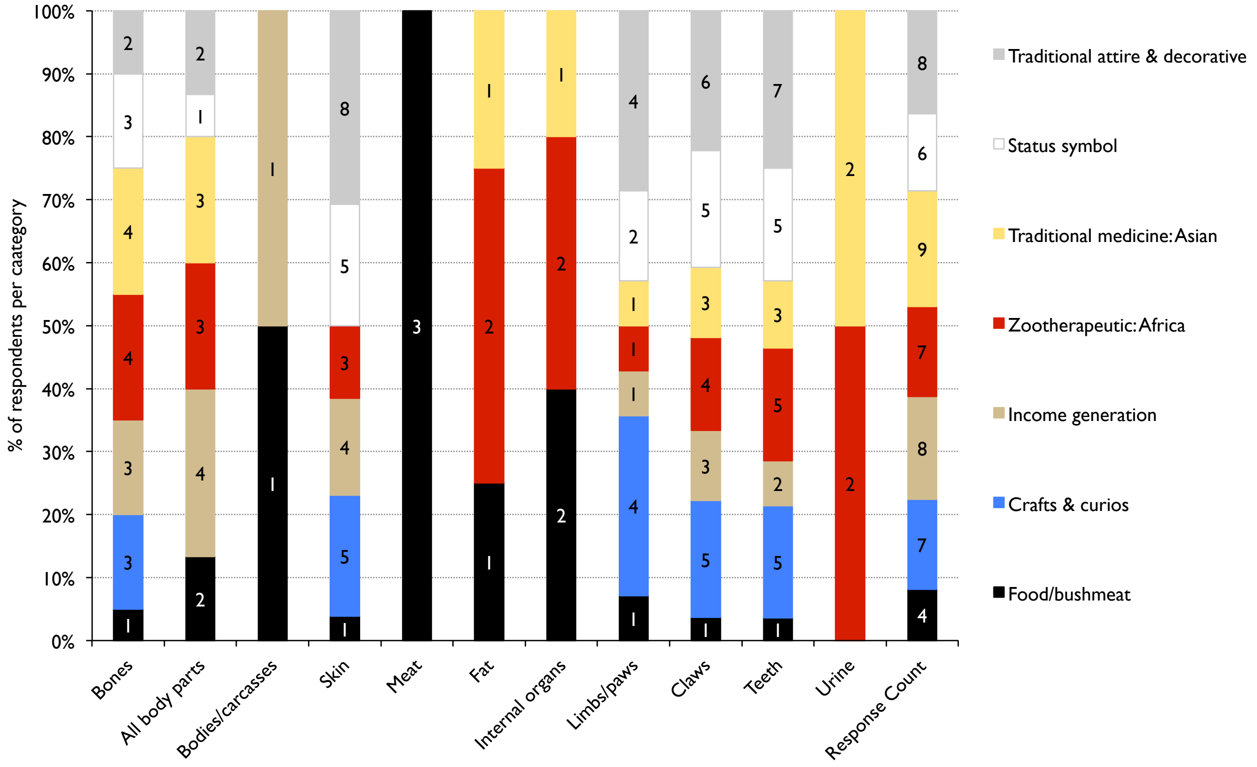

Fig. D. Reasons for international trade in East Africa

Domestic use in East Africa is believed, like Southern Africa, to be weighted towards zootherapeutic practices (n=12, 29% responses) (Fig. C; Table 3), and most body parts were listed for this purpose (especially fat, claws and bones). Informant opinion was that the domestic market for crafts/curios, and

traditional attire, usually involves claws and skin, and these parts plus teeth were the parts most often cited overall. In the comments of Question 12, Resp't #39 said that while lion fat is mainly used in West Africa "*poached carcasses in Kenya and Tanzania also show* [signs of] *fat harvesting recently*". Zootherapeutic practices, both Asian and African, were also said to be the main reasons for the international trade (n=16, 33% combined of total responses) (Fig. D; Table 3) and involved mainly lion bones. The craft/curio market for claws, teeth and skin (but also involving lion limbs and paws from which the claws are removed) was another frequently nominated reason for international trade.

The results for the perceived legality of trade are excluded from the results, but the following three explanatory responses were received for Tanzania in the comments of Question 15:

(Resp't #50) Trade is illegal and *"involves lions killed on the fringes of protected areas or those that wander incidentally into village lands"*;

(Resp't #41) Trade is mostly illegal and *"people can own body parts but they need a permit and so trade is usually not legal"*;

(Resp't #14) Was uncertain of the legality and "*I have only heard of a single case where lion parts were believed to go to China in the past 40 yrs. Most lion trophies are exported via CITES. An uncertain number of canine teeth and claws are sold to foreign tourists*".

Body parts obtained from lions in the sub-region were considered to be from four sources: wild lions that were poached (n=7), wild problem lions (n=6), trophy hunted wild lions and natural mortality of wild lions (n=4 each) (Fig. 6). A respondent (Resp't #50) from Tanzania further noted: "*All* [body parts sourced for trade are] *from poached lions under the disguise/pretext of problem lions…from Tanzania*".

Respondents were divided on the impact that domestic trade of body parts (excluding bones) is having on wild lion populations in East Africa; 32% said the impact was 'unknown', 37% that it was 'medium' to 'high', and 32% that there was 'little' to 'no impact' (Fig. 7). The international trade in body parts was mostly considered to be having an unknown impact (79% of responses), but there were also answers for this impact being both high and low (Fig. 7). Tanzania was the only East African country where domestic and international trade was said to be having a high impact on wild populations (S4 & S5 Figs.).

Tanzania and Kenya were nominated the most as priorities for further study (Fig. 2; S6 Fig.), mainly because: (i) there is a high level of poaching; (ii) there is a history of illegal trade, and countries like Tanzania, Kenya and Uganda are transit routes for illegal wildlife trade; (iii) like Southern Africa, lion parts such as teeth, claws and skin are sold openly to tourists for souvenirs and curios; (iv) Tanzania has an established trophy hunting industry that might be a conduit for illegal trade, especially through hunting operators looking to generate additional income; and, (v) the trade in lion parts is growing. Resp't #50 wrote: "*up to 200 lions may be killed annually by traditional pastoralists around Tanzania. A study should also investigate whether or not bones are traded in one form or another*". While Uganda was infrequently mentioned overall during the survey, a respondent wrote in a subsequent email to us that a game warden from the Queen Elizabeth National Park had been approached by somebody to find out if they could sell lion body parts.

c) West Africa

Skin pieces (n=24 responses), bones (n=21), claws and urine (n=20 each), teeth and fat (n=19 each), were the parts believed to be most used domestically (Fig. 4a; Fig. 5a; Table 3; Fig. E). But whereas respondents recorded urine to be more likely used locally, it was less likely to be traded internationally (n=4; Fig. 4b; Fig. 5b; Fig. F.), presumably because it is imported rather than exported. Of the parts traded internationally, there were more citations for bones (n=17), teeth (n=8), skin and claws (n=7 each) (Fig. 4b; Fig. 5b).

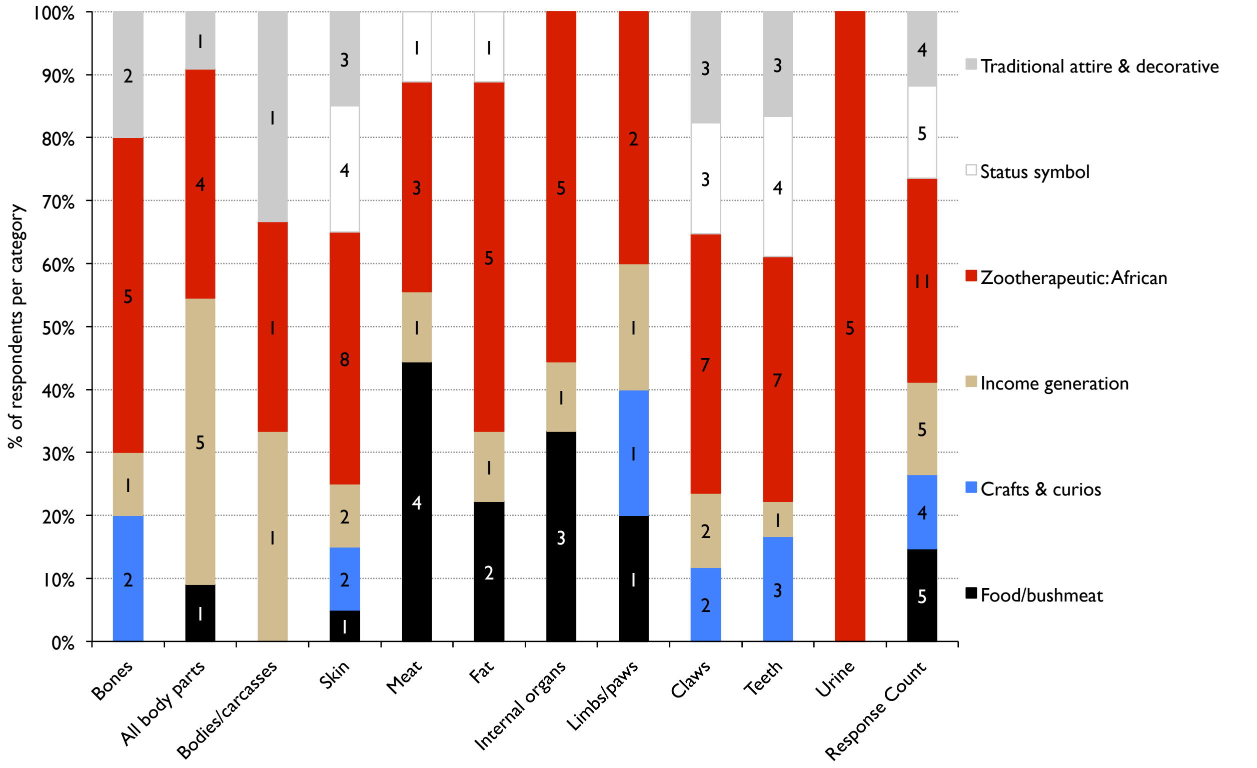

Fig. E. Reasons for domestic trade in West Africa

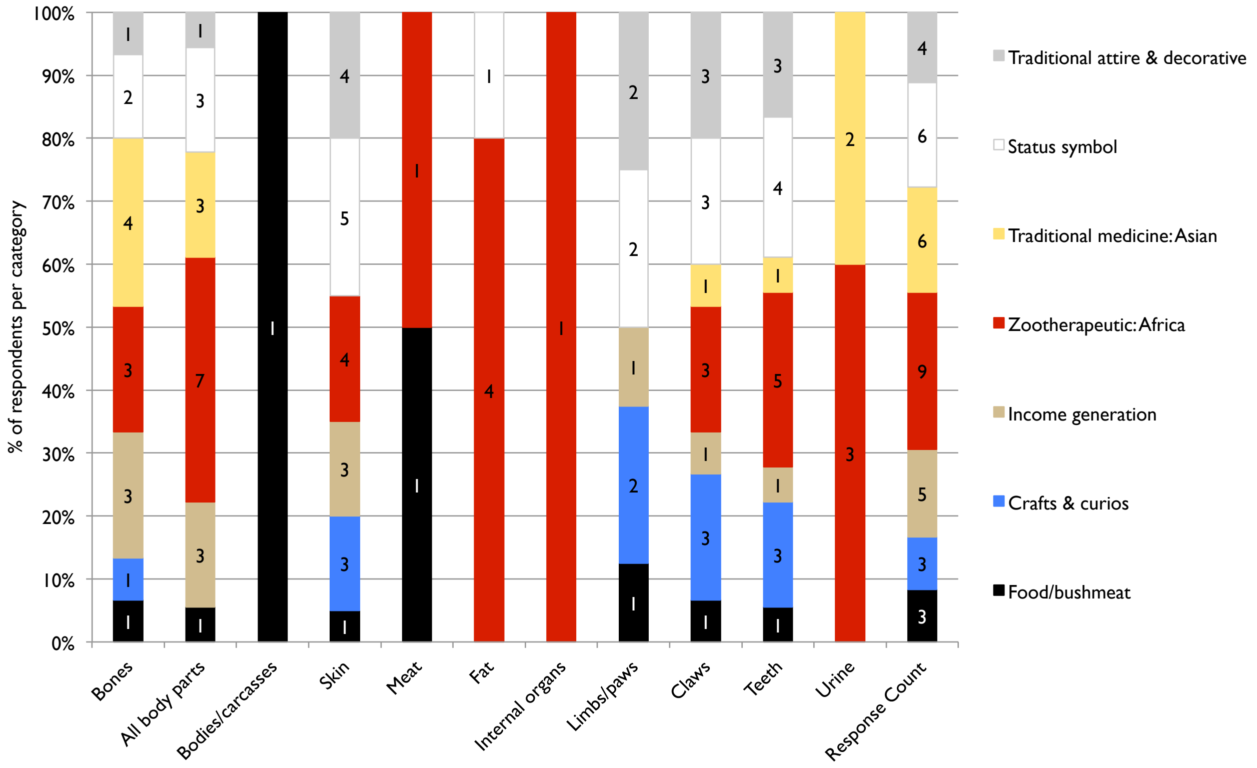

Fig. F. Reasons for international trade in West Africa

Domestic utilisation in West Africa was, like other sub-regions, believed to be centred around African zootherapeutic practices (n=11, 32% responses) (typically skin, claws and teeth) (Table 3; Fig. E). Parts intended for traditional attire and status symbols (n=9 combined) were also important. The international trade was mainly said to be for zootherapeutic purposes (African and Asian) (n=15 combined, 42%) (particularly African), with some trade related to status symbols (n=6) (Fig. F). Lion skins were nominated as the part most often in international trade, especially for traditional attire (n=5; Fig. F).

The results for the perceived legality of trade are excluded from the results, but the following three explanatory responses were received in the comments of Question 15:

(Resp't #16) Was uncertain of the legality and *"In Benin and Burkina-Faso cases of lion derivatives arrested are not considered legal even though the correct legislations are unknown and taxidermist and local communities who work with park managers should have the right to sell or do whatever they want with their part of derivatives (from legal trophy hunting)"*;

(Resp't #62) The lion is a fully protected species in the Republic of Guinea. Slaughter and trade of all kind of derivative are illegal (original answer in French: *"Le lion est une espèce intégralement protégée en République de Guinée. Son abattage et le commerce de tout produit dérivé de l'espèce sont illégaux"*);

(Resp't #10) in reference to Benin, Cote d'Ivoire, Guinea, Nigeria, Senegal: *"I am not fully aware of the legislation for the different countries I refer to. However, I strongly suspect that trade is illegal in most countries I cover"*.

Lion body parts were mainly believed to come from wild lions that are poached (n=6; Fig. 6; Table 3). A Nigerian respondent (Resp't #60) wrote that poached lions are brought into Nigeria (likely originating from neighbouring countries), but that these events have declined with the 'insecurity' that is rife in northern Nigeria (original answer in French: *"Les lions qu'i sont braconnés étaient amenés au Nigéria. Ce phénomène a considérablement diminué avec l'insécurité qui sévit dans le Nord Nigérian"*). Body parts from trophy hunted lions are also in the domestic and international trade (n=4; Fig. 6). As Resp't #10, commenting on the West and Central African lion trade, wrote: *"…origin of body parts from trophy hunting are suspected in Benin and Burkina Faso. Origin from the wild are proven in Benin, Nigeria, Cameroon, and strongly suspected in Senegal, formerly in Cote d'Ivoire (lions now extinct), Burkina Faso and formerly in Guinea (lions now most likely extinct)"*. Resp'ts #10 & #16 also noted that lion urine sold in Nigeria is taken from captive lions in a Niger zoo (see also [134]).

The impact of the domestic trade of body parts was mainly considered 'unknown' (40% of responses, Fig. 7; Table 3); 28% consider this impact to be 'high' and 32% 'low'. Countries where the impact of some domestic trade was nominated as high, low and unknown are Benin, Burkina Faso, Nigeria, Senegal and Guinea (the lion population in the latter country is possibly extinct) (S4. Fig.). The international trade in lion body parts (excluding bones) in the region is, like the rest of Africa, mostly considered to be having an unknown impact on wild lion populations in range states (69% responses, Fig. 8b). However, 27% suggested that the impact was high (Fig. 7) – especially in Nigeria (S5 Fig.). Other countries nominated at least once for international trade having a high impact are Benin, Burkina Faso, Senegal and Guinea (S5 Fig.).

The active and diverse local market for lion body parts in West Africa (especially for zootherapeutic practices), and the small and declining lion populations, validates the need for further studies be conducted in the sub-region, especially in Nigeria and possibly also Benin and Burkina Faso (Fig. 2; S6 Fig.). There was also a concern about the limited data available on trade relative to the alleged importance of local trade. Nigeria only has a small lion population, but the trade in derivatives there allegedly "*fuels the market*" (Resp't #33). Furthermore, trophy hunting in Benin and Burkina Faso are having an impact on lion populations and generating a body part surplus (after the trophy parts have been removed) that can be traded regionally. Resp't #10 from Gabon wrote: "*More detailed field studies covering traditional markets in West and Central Africa, as well as local and international supply chains* [are needed]. *Based on the results, there is a need for local awareness-raising and provisioning of direct support to curb trade.*

Considering low lion numbers in West and Central Africa, this local and international trade is sufficiently important to wipe out some of the last remaining populations".

d) Central Africa

There was a smaller sample size for Central Africa compared to the other sub-regions, and more citations of domestic versus international trade (Figs 4a,b). The latter, however, received more citations for urine awareness (n=19) than other body parts (teeth, skin and claws had n=5, 5 & 4 respectively) (Fig. 4b). Of the products believed to be in local use, teeth and fat (n=8 each) and claws and meat (n=7 each) were better known (Fig. 4a; Table 3).

Like all the other sub-regions, informant opinion was that domestic utilisation was mainly for zootherapeutic purposes (n=7, 37%) (Fig 5a; Table 3; Fig. G), particularly the teeth, claws, skin and bones. The reasons for international trade in lion body parts was also said to be mainly for zootherapeutic purposes (African and Asian, n=11 combined, 35%), with skins important for traditional attire and status symbols (Fig 5b; Table 3; Fig. H).

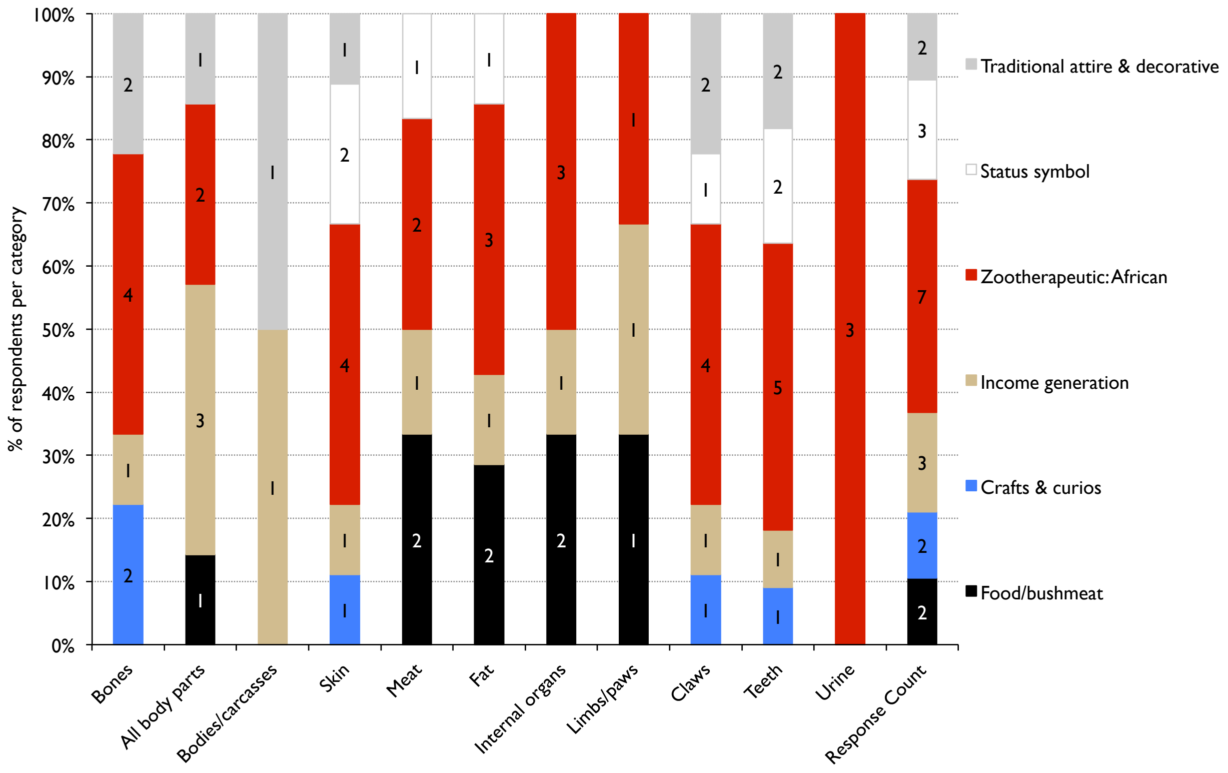

Fig. G. Reasons for domestic trade in Central Africa

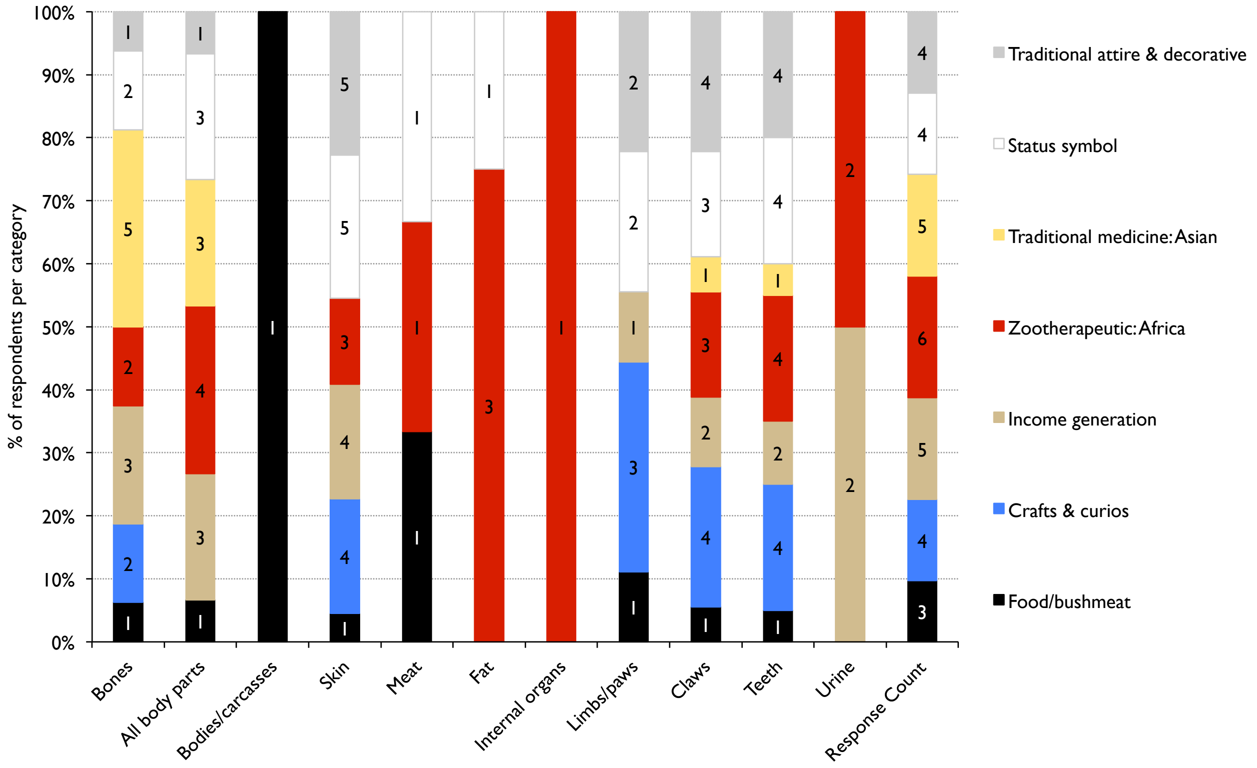

Fig. H. Reasons for international trade in Central Africa

The results for the perceived legality of trade are excluded from the results, but the following three explanatory responses were received for Cameroon in the comments of Question 15:

(Resp't #60) Legal trade is possible if the lion population is sizeable. However, there are estimated to be more than 20 lions in the Waza National Park, which cannot be the subject of legal trade (original answer in French: "*Le commerce légal ne peut se faire que si la population des lions est considérable. Le nombre que l'on estime à plus de 20 dans le Parc National de Waza ne saurait faire l'objet d'un commerce légal*");

(Resp't #1) Although the government of Cameroon (through the Ministry of Wildlife) authorises trophy hunting, most of the lion hunting is illegal;

(Resp't #53) "*I have evidence of body parts of lions killed for trophy hunting in Cameroon are sold on the black market. A case was discovered by the NGO ALERT in Cameroon. We also lost more than five collared lions in Waza NP during 2007–2009, which were killed, collars cut off with a knife and carcasses disappeared mysteriously*" (see also [133]).

There were only five responses on the origin of lion body parts, relating to Cameroon and Gabon. Poaching and trophy hunting (n=2), and problem lions (n=1), were listed as the sources of parts in this region (Fig. 6).

The impact of the domestic trade in lion body parts (excluding bones) was mostly said to be 'unknown' (n=7, 39% of responses), although 33% perceived the impact to be 'low', 22% 'medium', and 6% 'high' (Fig. 7). Cameroon was the only country nominated for some domestic trade having a high impact on lion populations, whereas all other range states had responses for low, medium or unknown impact (S Fig.). Regarding international trade, respondents were most uncertain on the impact to wild populations in Central Africa compared to other sub-regions (n=15, 83% of responses, Fig. 8b), but trade was perceived to be having a high impact in Cameroon and the CAR (S5 Fig.).

Regarding further studies in the Central Africa, Cameroon and CAR were nominated (Fig. 2; S6 Fig.) for reasons relating to the trophy hunting of wild lions. However, lion trade and utilisation is the least documented here of all sub-regions, and this makes it a priority for conducting further research to determine levels of trade and the impacts thereof.

e) South Africa

The South African trade in lion body parts was best known to respondents, largely through the research being actively conducted there and incidences of illegal trade reported by the media. Domestic trade was said to be more for skin, claws and teeth (n=11, 10 & 8 respectively) (Fig. 4a; Table 3), and incidences of poaching reported in the media South Africa in 2016 would affirm this. Whilst international trade in lion bones derived mainly from captive animals is well known and documented, regular trade in claws, skin and teeth was also understood to occur (Fig. 4b; Table 3).

Local utilisation in South Africa was, like the rest of Africa, understood to be mostly for zootherapeutic purposes (n=9, 24%) but involving 'lion' fat, claws and bones (Fig. 5a; Table 3; Fig. I). Owning lion skin and teeth for traditional attire and as a status symbol are also important to some communities, although skins from spotted carnivores tends to be preferred for this purpose [119]. The international trade in body parts is known to be dominated by the East-Southeast Asian lion bone trade for traditional Asian medicine (Fig. 5b.; Table 3; Fig. J). However, skins used for traditional attire, status symbols, crafts/curios and for income generation were also reasons nominated for international trade Table 3).

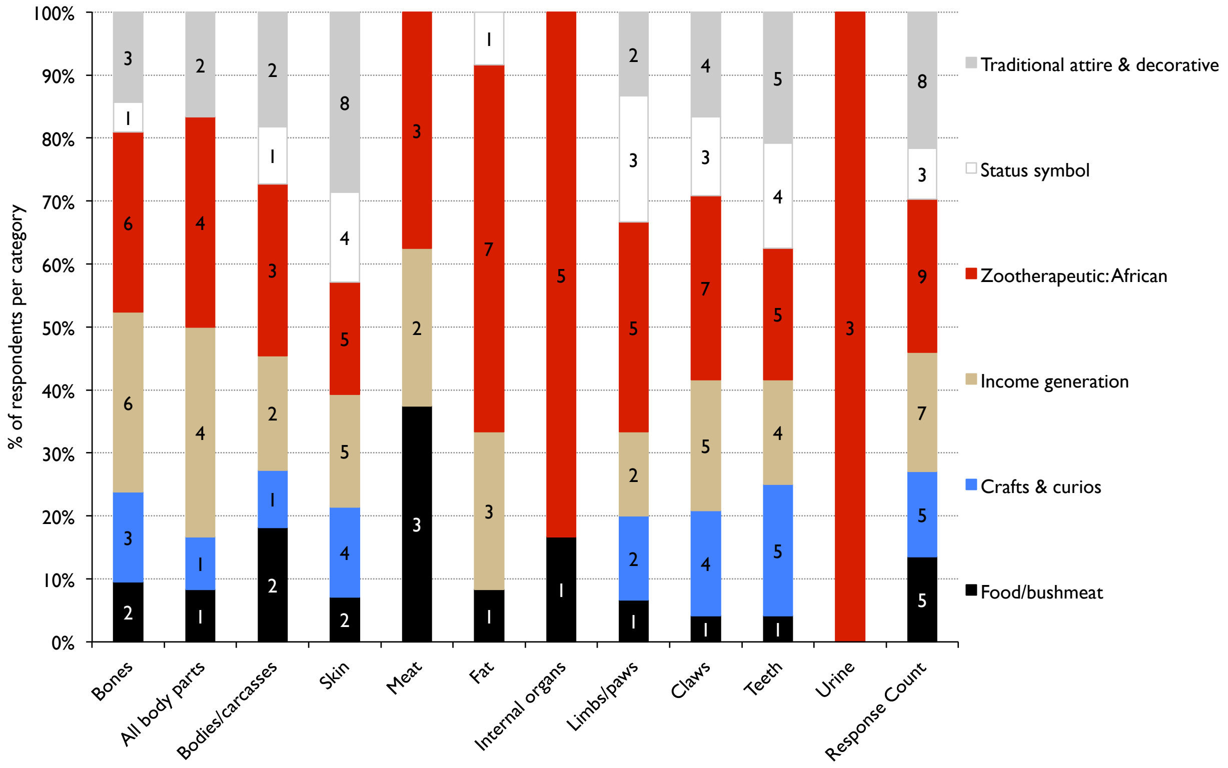

Fig. I. Reasons for domestic trade in South Africa

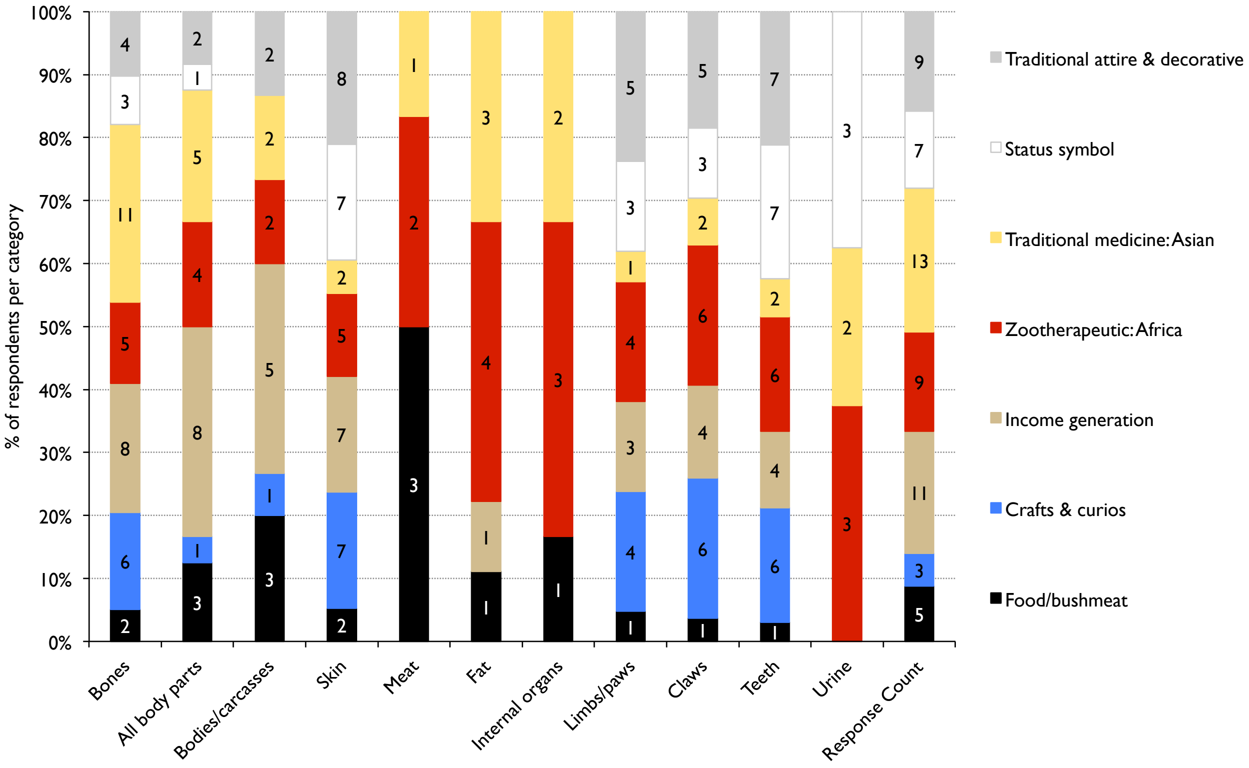

Fig. J Reasons for international trade in South Africa

The results for the perceived legality of trade are excluded from the results, but the explanatory responses are captured for South Africa in the comments of Question 15. South Africa was the only country in which 'mostly legal' trade was believed to occur (in the acquisition of the parts and/or their trade). The general perception was that, although known to exist, it was hard to find *documented* instances of illegal trade. For example, a North West Province government official wrote *"There are so many legally obtainable captive lions that were hunted that they will dominate* [over] *illegal sources of lion at least in NW (North West), though we are seeing trend of lions just dying at young age so it is suspicious...Even those with vet reports, most common claim is pneumonia as cause of death...I refer to captive ones that weren't hunted. But when it comes to body parts and skins much of the trade north of South Africa is illegal"*. Another respondent (Resp't #21) wrote that while the trade was mostly legal, "*nobody checks on the acquisition of the body parts, the ethical and humane killing of the animals and the welfare issues around lions in ex situ situations*".

Further to the above perceived legality of the body parts trade, the trade in lion bones was generally perceived by respondents to be illegal or mostly illegal across all range states and regions, with few respondents (12%) considering it to be entirely legal (except in South Africa where the proportion was higher). The respondents' comments in the open-ended section of this question address some of the issues, rumours and concerns around the legality of the trade that apply across all range states. Five respondents from Southern Africa wrote the following: (1) Resp't #12 (Mozambique): *"Sport hunting operators are required to burn or bury skeletons. On occasion skeletons and bones have been stolen. This is illegal. But it is not common – only 2-3 events in 12 years"*; (2) Resp't #15 (Namibia): *"The only legal trade I know of is sport hunting trophies. However, I am not sure whether Namibia has any specific laws about trading bones or body parts, other than that killing a protected species without declaring it and selling any part of it without a permit of is illegal. I am also not sure about what happens to the non-exported body parts of lions that are trophy hunted"*; (3) Resp't #27 (Zimbabwe & South Africa): *"Contraventions of CITES & other export requirements render international trade in lion bone from Zimbabwe mostly illegal. Local consumption of body parts for traditional meds...is in my estimation mostly legal in RSA and Zimbabwe; targeting of lion parts for traditional use in RSA is mostly illegal"*; (4) Resp't #3 (South Africa): *"Captive lion bone and body parts can be legally exported from South Africa. Wild lion bones find their way via the "legal"*

captive lion bone trade (speculative)"; (5) Resp't #2 (South Africa): *"Exporting of canned hunted lion bones is legal in South Africa, but there is persistent speculation that free-range lion bones are finding their way through this legal rout* (sic) *which is illegal"*.

South Africa is unique in Africa in that captive-bred lions, and not wild-sourced individuals, are acknowledged to be the primary source of the body parts (Fig. 6). Some parts become available from wild trophy hunted, problem and poached lions – but these latter incidences are sporadic. Consequently, the domestic trade was largely considered to be having little or no effect on wild populations (50% responses combined, Fig. 7). Only 25% of respondents said that the impact was medium to high, however most respondents felt that the impact on wild lion populations was still unknown (75% of responses, Fig. 7).
