# Supplementary material for: Questionnaire survey of the pan-African trade in lion body parts
Source: PLoS One. 2017 Oct 26;12(10):e0187060. doi: 10.1371/journal.pone.0187060 (PMC5658145; doi:10.1371/journal.pone.0187060)
Supplement: S1 Fig — (PDF) [file pone.0187060.s005.pdf]

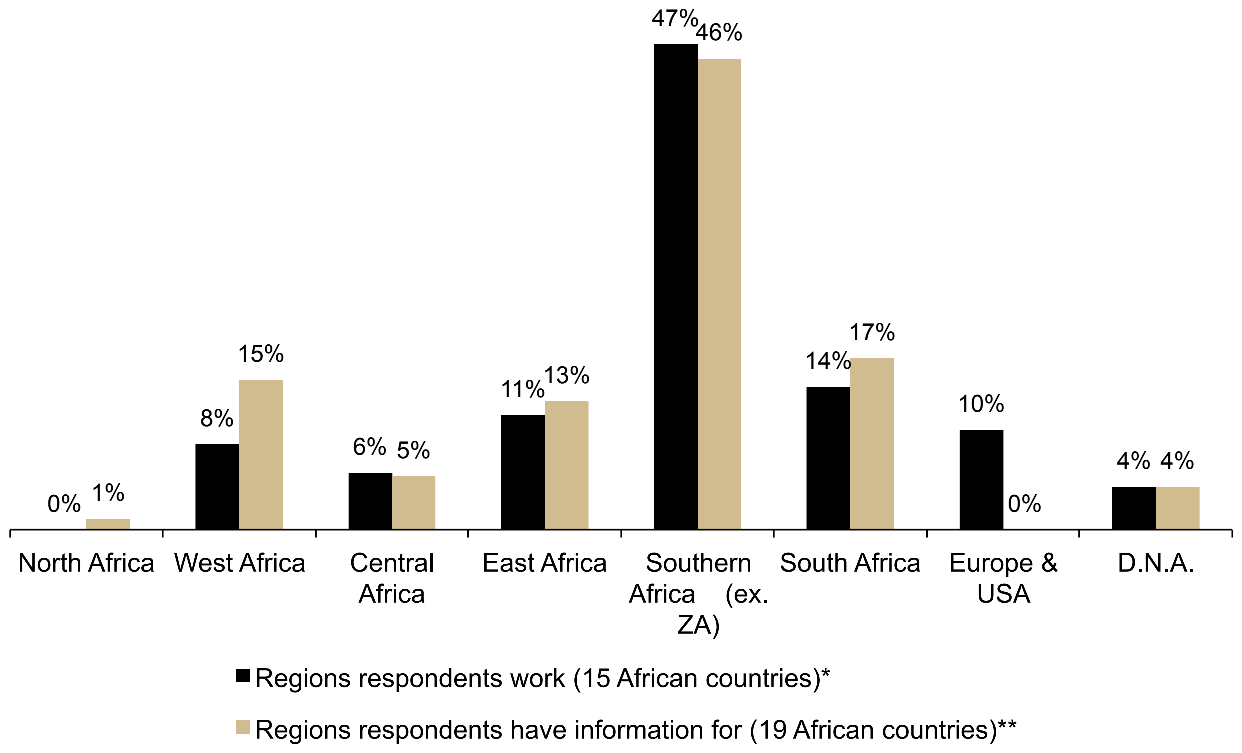

S1 Fig. Sub-regions in which respondents worked, and for which they had information on lions (answers include multiple responses). (D.N.A. = Did Not Answer; Ex. ZA = Excludes South Africa). African regions defined by Bauer et al [3]. [See full list of countries in Fig 2]. (Answers correspond to survey Questions 3 and 7)
