# Supplementary material for: Questionnaire survey of the pan-African trade in lion body parts
Source: PLoS One. 2017 Oct 26;12(10):e0187060. doi: 10.1371/journal.pone.0187060 (PMC5658145; doi:10.1371/journal.pone.0187060)
Supplement: S2 Fig — (PDF) [file pone.0187060.s006.pdf]

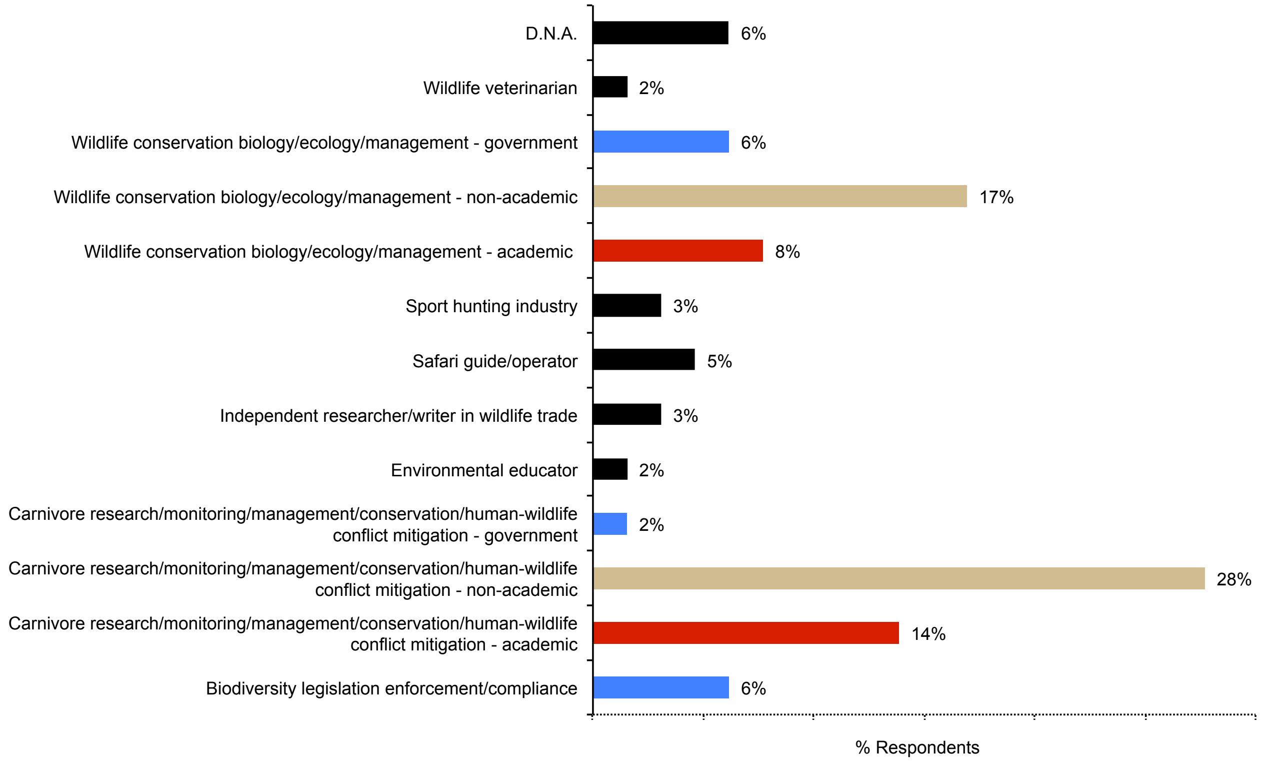

S2 Fig. Respondents' area of expertise and/or occupation and/or type of employment (Survey question 5) (D.N.A. = Did Not Answer) (Bars in blue, beige and red show that respondents worked for government, non-academic and academic institutions respectively)
