# Supplementary material for: Questionnaire survey of the pan-African trade in lion body parts
Source: PLoS One. 2017 Oct 26;12(10):e0187060. doi: 10.1371/journal.pone.0187060 (PMC5658145; doi:10.1371/journal.pone.0187060)
Supplement: S3 Fig — (PDF) [file pone.0187060.s007.pdf]

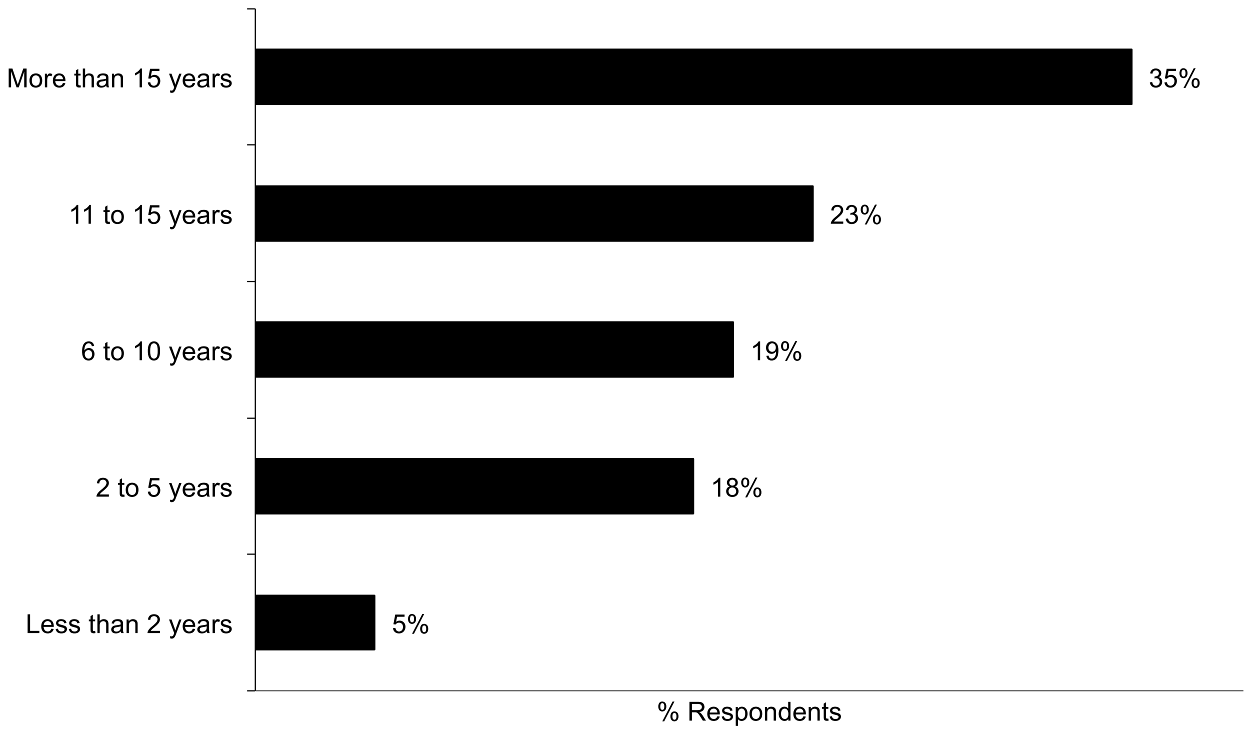

S3 Fig. Number of years respondents had been involved in lion conservation or allied wildlife matters (D.N.A. = Did Not Answer) (Survey question 6)
