# Supplementary material for: Questionnaire survey of the pan-African trade in lion body parts
Source: PLoS One. 2017 Oct 26;12(10):e0187060. doi: 10.1371/journal.pone.0187060 (PMC5658145; doi:10.1371/journal.pone.0187060)
Supplement: S4 Fig — The perceived impact of the domestic trade in lion (A) body parts (sub-regional), (B) bones (sub-regional), (C) body parts (per nominated range state), (D) bones (per nominated range state). (PDF) [file pone.0187060.s008.pdf]

S4 Fig. The perceived impact of the **domestic** trade in lion: (A) body parts (sub-regional), (B) bones (sub-regional), (C) body parts (per nominated range state), and (D) bones (per nominated range state). The Y-axis represents the percent responses per region, whereas numbers inside the histogram blocks are the number of responses. 'Total responses' is greater than the number of respondents because the questions' comment sections permitted people to elaborate on the countries/regions that their answers pertained to) (Excl. ZA = excluding South Africa)

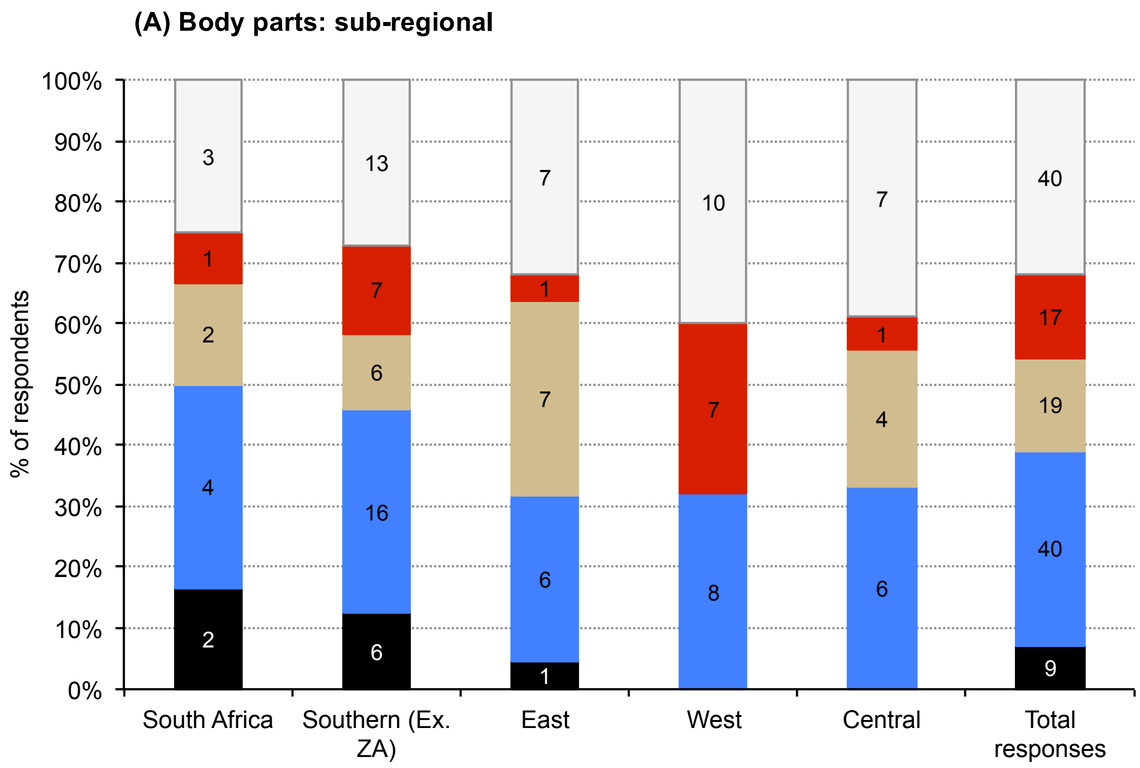

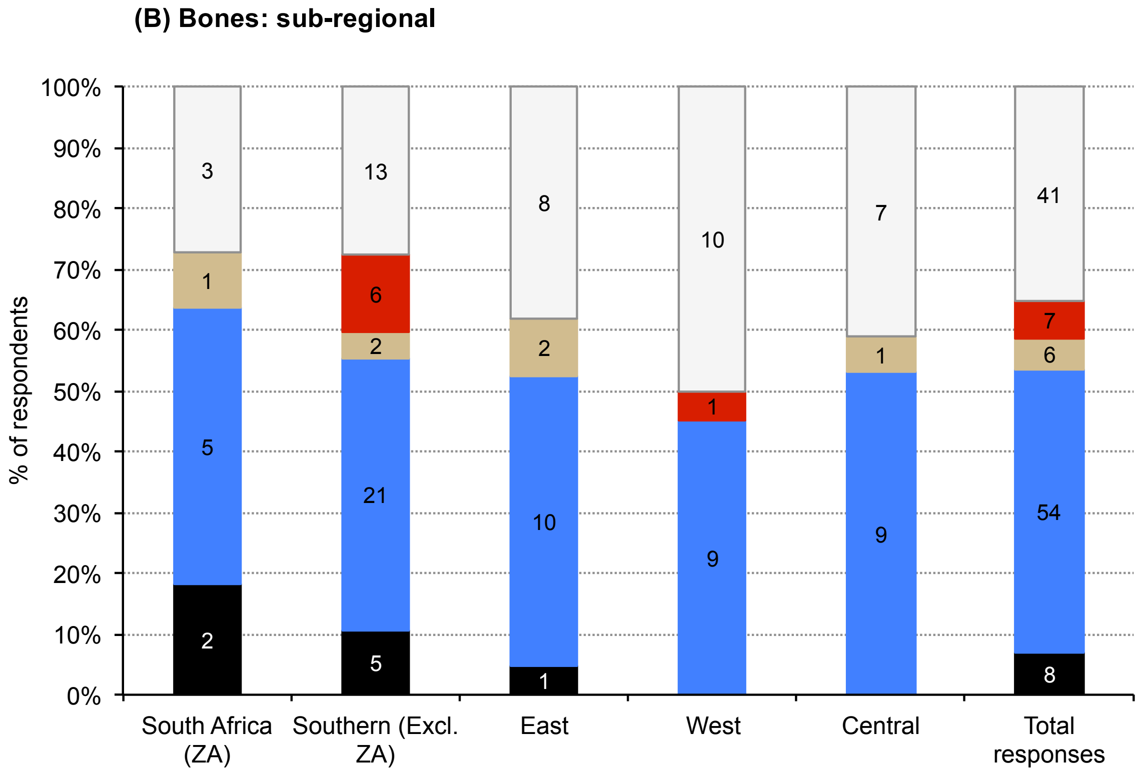

(C) Body parts: per nominated range state

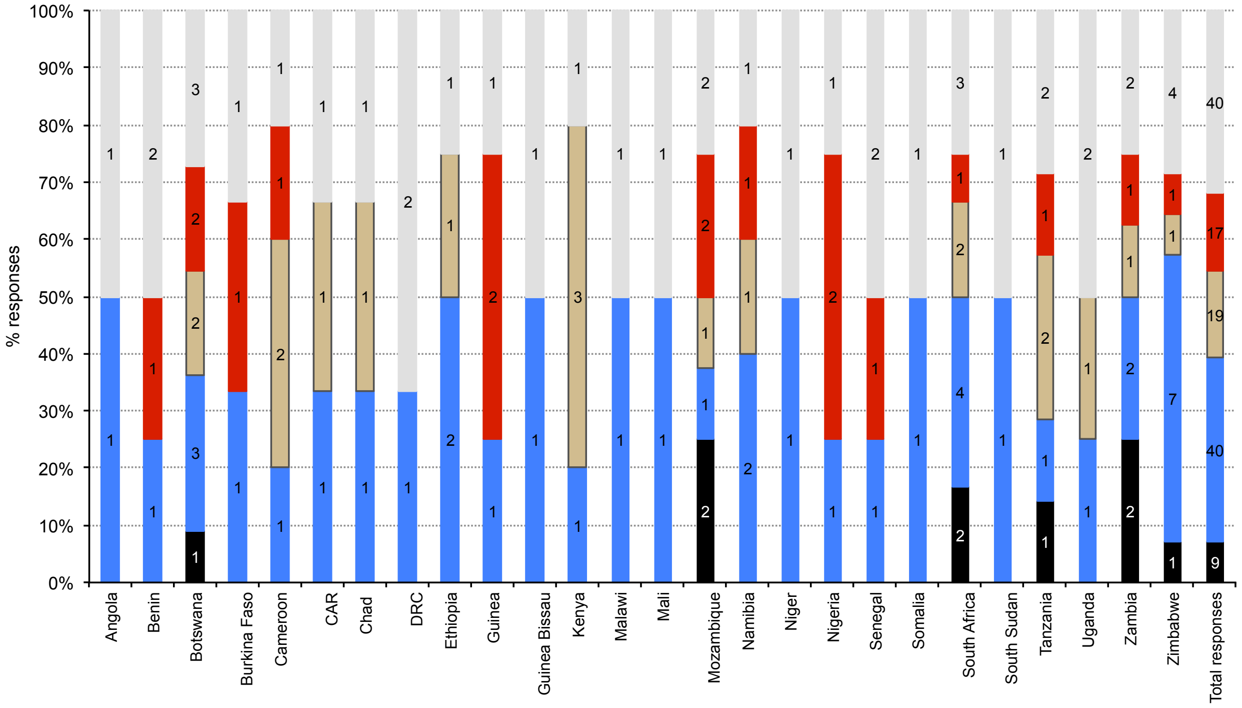

(D) Bones: per nominated range state

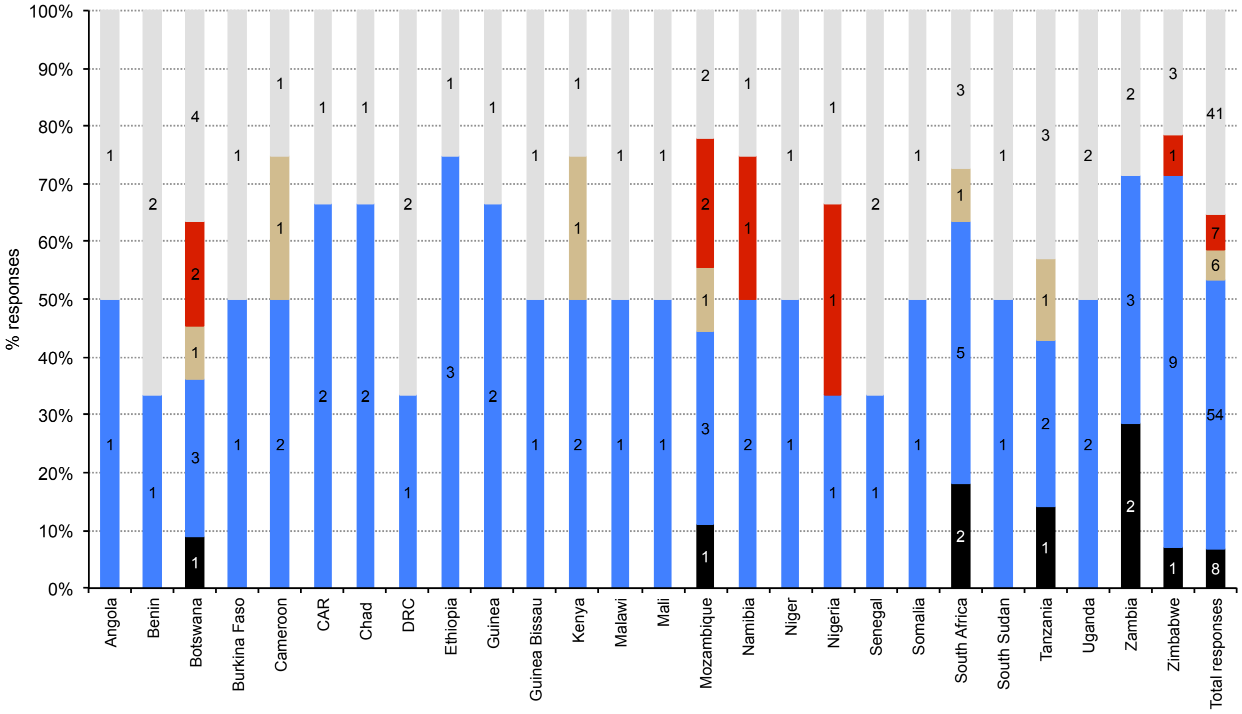

■ No impact ■ Low impact ■ Medium impact ■ High impact ■ Unknown impact
