# Supplementary material for: Questionnaire survey of the pan-African trade in lion body parts
Source: PLoS One. 2017 Oct 26;12(10):e0187060. doi: 10.1371/journal.pone.0187060 (PMC5658145; doi:10.1371/journal.pone.0187060)
Supplement: S6 Fig — (PDF) [file pone.0187060.s010.pdf]

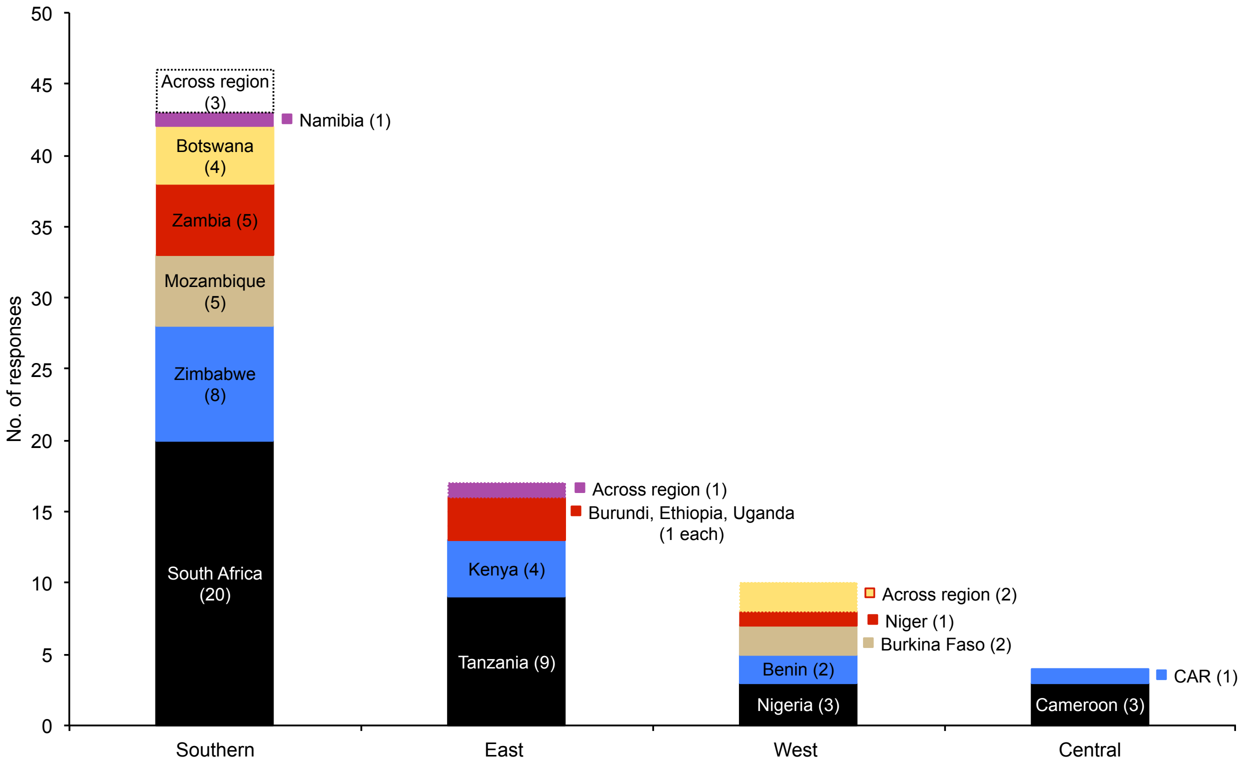

S6 Fig. Informant opinion on which African Lion range states are the most important in which to conduct studies vis-à-vis the trade in body parts. (Answers correspond to survey Question 25) (See also Fig. 2)
